# Perioperative penpulimab-based combination therapy in patients with resectable non-small cell lung cancer (ALTER-L043): an open-label, multicenter, randomized, phase II trial

**DOI:** 10.1038/s41392-025-02544-w

**Published:** 2026-01-16

**Authors:** Meng Wang, Weiran Liu, Hongbo Guo, Hao Long, Bentong Yu, Guofang Zhao, Jun Wu, Dongsheng Yue, Xiaoliang Zhao, Chenguang Li, Lianmin Zhang, Shengguang Wang, Qiang Zhang, Zhenfa Zhang, Changli Wang

**Affiliations:** 1 Department of Lung Cancer, Tianjin Medical University Cancer Institute and Hospital, Tianjin, China; 2 Department of Anesthesiology, Tianjin Medical University Cancer Institute and Hospital, Tianjin, China; 3 Department of Thoracic Surgery, Shandong Cancer Hospital and Institute, Jinan, China; 4 Department of Thoracic Surgery, Sun Yat-sen University Cancer Center, Guangzhou, China; 5 Department of Thoracic Surgery, The First Affiliated Hospital of Nanchang University, Nanchang, China; 6 Department of Thoracic Surgery, Ningbo No.2 Hospital, Ningbo, China; 7 Department of Thoracic Surgery, Hainan Cancer Hospital, The Affiliated Cancer Hospital of Hainan Medical, Haikou, China

**Keywords:** Lung cancer, Clinical trials

## Abstract

Although perioperative immunotherapy combined with neoadjuvant chemotherapy has improved the clinical outcomes of patients with resectable non-small cell lung cancer (NSCLC), the optimal combination strategy remains unknown. This multicenter, open-label, randomized, phase II trial (ALTER-L043; NCT04846634) evaluated the efficacy and safety of perioperative penpulimab plus anlotinib with or without neoadjuvant chemotherapy in patients with resectable NSCLC. Eligible patients were randomly assigned (1:1:1) to receive 3–4 cycles of neoadjuvant penpulimab (200 mg on day 1) plus anlotinib (12 mg on days 1–14) and chemotherapy, penpulimab plus chemotherapy, or penpulimab plus anlotinib, followed by surgery and matching adjuvant therapy. The primary endpoint was the investigator-assessed major pathologic response (MPR) rate. Between December 3, 2021, and January 23, 2024, 90 patients were randomly assigned to the penpulimab plus anlotinib and chemotherapy (n = 30), penpulimab plus chemotherapy (n = 30), or penpulimab plus anlotinib (n = 30) groups. Definitive surgery was performed in 92.6%, 89.7%, and 70.0% of patients, respectively. Among those who underwent surgery, the MPR and pathological complete response rates were 76.0% (95% CI 54.9–90.6) and 52.0% (95% CI 31.3–72.2), respectively, in the penpulimab plus anlotinib and chemotherapy group; 57.7% (95% CI 36.9–76.7) and 50.0% (95% CI 29.9–70.1), respectively, in the penpulimab plus chemotherapy group; and 52.4% (95% CI 29.8–74.3) and 38.1% (95% CI 18.1–61.6), respectively, in the penpulimab plus anlotinib group. Across all treatment phases, the incidences of grade ≥3 treatment-related adverse events were 26.7%, 20.0%, and 30.0%, respectively. Penpulimab plus anlotinib with or without neoadjuvant chemotherapy demonstrated promising efficacy and a manageable safety profile in patients with resectable NSCLC, suggesting its potential as a viable perioperative treatment option.

## Introduction

Non-small cell lung cancer (NSCLC) is the predominant pathological subtype of lung cancer, accounting for 80% to 85% of all cases.^1^ For patients with early-stage NSCLC, surgical resection remains the mainstay treatment.^2^ However, 30% to 77% of patients still experience recurrence after surgery, resulting in long-term survival of less than 50%.^3^ Although perioperative (neoadjuvant or adjuvant) chemotherapy has significantly improved the survival of patients with NSCLC, the benefit remains modest, with an absolute survival improvement of only 5% at 5 years compared with that of surgery alone, emphasizing the need for new treatment options in this setting.^4^

In recent years, the revolutionary breakthrough of immunotherapy in advanced NSCLC has inspired the search for a place in the perioperative setting of resectable NSCLC.^5,6^ As the first published phase III analysis of neoadjuvant chemoimmunotherapy, the CheckMate 816 trial revealed that, compared with chemotherapy alone, nivolumab plus chemotherapy significantly improved the median event-free survival (EFS; 31.6 vs 20.8 months, *p* = 0.005) and pathological complete response (pCR; 24.0% vs 2.2%, *p* < 0.001) rates in patients with resectable stage IB-IIIA NSCLC.^7^ Compared with neoadjuvant chemotherapy alone, several phase III clinical trials revealed significant improvements in EFS and pCR in patients with resectable NSCLC treated with perioperative immune checkpoint inhibitors (ICIs) plus neoadjuvant chemotherapy^.^^8–13^ Despite exciting advances, the optimal ICI-based combination strategy and the optimal duration of treatment in the perioperative setting remain unknown.

Penpulimab is a novel humanized fragment crystallizable (Fc)-engineered IgG1 anti-programmed cell death-1 (PD-1) monoclonal antibody with improved efficacy and a low incidence of immune-related adverse events (irAEs).^14^ It received approval from the China National Medical Products Administration (NMPA) in August 2021 for the treatment of patients with relapsed or refractory classical Hodgkin lymphoma who had previously received at least two lines of chemotherapy.^15,16^ Anlotinib, an oral multitarget tyrosine kinase inhibitor (TKI), has shown promising efficacy and an acceptable safety profile in multiple solid tumors, especially as a third-line or subsequent treatment for advanced NSCLC.^17,18^ Preclinical studies have shown that anlotinib can enhance the infiltration of innate immune cells, facilitate tumor vessel normalization, and restore the immunostimulatory microenvironment, conferring potentially synergistic antitumor activities when combined with immunotherapy.^19–21^ Additionally, anlotinib prolongs the retention time and distribution of other antitumor drugs by reducing extracellular matrix (ECM) stiffness and reshaping the physiological and physical characteristics of the TME.^22^ Furthermore, the combination of penpulimab plus anlotinib appears to be effective in prospective clinical trials against NSCLC, head and neck squamous cell carcinoma, pancreatic cancer, and hepatocellular carcinoma.^23–26^ Nevertheless, the application of this combination regimen for resectable NSCLC in the perioperative setting remains unknown.

Thus, this multicenter, randomized, phase II trial (ALTER-L043) was conducted to evaluate the efficacy and safety of perioperative penpulimab plus anlotinib with or without neoadjuvant chemotherapy in patients with resectable NSCLC.

## Results

### Patient characteristics

**Fig. 1:**
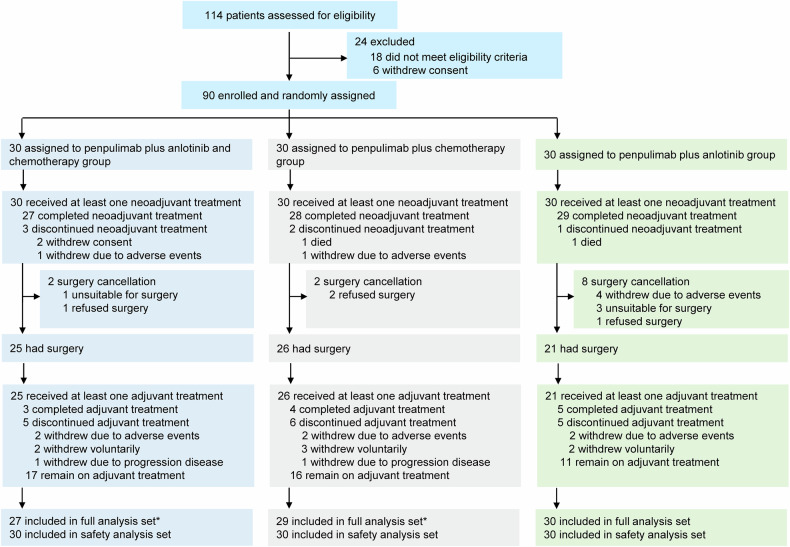
Trial profile of the ALTER-L043 study. ^*^Three patients who did not undergo tumor evaluation after one cycle of treatment were excluded from the full analysis set

From December 3, 2021, to January 23, 2024, 114 patients were screened for eligibility, 90 of whom were randomly assigned to receive neoadjuvant penpulimab plus anlotinib and chemotherapy (n = 30), penpulimab plus chemotherapy (n = 30), or penpulimab plus anlotinib (n = 30; Fig. 1). All 90 patients were included in the safety analysis set (SS), whereas 3 patients in the penpulimab plus anlotinib and chemotherapy group and 1 patient in the penpulimab plus chemotherapy group were excluded from the full analysis set (FAS; Fig. 1). Baseline demographics and disease characteristics were generally balanced among the three treatment groups in the FAS population (Table 1). The median age was 66 years (range, 46–69) in the penpulimab plus anlotinib and chemotherapy group, 58 years (range, 52–70) in the penpulimab plus chemotherapy group, and 62 years (range, 53–71) in the penpulimab plus anlotinib group. The proportions of patients with squamous NSCLC in the three treatment groups were 77.8% (21/27), 75.9% (22/29), and 86.7% (26/30), respectively.

**Table 1:** Baseline demographics and disease characteristics of patients in the full analysis set

	Penpulimab plus anlotinib and chemotherapy group (n = 27)	Penpulimab plus chemotherapy group (n = 29)	Penpulimab plus anlotinib group (n = 30)	*P* value
Age, years				0.311
Median (range)	66 (46–69)	58 (52–70)	62 (53–71)	
<65	17 (63.0)	18 (62.1)	17 (56.7)	
≥65	10 (37.0)	11 (37.9)	13 (43.3)	
Gender				0.545
Male	25 (92.6)	28 (96.6)	28 (93.3)	
Female	2 (7.4)	1 (3.5)	2 (6.7)	
Smoking status				0.827
Never	3 (11.1)	4 (13.8)	6 (20.0)	
Former	20 (74.1)	20 (69.0)	22 (73.3)	
Current	4 (14.8)	5 (17.2)	2 (6.7)	
ECOG performance status				0.980
0	14 (51.9)	17 (58.6)	19 (63.3)	
1	13 (48.2)	12 (41.4)	11 (36.7)	
Pathology				0.615
Squamous	21 (77.8)	22 (75.9)	26 (86.7)	
Nonsquamous	6 (22.2)	7 (24.1)	3 (10.0)	
Unknown	0	0	1 (3.3)	
Clinical stage				0.131
IIB	11 (40.7)	19 (65.5)	14 (46.7)	
IIIA	12 (44.4)	9 (31.0)	8 (26.7)	
IIIB	4 (14.8)	1 (3.5)	8 (26.7)	
Node stage				0.779
N0	5 (18.5)	8 (27.6)	4 (13.3)	
N1	9 (33.3)	11 (37.9)	13 (43.3)	
N2	13 (48.2)	10 (34.5)	13 (43.3)	
Tumor PD-L1 expression				0.881
<1%	8 (29.6)	6 (20.7)	10 (33.3)	
≥1%	9 (33.3)	13 (44.8)	3 (10.0)	
Not evaluable	10 (37.0)	10 (34.5)	17 (56.7)	

Data are n (%) unless otherwise specified

*ECOG* Eastern Cooperative Oncology Group, *PD-L1* programmed cell death-ligand 1

### Treatment

At the data cutoff (January 17, 2025), the median duration of follow-up was 14.0 months (95% confidence intervals [CI] 11.9–14.8). Among the 86 patients in the FAS, 27 (100.0%) of the 27 in the penpulimab plus anlotinib and chemotherapy group, 28 (96.6%) of the 29 in the penpulimab plus chemotherapy group, and 29 (96.7%) of the 30 in the penpulimab plus anlotinib group completed the planned neoadjuvant treatment. The reasons for the discontinuation of neoadjuvant treatment are provided in Fig. 1.

After neoadjuvant treatment, 25 (92.6%) of 27 patients in the penpulimab plus anlotinib and chemotherapy group, 26 (89.7%) of 29 patients in the penpulimab plus chemotherapy group, and 21 (70.0%) of 30 patients in the penpulimab plus anlotinib group underwent definitive surgery (Fig. 1). The detailed reasons for surgical cancellation are provided in Supplementary Table 1. Adjuvant treatment was administered to all patients who underwent definitive surgery. At the data cutoff, 63.0% (17/27), 55.2% (16/29), and 36.7% (11/30) of patients remained on adjuvant treatment, respectively.

### Efficacy

**Fig. 2:**
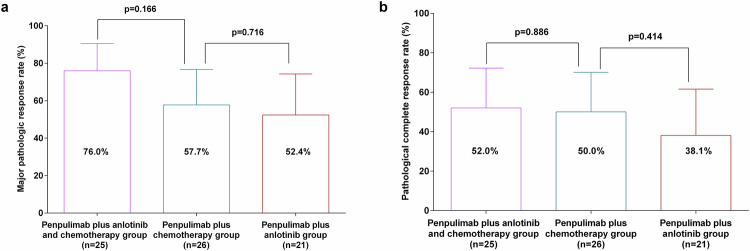
Pathological response in patients who underwent definitive surgery. **a** Investigator-assessed major pathologic response rate in the three groups. **b** Investigator-assessed pathological complete response rate in the three groups

Among patients who underwent definitive surgery, major pathologic response (MPR) occurred in 19 (76.0% [95% CI 54.9–90.6]) of 25 patients in the penpulimab plus anlotinib and chemotherapy group, 15 (57.7% [95% CI 36.9–76.7]) of 26 patients in the penpulimab plus chemotherapy group, and 11 (52.4% [95% CI 29.8–74.3]) of 21 patients in the penpulimab plus anlotinib group (Fig. 2a). Compared with that in the penpulimab plus chemotherapy group, the MPR rate difference was 18.3% (95% CI −7.9–42.2; p = 0.166) for the penpulimab plus anlotinib and chemotherapy group and 5.3% (95% CI −32.8–22.7; *p* = 0.716) for the penpulimab plus anlotinib group. The MPR appeared to improve with penpulimab plus anlotinib and chemotherapy across key subgroups (Table 2). Patients with squamous NSCLC (79.0% [95% CI 54.4–94.0] vs 55.0% [95% CI 31.5–76.9] vs 50.0% [95% CI 26.0–74.0]) and those with stage IIIA/IIIB disease (82.4% [95% CI 56.6–96.2] vs 55.6% [95% CI 21.2–86.3] vs 50.0% [95% CI 21.1–78.9]) in the penpulimab plus anlotinib and chemotherapy group presented numerically higher MPR rates than did those in the penpulimab plus chemotherapy and penpulimab plus anlotinib groups.

**Table 2:** Subgroup analyses for the MPR in patients who underwent definitive surgery

	Penpulimab plus anlotinib and chemotherapy group (n = 25)		Penpulimab plus chemotherapy group (n = 26)		Penpulimab plus anlotinib group (n = 21)	
	n/N	MPR rate	n/N	MPR rate	n/N	MPR rate
Age, years						
<65	12/15	80.0 (51.9–95.7)	8/18	44.4 (21.5–69.2)	7/12	58.3 (27.7–84.8)
≥65	7/10	70.0 (34.8–93.3)	7/8	87.5 (47.4–99.7)	4/9	44.4 (13.7–78.8)
Gender						
Male	18/23	78.3 (56.3–92.5)	15/25	60.0 (38.7–78.9)	10/20	50.0 (27.2–72.8)
Female	1/2	50.0 (1.3–98.7)	0/1	0	1/1	100.0 (2.5–100.0)
Smoking status						
Never	2/3	66.7 (9.4–99.2)	0/3	0	2/4	50.0 (6.8–93.2)
Former	15/19	79.0 (54.4–94.0)	13/18	72.2 (46.5–90.3)	8/16	50.0 (24.7–75.4)
Current	2/3	66.7 (9.4–99.2)	2/5	40.0 (5.3–85.3)	1/1	100.0 (2.5–100.0)
ECOG performance status						
0	10/14	71.4 (41.9–91.6)	10/16	62.5 (35.4–84.8)	7/16	43.8 (19.8–70.1)
1	9/11	81.8 (48.2–97.7)	5/10	50.0 (18.7–81.3)	4/5	80.0 (28.4–99.5)
Pathology						
Squamous	15/19	79.0 (54.4–94.0)	11/20	55.0 (31.5–76.9)	9/18	50.0 (26.0–74.0)
Nonsquamous	4/6	66.7 (22.3–95.7)	4/6	66.7 (22.3–95.7)	2/3	66.7 (9.4–99.2)
Clinical stage						
IIB	5/8	62.5 (24.5–91.5)	10/17	58.8 (32.9–81.6)	5/9	55.6 (21.2–86.3)
IIIA/IIIB	14/17	82.4 (56.6–96.2)	5/9	55.6 (21.2–86.3)	6/12	50.0 (21.1–78.9)
Node stage						
N0	2/4	50.0 (6.8–93.2)	5/6	83.3 (35.9–99.6)	1/3	33.3 (0.8–90.6)
N1	6/8	75.0 (34.9–96.8)	5/11	45.5 (16.8–76.6)	5/9	55.6 (21.2–86.3)
N2	11/13	84.6 (54.6–98.1)	5/9	55.6 (21.2–86.3)	5/9	55.6 (21.2–86.3)
Tumor PD-L1 expression						
<1%	4/8	50.0 (15.7–84.3)	3/5	60.0 (14.7–94.7)	2/9	22.2 (2.8–60.0)
≥1%	6/8	75.0 (34.9–96.8)	8/12	66.7 (34.9–90.1)	3/3	100.0 (29.2–100.0)
Unknown	9/9	100.0 (66.4–100.0)	4/9	44.4 (13.7–78.8)	6/9	66.7 (29.9–92.5)

Data are n or % (95% CI)

*MPR* major pathologic response, *ECOG* Eastern Cooperative Oncology Group, *PD-L1* programmed cell death-ligand 1

The pCR rate was 52.0% (13/25; 95% CI 31.3–72.2) with penpulimab plus anlotinib and chemotherapy, 50.0% (13/26; 95% CI 29.9–70.1) with penpulimab plus chemotherapy, and 38.1% (8/21; 95% CI 18.1–61.6) with penpulimab plus anlotinib (Fig. 2b). Compared with the penpulimab plus chemotherapy group, no significant difference in the pCR rate was observed in the penpulimab plus anlotinib and chemotherapy group (difference: 2.0% [95% CI −24.9 to 28.6]; p = 0.886) or in the penpulimab plus anlotinib group (difference: 11.9% [95% CI −38.4 to 16.7]; p = 0.414). Subgroup analyses for pCR are shown in supplementary Table 2. Among patients with squamous NSCLC, the pCR rate was numerically greater in the penpulimab plus anlotinib and chemotherapy group (57.9% [95% CI 33.5–79.8]) than in the penpulimab plus chemotherapy (50.0% [95% CI 27.2–72.8]) and penpulimab plus anlotinib groups (33.3% [95% CI 13.3–59.0]). Similarly, in patients with stage IIIA/IIIB disease, the pCR rates were 52.9% (95% CI 27.8–77.0), 44.4% (95% CI 13.7–78.8), and 25.0% (95% CI 5.5–57.2), respectively.

An objective response was achieved in 21 (77.8% [95% CI 57.7–91.4]) of 27 patients in the penpulimab plus anlotinib and chemotherapy group, 17 (58.6% [95% CI 38.9–76.5]) of 29 patients in the penpulimab plus chemotherapy group, and 19 (63.3% [95% CI 43.9–80.1]) of 30 patients in the penpulimab plus anlotinib group prior to surgery (Table 3).

**Table 3:** Tumor response prior to surgery in the full analysis set population

	Penpulimab plus anlotinib and chemotherapy group (n = 27)	Penpulimab plus chemotherapy group (n = 29)	Penpulimab plus anlotinib group (n = 30)
Best overall response			
Complete response	0	0	0
Partial response	21 (77.8)	17 (58.6)	19 (63.3)
Stable disease	6 (22.2)	12 (41.4)	11 (36.7)
Progressive disease	0	0	0
Objective response rate, n (% [95% CI])	21 (77.8 [57.7–91.4])	17 (58.6 [38.9–76.5])	19 (63.3 [43.9–80.1])
Disease control rate, n (% [95% CI])	27 (100.0 [87.2–100.0])	29 (100.0 [88.1–100.0])	30 (100.0 [88.4–100.0])

Data are n (%) unless otherwise specified. *CI* confidence interval

At the data cutoff, 1 (3.7%) of 27 patients in the penpulimab plus anlotinib and chemotherapy group, 4 (13.8%) of 29 patients in the penpulimab plus chemotherapy group, and 6 (20.0%) of 30 patients in the penpulimab plus anlotinib group experienced EFS events. The median EFS was not reached in each group (supplementary Fig. 1a). The 12-month EFS rates were 94.1% (95% CI 65.0–99.2), 89.3% (95% CI 63.2–97.2), and 73.6% (95% CI 49.9–87.3), respectively. The post hoc 24-month EFS rates were 94.1% (95% CI 65.0–99.2), 53.6% (95% CI 9.0–85.0), and 73.6% (95% CI 49.9–87.3), respectively. Subgroup analyses for 6-month and 12-month EFS are provided in supplementary Table 3.

At the time of data analysis, one death occurred in both the penpulimab plus chemotherapy group and the penpulimab plus anlotinib group. The median overall survival (OS) was not reached (supplementary Fig. 1b). The 12-month OS rate was 100.0% (95% CI 100.0–100.0) with penpulimab plus anlotinib and chemotherapy, 94.7% (95% CI 68.1–99.2) with penpulimab plus chemotherapy, and 96.7% (95% CI 78.6–99.5) with penpulimab plus anlotinib.

### Safety

Across all treatment phases, treatment-emergent AEs (TEAEs) of any grade occurred in 26 (86.7%) of the 30 patients in the penpulimab plus anlotinib and chemotherapy groups, 29 (96.7%) of the 30 patients in the penpulimab plus chemotherapy group, and 28 (93.3%) of the 30 patients in the penpulimab plus anlotinib group; of these, 73.3% (22/30), 60.0% (18/30), and 76.7% (23/30), respectively, were treatment related (supplementary Tables 4 and 5). Grade ≥3 treatment-related AEs (TRAEs) were reported in 26.7% (8/30), 20.0% (6/30), and 30.0% (9/30) of patients in the three groups, respectively. The most common grade ≥3 TRAEs were hypertension (2 [6.7%] in the penpulimab plus anlotinib and chemotherapy group *vs* 0 [0.0%] in the penpulimab plus chemotherapy group *vs* 4 [13.3%] in the penpulimab plus anlotinib group), nausea (1 [3.3%] vs 1 [3.3%] vs 0 [0.0%]), anorexia (1 [3.3%] vs 0 [0.0%] vs 1 [3.3%]), anemia (1 [3.3%] vs 1 [3.3%] vs 0.0%]), and hand‒foot syndrome (2 [6.7%] vs 0 [0.0%] vs 0.0%]; Table 4).

**Table 4:** Treatment-related adverse events occurring in ≥ 5% of patients in the safety analysis set population

	Penpulimab plus anlotinib and chemotherapy group (n = 30)			Penpulimab plus chemotherapy group (n = 30)			Penpulimab plus anlotinib group (n = 30)		
	Any grade	Grade 1–2	Grade 3–5	Any grade	Grade 1–2	Grade 3–5	Any grade	Grade 1–2	Grade 3–5
Nausea	9 (30.0)	8 (26.7)	1 (3.3)	3 (10.0)	2 (6.7)	1 (3.3)	0	0	0
Elevated ALT	7 (23.3)	7 (23.3)	0	2 (6.7)	2 (6.7)	0	0	0	0
Hypertension	6 (20.0)	4 (13.3)	2 (6.7)	0	0	0	7 (23.3)	3 (10.0)	4 (13.3)
Anorexia	5 (16.7)	4 (13.3)	1 (3.3)	3 (10.0)	3 (10.0)	0	1 (3.3)	0	1 (3.3)
Elevated AST	4 (13.3)	4 (13.3)	0	2 (6.7)	2 (6.7)	0	0	0	0
Rash	4 (13.3)	4 (13.3)	0	5 (16.7)	4 (13.3)	1 (3.3)	7 (23.3)	7 (23.3)	0
Weakness	4 (13.3)	4 (13.3)	0	3 (10.0)	3 (10.0)	0	5 (16.7)	5 (16.7)	0
Arthralgia	3 (10.0)	2 (6.7)	1 (3.3)	0	0	0	2 (6.7)	2 (6.7)	0
Thrombocytopenia	3 (10.0)	2 (6.7)	1 (3.3)	0	0	0	0	0	0
Oral ulceration	2 (6.7)	2 (6.7)	0	1 (3.3)	1 (3.3)	0	3 (10.0)	3 (10.0)	0
Vomiting	2 (6.7)	2 (6.7)	0	0	0	0	1 (3.3)	1 (3.3)	0
Hand-foot syndrome	2 (6.7)	0	2 (6.7)	0	0	0	0	0	0
Toothache	2 (6.7)	2 (6.7)	0	2 (6.7)	2 (6.7)	0	1 (3.3)	1 (3.3)	0
Hypothyroidism	2 (6.7)	2 (6.7)	0	1 (3.3)	1 (3.3)	0	3 (10.0)	3 (10.0)	0
Leukopenia	2 (6.7)	2 (6.7)	0	2 (6.7)	1 (3.3)	1 (3.3)	1 (3.3)	1 (3.3)	0
Neurological disorders	2 (6.7)	2 (6.7)	0	0	0	0	0	0	0
Pleural effusion	2 (6.7)	2 (6.7)	0	0	0	0	0	0	0
Alopecia	2 (6.7)	2 (6.7)	0	0	0	0	0	0	0
Diarrhea	2 (6.7)	2 (6.7)	0	1 (3.3)	1 (3.3)	0	2 (6.7)	2 (6.7)	0
Abdominal distension	2 (6.7)	2 (6.7)	0	0	0	0	0	0	0
Elevated TSH	2 (6.7)	2 (6.7)	0	0	0	0	1 (3.3)	1 (3.3)	0
Anemia	2 (6.7)	1 (3.3)	1 (3.3)	4 (13.3)	3 (10.0)	1 (3.3)	0	0	0
Oropharyngeal discomfort	1 (3.3)	1 (3.3)	0	2 (6.7)	1 (3.3)	0	2 (6.7)	2 (6.7)	0
Hemoptysis	1 (3.3)	1 (3.3)	0	0	0	0	3 (10.0)	2 (6.7)	1 (3.3)
Skin exfoliation	1 (3.3)	1 (3.3)	0	0	0	0	2 (6.7)	2 (6.7)	0
Dry skin	1 (3.3)	1 (3.3)	0	1 (3.3)	1 (3.3)	0	2 (6.7)	2 (6.7)	0
Gastrointestinal distention	1 (3.3)	1 (3.3)	0	2 (6.7)	2 (6.7)	0	0	0	0
Musculoskeletal pain	1 (3.3)	0	1 (3.3)	1 (3.3)	1 (3.3)	0	2 (6.7)	2 (6.7)	0
Fever	0	0	0	2 (6.7)	2 (6.7)	0	1 (3.3)	0	1 (3.3)
Oral mucositis	0	0	0	0	0	0	2 (6.7)	2 (6.7)	0
Postoperative wound complications	0	0	0	2 (6.7)	2 (6.7)	0	0	0	0
Hyperthyroidism	0	0	0	0	0	0	2 (6.7)	2 (6.7)	0

Data are n (%)

*ALT* alanine aminotransferase, *AST* aspartate aminotransferase, *TSH* thyroid-stimulating hormone

Serious AEs were observed in 8 (26.7%) of the 30 patients in the penpulimab plus anlotinib and chemotherapy group, 9 (30.0%) of the 30 patients in the penpulimab plus chemotherapy group, and 9 (30.0%) of the 30 patients in the penpulimab plus anlotinib group (supplementary Table 6); 23.3% (7/30), 26.7% (8/30), and 30.0% (9/30), respectively, were grade ≥3. The incidences of TRAEs leading to dose reduction or interruption in the penpulimab plus anlotinib and chemotherapy groups (9 [30.0%]) and the penpulimab plus anlotinib group (8 [26.7%]) were greater than those in the penpulimab plus chemotherapy group (3 [10.0%]). One patient (3.3%) in the penpulimab plus anlotinib plus chemotherapy group discontinued treatment because of grade 1 lacunar cerebral infarction (considered possibly related to anlotinib and unlikely related to penpulimab). Two patients (6.7%) in the penpulimab plus chemotherapy group discontinued treatment because of grade 3 immune-related hypothyroidism and hypophysitis. Four patients (13.3%) in the penpulimab plus anlotinib group discontinued treatment because of grade 3 fever and a grade 4 confusional state (both considered possibly related to both anlotinib and penpulimab) and grade 3 hemoptysis and myocardial infarction (both considered possibly related to anlotinib and unlikely related to penpulimab). No treatment-related deaths were observed across the three groups.

The incidence of immune-related AEs (irAEs) with penpulimab plus anlotinib and chemotherapy was greater than that with penpulimab plus chemotherapy and penpulimab plus anlotinib (11 [36.7%] vs 6 [20.0%] vs 7 [23.3%]; supplementary Table 7). These events were grade ≥3 in 23.3% (7/30), 10.0% (3/30), and 6.7% (2/30) of patients, respectively. In the penpulimab plus anlotinib and chemotherapy group, 7 patients experienced 9 grade ≥3 irAEs, including grade 3 arthralgia (lasting 5 days, no treatment interruption, resolved with analgesics), lacunar cerebral infarction (lasting 10 days, treatment delayed for 1 month), musculoskeletal pain (treatment interrupted and managed with glucocorticoids), anemia (no specific intervention), myasthenia (treatment interrupted), anorexia (treatment interrupted and managed with metoclopramide), hypertension (managed with antihypertensives), myocardial ischemia (managed with trimetazidine), and grade 4 myocardial infarction (lasting 1 month; resulting in treatment discontinuation).

## Discussion

To our knowledge, this multicenter, randomized, phase II study (ALTER-L043) provides the first analysis of perioperative ICI in combination with an antiangiogenic agent with or without neoadjuvant chemotherapy in patients with resectable NSCLC. In the present study, the addition of anlotinib to penpulimab plus chemotherapy yielded a numerically higher MPR (76.0% vs 57.7% vs 52.4%) than penpulimab plus chemotherapy or penpulimab plus anlotinib. The pCR rate (52.0% vs 50.0% vs 38.1%), ORR (77.8% vs 58.6% vs 63.3%), 12-month EFS rate (94.1% vs 89.3% vs 73.6%), and 12-month OS rate (100.0% vs 94.7% vs 96.7%) were comparable among the three treatment groups. Additionally, all three combination regimens demonstrated tolerable and manageable safety profiles, and no new safety signals were reported. Furthermore, definitive surgery (92.6% vs 89.7%) rates were similar between the penpulimab plus anlotinib and chemotherapy group and the penpulimab plus chemotherapy group, indicating that the addition of anlotinib to chemoimmunotherapy did not affect the feasibility of surgery.

Recent randomized phase III trials, including Neotorch, KEYNOTE-671, AEGEAN, RATIONALE-315, and CheckMate 77 T, have established the clinical benefits of perioperative ICI in combination with neoadjuvant chemotherapy for resectable NSCLC.^8–13^ In prior phase II/III trials, the MPR and pCR rates with perioperative ICI plus neoadjuvant chemotherapy ranged from 30.2% to 58.6% and from 17.2% to 41.0%, respectively.^9–13,27,28^ Although cross-trial comparisons should be interpreted cautiously due to differences in patient populations, sample sizes, and trial designs, our perioperative penpulimab plus neoadjuvant chemotherapy regimen yielded comparable MPR (57.7%) and pCR (50.0%) rates to those observed with other perioperative ICIs.^9–13,27,28^ Notably, the chemotherapy-free regimen of penpulimab plus anlotinib also demonstrated promising efficacy, with MPR and pCR rates of 52.4% and 38.1%, respectively, which aligned with previously reported outcomes.^9–13,27,28^ Furthermore, the combination of anlotinib plus penpulimab and chemotherapy further increased the MPR rate to 76.0%, while maintaining a comparable pCR rate of 52.0% to that of the other regimens. The promising efficacy observed with penpulimab plus anlotinib with or without chemotherapy may be attributed to the role of anlotinib in modulating the tumor microenvironment, conferring synergistic antitumor effects when combined with immunotherapy and/or chemotherapy. Specifically, anlotinib can convert the intrinsically immunosuppressive tumor microenvironment to an immunosupportive microenvironment by normalizing tumor vessels and increasing the infiltration of immune effector cells into tumors.^29,30^ Additionally, preclinical studies have shown that anlotinib reduces the stiffness of the ECM by modulating the expression of ECM-related genes and proteins, thereby increasing the retention time and distribution of ICIs or chemotherapeutic agents at the tumor site.^22,31,32^ Moreover, anlotinib induced notable loosening of the tumor tissue structure, which was associated with decreased interstitial fluid pressure and solid stress, potentially facilitating the penetration of antitumor drugs.^22,33^ These findings suggest the therapeutic potential of combining perioperative ICIs and antiangiogenic agents with or without neoadjuvant chemotherapy for patients with resectable NSCLC.

One interesting finding of this study was that adding anlotinib to penpulimab plus chemotherapy notably increased the MPR rate (76.0% vs 57.7%) while having a modest effect on the pCR rate (52.0% vs 50.0%). Similarly, anlotinib versus chemotherapy in combination with penpulimab had a comparable MPR rate (57.7% vs 52.4%) but a numerically lower pCR rate (50.0% vs 38.1%). This discrepancy indicated that achieving pCR may depend more critically on the direct cytotoxic effects of chemotherapy, which induces tumor cell apoptosis and other forms of nonapoptotic death, thereby eradicating cancer cells at the histopathological level.^34,35^ In contrast, anlotinib primarily contributes to microenvironment modulation and vascular normalization, facilitating partial tumor clearance but not necessarily completing pathological eradication.^29,30^ Notably, the 12- and 24-month EFS results revealed an intriguing pattern in which the penpulimab plus chemotherapy group presented favorable outcomes at 12 months but experienced a pronounced decline by 24 months, whereas the penpulimab plus anlotinib group appeared to maintain more durable long-term outcomes (supplementary Fig. 1a). This observation may reflect underlying mechanistic differences, with chemotherapy primarily delivering short-term cytotoxic effects, whereas anlotinib potentially promoted more durable immune modulation by remodeling the tumor microenvironment.^29,30,36,37^ Given the limited sample size, these findings should be interpreted with caution and warrant further validation in future studies.

Clinically meaningful improvements in MPR with penpulimab plus anlotinib and chemotherapy over penpulimab plus chemotherapy or penpulimab plus anlotinib were also observed across key subgroups. Although patients aged ≥65 years, female patients, current smokers, those with N0 stage disease, or those with tumor PD-L1 expression levels of <1% or ≥1% appeared to derive relatively less benefit than penpulimab plus chemotherapy or penpulimab plus anlotinib, these subgroups were relatively small and had low event rates, resulting in wide and overlapping CIs. In the present study, over 75% of patients across the three groups had squamous NSCLC, a proportion comparable to that reported in the Chinese population of the Neotorch and RATIONALE-315 trials (77–79%) but notably higher than that reported in the global KEYNOTE-671, AEGEAN, and CheckMate 77 T trials (43–51%).^8,10–13^ This discrepancy may be partly attributed to the exclusion of patients with known epidermal growth factor receptor (*EGFR*) mutations, which are more prevalent in Asian populations and significantly correlated with adenocarcinoma histology.^38,39^ Among our patients receiving perioperative penpulimab plus chemotherapy, the overall MPR rate was consistent with that reported in Chinese cohorts (48.5%-56%) and higher than that reported in global cohorts (30.2–35.4%).^8,10–13^ These findings aligned with prior trials of perioperative chemoimmunotherapy, which demonstrated that patients with squamous histology generally achieved a higher MPR rate than those with nonsquamous histology did, highlighting the influence of tumor histology on perioperative chemoimmunotherapy efficacy.^11,12^ Notably, penpulimab plus anlotinib and chemotherapy demonstrated particularly pronounced MPR benefits over penpulimab plus chemotherapy in patients with squamous NSCLC (79.0% vs 55.0%) and stage IIIA/IIIB disease (82.4% vs 55.6%), suggesting potential enhanced efficacy in these subgroups. However, as the subgroup analyses were descriptive and the study was not powered to detect differences among subgroups, these findings should be interpreted with caution.

The safety profiles of perioperative penpulimab plus anlotinib with or without neoadjuvant chemotherapy were consistent with those of previous reports, with no new safety signals identified.^25,26^ The incidences of grade ≥3 TRAEs (26.7% vs 20.0% vs 30.0%) and serious AEs (26.7% *vs* 30.0% vs 30.0%) were comparable across the three treatment groups, aligning with prior findings for perioperative ICI plus neoadjuvant chemotherapy (grade ≥3 TRAEs, 32–72%; serious AEs, 15–38%).^8,11,13^ As expected, irAEs were more common in the penpulimab plus anlotinib and chemotherapy groups than in the penpulimab plus chemotherapy or penpulimab plus anlotinib groups (36.7% vs 20.0% vs 23.3%); however, most events were grade 1–2. Notably, the incidence of irAEs associated with penpulimab plus anlotinib and chemotherapy was within the reported range for prior perioperative ICI plus neoadjuvant chemotherapy (16–40%), penpulimab monotherapy or combination therapy (24–48.5%).^8,11,13,26,40^ Importantly, the addition of anlotinib to penpulimab plus chemotherapy did not lead to more treatment discontinuation (3.3% vs. 6.7% vs. 13.3%) than penpulimab plus chemotherapy or penpulimab plus anlotinib did. No treatment-related deaths were observed across the three groups. Overall, perioperative penpulimab plus anlotinib combined with neoadjuvant chemotherapy demonstrated an acceptable and manageable safety profile in patients with resectable NSCLC.

The study had several limitations. First, the open-label design and the absence of a blinded central assessment may have introduced bias in evaluations of surgical resectability, radiographic responses, and pathological endpoints. Future phase III trials should incorporate blinded independent radiologic and pathology reviews to improve the reliability of efficacy assessments. Second, although the trial achieved its planned enrollment of 90 patients, the effective sample size of approximately 30 patients per group resulted in limited statistical power and wide CIs around key efficacy estimates. This increased the risk of type II error and warranted cautious interpretation of treatment comparisons. Third, no stratification factors (e.g., pathology, clinical stage, or PD-L1 expression) were applied during randomization, which may have led to baseline imbalances and potential confounding factors. Fourth, the underrepresentation of the Chinese population limits the generalizability of our results to the broader resectable NSCLC patient population. Fifth, the follow-up duration was relatively short, and the median EFS and OS were not reached at the time of analysis. Consequently, conclusions regarding survival benefits should be interpreted with caution, and longer follow-up with updated analyses would be necessary to determine whether the observed early pathological and radiographic responses could translate into durable survival benefits. Sixth, although adding anlotinib to penpulimab plus chemotherapy appeared to improve the MPR, its limited effect on pCR remains exploratory. The proposed mechanism explanation was hypothetical, as no biomarker or mechanistic analyses were conducted to support these observations, warranting further investigation in future trials. Finally, the trial design did not allow for a direct evaluation of the separate contributions of neoadjuvant and adjuvant components across different treatment regimens. To address this issue comprehensively, a phase III trial with multiple comparative cohorts and a larger sample size is necessary.

In conclusion, perioperative penpulimab plus anlotinib with or without neoadjuvant chemotherapy demonstrated promising efficacy and manageable safety profiles in patients with resectable NSCLC. Although a numerically higher MPR rate was observed with perioperative penpulimab plus anlotinib combined with neoadjuvant chemotherapy, this finding should be interpreted cautiously given the exploratory nature of this trial. Further data from ongoing and future clinical trials are needed to confirm the clinical benefits of combining perioperative ICI and an anti-angiogenic agent with or without neoadjuvant chemotherapy in this patient population.

## Materials and methods

### Ethics

This study was registered with ClinicalTrials.gov (NCT04846634) and was conducted in accordance with the Declaration of Helsinki and Good Clinical Practices. The protocol was approved by the ethics committee of each participating center (center ethics approval number from the Tianjin Medical University Cancer Institute and Hospital was E20221162). Written informed consent was obtained from each patient before enrollment.

### Study design and patients

ALTER-L043 was an open-label, multicenter, randomized, phase II study conducted in 6 medical centers across China. Patients were eligible if they were aged 18–70 years and had histopathology or cytologically confirmed treatment-naïve resectable stage IIB–IIIB (N2) NSCLC according to the eighth edition of the American Joint Committee Cancer Staging Manual.^41^ Additional eligibility criteria were an Eastern Cooperative Oncology Group (ECOG) performance status of 0–1, a life expectancy of ≥12 weeks, at least one measurable target lesion per Response Evaluation Criteria in Solid Tumors version 1.1 (RECIST 1.1), and adequate bone marrow and organ function. Patients with known *EGFR* mutations or anaplastic lymphoma kinase (*ALK*)*/*c-ros oncogene 1 (*ROS1*) fusions, a history of other malignancies within the last 5 years, known allergies or hypersensitivity reactions to any of the study treatments, or any severe or uncontrolled intercurrent illness were excluded. The complete eligibility criteria are listed in the Supplementary Appendix and study protocol.

### Randomization and treatments

**Fig. 3:**
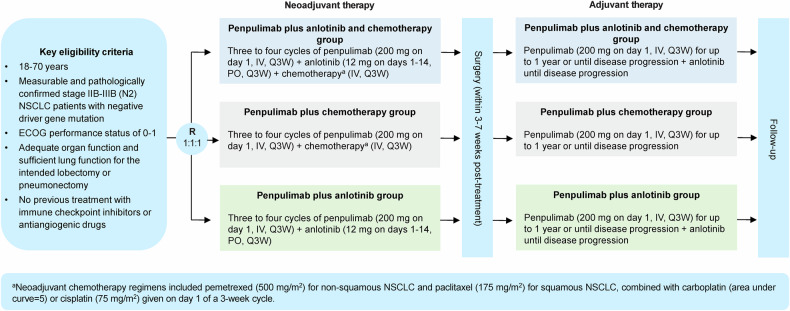
Study design of the ALTER-L043 study. NSCLC non-small cell lung cancer, ECOG Eastern Cooperative Oncology Group, R randomization, IV intravenous, Q3W every 3 weeks

Randomization was performed via the random envelope method, and each eligible patient was assigned a random number that was placed in the random envelope. Patients were randomly assigned at a 1:1:1 ratio to receive 3‒4 cycles of neoadjuvant penpulimab (200 mg intravenously on day 1 every 3 weeks) plus anlotinib (12 mg orally once daily on days 1‒14 every 3 weeks) and chemotherapy, penpulimab plus chemotherapy, or penpulimab plus anlotinib, followed by surgery and adjuvant penpulimab with or without anlotinib (Fig. 3). Neoadjuvant chemotherapy regimens included intravenous pemetrexed (500 mg/m^2^) for nonsquamous NSCLC and paclitaxel (175 mg/m^2^) for squamous NSCLC, combined with carboplatin (area under curve=5) or cisplatin (75 mg/m^2^) given on day 1 of a 3-week cycle. Surgical resection was performed within 3–7 weeks of the last dose of neoadjuvant therapy, with adjuvant therapy starting 4–6 weeks post-surgery. Adjuvant anlotinib continued until disease progression, while penpulimab was administered for up to 1 year or until disease progression.

Dose reduction of penpulimab was not permitted, but penpulimab treatment could be interrupted in cases of grade 2 or 3 toxicities (excluding grade 3 liver enzyme abnormalities, pneumonitis, and neurotoxicities) or discontinued upon grade 4 toxicities and grade 3 liver enzyme abnormalities, pneumonitis, and neurotoxicities. The maximum interval allowed for penpulimab dose suspension was 12 weeks. For grade ≥3 irAEs, systemic glucocorticoid (e.g., prednisone or intravenous equivalent) therapy was considered, with detailed information provided in the trial protocol (pp 74–86). Dose modifications of anlotinib from 12 mg to 10 mg or 8 mg were allowed to manage unacceptable toxicities. If patients continued to experience intolerable toxicity after the anlotinib dose was reduced to 8 mg, treatment was discontinued. In-study crossover was not permitted.

### Outcomes and assessments

The primary endpoint was the investigator-assessed MPR rate, which was defined as the proportion of patients with 10% or less residual viable tumor cells in the resected primary tumor. The secondary endpoints included investigator-assessed pCR (absence of residual viable tumor cells in the resected primary tumor and lymph nodes), objective response rate (ORR; the proportion of patients with the best overall response [CR] or partial response [PR] prior to surgery), EFS (time from treatment initiation to the first occurrence of local progression precluding planned surgery, unresectable tumor, local or distant recurrence, or death from any cause, whichever occurred first), 12-month EFS rate (the proportion of patients without local progression precluding planned surgery, unresectable tumor, local or distant recurrence, or death within 1 year from treatment initiation), OS (time from enrollment to death from any cause), and safety.

Tumor assessments with positron emission tomography-computed tomography (PET-CT) or CT were performed by investigators per RECIST 1.1 at baseline, at the end of cycle 2 of neoadjuvant therapy, prior to surgery, every 3 months during the first year after surgery, and then every 6 months during the 2–5 years after surgery. AEs were continuously monitored until 21 days after the last dose of the study treatment and were graded according to the Common Terminology Criteria for Adverse Events version 5.0 (CTCAE 5.0).

### Statistical analysis

The sample size was determined according to the primary endpoint of the MPR rate via PASS software version 15.0 (NCSS, Kaysville, UT, USA). On the basis of the historical MPR rate of 20.0% for neoadjuvant atezolizumab monotherapy in patients with resectable NSCLC, we hypothesized that the combination of perioperative penpulimab plus anlotinib with or without neoadjuvant chemotherapy would increase the MPR rate to 42.0%.^42^ With this assumption, a sample of 90 patients (30 per group) was needed to obtain a power of 80.0% and a two-sided alpha of 0.05, with a dropout rate of 10.0%.

Efficacy analyses were performed in the FAS, which consisted of patients who received at least one dose of the study drug and underwent a posttreatment tumor evaluation. Safety analyses were conducted in the SS, which included patients who received at least one dose of the study drug and had at least one safety assessment. The paired t test or Wilcoxon signed-rank test was applied to compare the baseline variables between treatment arms. The MPR rate, pCR rate, and ORR were summarized descriptively, with corresponding 95% CIs calculated via the Clopper‒Pearson method. Between-group comparisons of MPR and pCR rates were assessed via the chi-square test or Fisher’s exact test. The time-to-event endpoints (EFS and OS) were estimated via the Kaplan‒Meier method. The Brookmeyer‒Crowley method and Greenwood formula were used to calculate the 95% CIs for the medians and rates of time‒to-event endpoints, respectively. Safety data are presented as frequencies and percentages. Statistical significance was set at a two-sided *p* < 0.05, and SAS Software (version 9.4, SAS Institute Inc., Cary, NC, USA) was used for statistical analyses.

## Supplementary information

Sigtrans_Supplementary_Materials

Study Protocol

SAP

CONSORT_2025_editable_checklist

## Data Availability

The datasets supporting the findings of the current study are included in the manuscript and its Supplemental Materials. The datasets are available from the corresponding author upon reasonable request.
