# Supplementary material for: Perioperative penpulimab-based combination therapy in patients with resectable non-small cell lung cancer (ALTER-L043): an open-label, multicenter, randomized, phase II trial
Source: Signal Transduct Target Ther. 2026 Jan 16;11:21. doi: 10.1038/s41392-025-02544-w (PMC12811312; doi:10.1038/s41392-025-02544-w)
Supplement: Supplementary file 1 — Sigtrans_Supplementary_Materials [file 41392_2025_2544_MOESM1_ESM.docx]

Supplementary Materials for

Perioperative penpulimab-based combination therapy in patients with resectable non-small cell lung cancer (ALTER-L043): an open-label, multicenter, randomized, phase II trial

Meng Wang^#,*^, Weiran Liu^#^, Hongbo Guo, Hao Long, Bentong Yu, Guofang Zhao, Jun Wu, Dongsheng Yue, Xiaoliang Zhao, Chenguang Li, Lianmin Zhang, Shengguang Wang, Qiang Zhang, Zhenfa Zhang^*^, Changli Wang^*^

^#^ Meng Wang and Weiran Liu contributed equally as first authors.

^*^Correspondence to: wangchangli@tjmuch.com; wangmeng312@126.com; zhangzhenfa1973@163.com.

**This PDF file includes:**

Supplementary materials and methods

Tables S1 to S7

Figure S1

**Other Supplementary Materials for this manuscript include the following:**

Study protocol

Statistical analysis plan

**Supplementary materials and methods**

The inclusion criteria were:

1. Patients who were willing to enroll in the study and provide written informed consent, have good compliance, and cooperate with follow-up.
2. Patients who aged 18-70 years;
3. Patients who had an Eastern Cooperative Oncology Group (ECOG) performance status of 0-1;
4. Patients who had a life expectancy of at least 12 weeks;
5. Patients with childbearing potential must agree to use reliable contraception before enrollment, throughout the study, and up to 8 weeks after the last dose of the study drug;
6. Patients who agreed to provide tumor tissue specimens for this study;
7. Patients who agreed to receive radical surgery;
8. Patients without surgical contraindications, as assessed by specialist physicians;
9. Patients who had histopathology or cytologically confirmed non-small cell lung cancer (NSCLC) according to 2015 World Health Organization classification; resectable stage IIB-IIIB (N2) NSCLC according to the eighth edition of the American Joint Committee Cancer Staging Manual; without epidermal growth factor receptor (*EGFR*) mutations or anaplastic lymphoma kinase (*ALK*)/c-ros oncogene 1 (*ROS1*) fusions in primary tumor and lymph nodes (the need for genetic testing in patients with squamous NSCLC was determined by the investigator);
10. Patients with treatment-naïve NSCLC who had never received surgery, chemotherapy, radiotherapy or biotherapy;
11. Patients who could provide adequate tumor tissue specimens (non-cytological) for molecular marker analysis;
12. Patients who had at least one measurable lesion per Response Evaluation Criteria in Solid Tumors version 1.1 (RECIST 1.1);
13. Patients who had adequate lung function tolerating the proposed lung surgery;
14. Patients with adequate lung ventilation function (forced expiratory volume in 1 s [FEV1]≥1 L or FEV1≥800 mL after lobectomy/pneumonectomy), hematologic function (hemoglobin≥90g/L, absolute neutrophil count≥1.5×10^9^/L, and platelet count≥100×10^9^/L), hepatic function (aspartate aminotransferase [AST] and alanine aminotransferase [ALT]≤2.5×upper normal limit [ULN] and total bilirubin ≤1.5×ULN), and renal function (serum creatinine≤1.5×ULN or creatinine clearance≥60 mL/min);
15. Coagulation function must meet: activated partial thromboplastin time (APTT) or PTT≤1.5×ULN;
16. Females (15-49 years) or males with childbearing potential must agree to use reliable contraception (such as intrauterine devices, birth control pills, or condoms) during the study and up to 6 months after it was completed; For females with childbearing potential, a negative serum and urine pregnancy test was confirmed within 7 days before enrollment; Females who were not breastfeeding;

The exclusion criteria were:

1. Patients with large cell carcinoma or mixed small cell/large cell carcinoma;
2. Patients who had a high risk of lethal hemorrhage due to tumor invasion of critical blood vessels during the study, as determined by the investigator; or those with obvious lung cavitary or necrotic tumor.
3. Patients who had previous systemic anti-cancer treatment for NSCLC, including cytotoxic drug therapy, immunotherapy, or experimental treatment;
4. Patients who had previous local radiotherapy;
5. Patients who had a history of another malignant disease other than cervical carcinoma in situ, cured basal cell carcinoma, or epithelial tumors of the bladder (stages Ta and Tis) within the last 5 years;
6. Patients who had previously received anlotinib or other anti-angiogenic targeted drugs;
7. Patients who had previously received penpulimab, other anti-programmed cell death-1/ligand 1 (PD-1/PD-L1) or anti-cytotoxic T lymphocyte-associated antigen-4 (CTLA-4) antibodies, or any other therapies targeting T-cell co-stimulation or checkpoint pathways, such as anti-inducible co-stimulator (ICOS) agonists antibodies (e.g., CD40, CD137, GITR, OX40, et al.);
8. Patients with known allergy or hypersensitivity reaction to any of the study treatments;
9. Patients who had multiple factors affecting oral medication (such as inability to swallow, chronic diarrhea, and intestinal obstruction);
10. Patients with any severe and/or uncontrolled disease:

- Poor blood pressure control (systolic blood pressure≥150 mmHg or diastolic blood pressure≥100 mmHg);
- Class Ⅰ or more of myocardial ischemia or myocardial infarction, arrhythmia (including subjects with corrected QT interval [QTc]≥480 ms and congestive heart failure in New York Heart Association [NYHA] functional class≥II);
- Abnormal coagulation function (international normalized ratio [INR]>1.5 or prothrombin time [PT]>ULN+4 s or APTT>1.5×ULN) with a bleeding tendency, or were receiving thrombolytic or anticoagulant therapy; Note: On the premise that the INR of PT≤1.5, the use of low-dose heparin (daily dosage of 6000-12,000 U for adults) or low-dose aspirin (daily dosage of≤100 mg) was allowed for prophylactic purposes);
- Active or uncontrolled severe infection;
- Liver cirrhosis, decompensated liver disease, active hepatitis, or chronic hepatitis that need to receive antiviral therapy;
- Kidney failure needs haemodialysis and peritoneal dialysis;
- History of immune deficiency disorders including HIV-positive or other acquired or congenital immunodeficiency diseases or a history of organ transplantation;
- Poor blood glucose control (fasting blood glucose [FBG]>10 mmol/L);
- Urinalysis showed urine protein≥+ +, and confirmed 24-hour urine protein>1.0 g;
- Seizures that needed treatment;
- Patients with long-standing, unhealed wounds or fractures;
- Patients who had experienced clinically significant hemoptysis (over 50 mL per day) within 2 weeks prior to enrollment; or those with significant bleeding symptoms or a clear predisposition to bleeding, such as gastrointestinal bleeding, hemorrhagic gastric ulcers, fecal occult blood test results of ++ or higher at baseline, or those with vasculitis;

1. History of interstitial lung disease, drug-induced interstitial lung disease, radiation pneumonitis that required steroid hormone therapy, or any active interstitial lung disease with clinical manifestations;
2. Patients with excessive arterial/venous thrombosis events before enrollment or within 6 months, such as cerebrovascular accidents (including transient ischemic attacks), deep vein thrombosis, and pulmonary embolism;
3. Patients with grade≥2 peripheral neuropathy per the Common Terminology Criteria for Adverse Events, excluding cases resulting from wounds;
4. Patients requiring a right pneumonectomy; or patients who had undergone major surgery or had severe trauma, with the effects not fully resolved within 14 days prior to enrollment;
5. Patients currently participating in another clinical trial or who had completed their previous clinical trial within 4 weeks prior to enrollment;
6. Patients with small cell lung cancer;
7. Patients who received live or attenuated vaccines within 30 days before the first dose of penpulimab, or who intend to receive these vaccines during the study period;
8. Severe hypersensitivity to other monoclonal antibodies;
9. Women who were pregnant or breastfeeding;
10. Concomitant diseases seriously endanger patients’ safety or interfere with the completion of the study in the opinion of investigators.

**Supplementary tables**

## Table S1. Reasons for surgery cancellation

| Patient No. | Treatment group | Reasons for surgery cancellation |
| --- | --- | --- |
| 1 | Penpulimab plus anlotinib and chemotherapy | This patient had a stable disease (SD) after three cycles of neoadjuvant therapy and was deemed ineligible for surgery by the investigator due to an increased risk of perioperative pneumonia. |
| 2 | Penpulimab plus anlotinib and chemotherapy | This patient achieved a partial response (PR) after three cycles of neoadjuvant therapy, and the only adverse event (AE) was grade 4 febrile myelosuppression (considered unrelated to the study drugs by the investigator), but the patient declined surgery. |
| 3 | Penpulimab plus chemotherapy | This patient had SD after four cycles of neoadjuvant therapy with AEs including grade 2 anemia and grade 3 leukopenia (both considered possibly related to penpulimab by the investigator) and declined surgery. |
| 4 | Penpulimab plus chemotherapy | This patient achieved PR after four cycles of neoadjuvant therapy with only grade 1 rash (considered possibly related to penpulimab by the investigator) and also refused surgery. |
| 5 | Penpulimab plus anlotinib | This patient achieved SD after four cycles of neoadjuvant therapy with AEs including grade 1 constipation and grade 1 fever (both considered unrelated to study drugs by the investigator). During the preoperative evaluation, the investigator recommended pneumonectomy based on the patient’s overall clinical status. However, due to recurrent constipation and fever, as well as concerns about undergoing pneumonectomy, the patient ultimately chose to withdraw from the study and receive other treatment. |
| 6 | Penpulimab plus anlotinib | This patient achieved SD after four cycles of neoadjuvant therapy with AEs including grade 1 oropharyngeal discomfort and grade 2 hypertension (both considered possibly related to study drugs by the investigator). During the preoperative evaluation, the investigator recommended pneumonectomy based on the patient’s overall clinical status. However, due to concerns regarding insufficiently controlled hypertension, as well as apprehension about undergoing pneumonectomy, the patient ultimately chose to withdraw from the study and receive other treatment. |
| 7 | Penpulimab plus anlotinib | This patient achieved SD after three cycles of neoadjuvant therapy with AEs including grade 1 ventricular arrhythmia and grade 2 hypertension (both considered possibly related to study drugs by the investigator). During the preoperative evaluation, the investigator deemed the patient clinically eligible for surgery. However, due to recurrent hypertension and concerns about potential exacerbation of AEs during postoperative adjuvant therapy, the patient ultimately declined surgery and withdrew from the study. |
| 8 | Penpulimab plus anlotinib | This patient achieved SD after three cycles of neoadjuvant therapy with AEs including grade 1 toothache, grade 1 leukopenia, grade 2 oral mucositis, and grade 3 hypertension (all considered possibly related to study drugs by the investigator). During the preoperative evaluation, the investigator deemed the patient clinically eligible for surgery. However, due to intolerance to toothache, oral mucositis, and hypertension, the patient ultimately declined surgery and withdrew from the study. |
| 9 | Penpulimab plus anlotinib | This patient with SD after three cycles of neoadjuvant therapy was deemed unsuitable for surgery due to poor pulmonary condition, potentially resulting in high surgical risk. |
| 10 | Penpulimab plus anlotinib | This patient with SD after four cycles of neoadjuvant therapy had grade 1 constipation and grade 1 fever (both considered unrelated to study drugs by the investigator) but was judged unsuitable for surgery owing to poor pulmonary condition, potentially resulting in high surgical risk. |
| 11 | Penpulimab plus anlotinib | This patient with PR after four cycles of neoadjuvant therapy was considered unsuitable for surgery due to severe obstructive ventilatory impairment and moderate diffusion dysfunction on pulmonary function testing. |
| 12 | Penpulimab plus anlotinib | This patient with PR after three cycles of neoadjuvant therapy refused surgery. |

## Table S2. Subgroup analyses for pCR in patients who underwent definitive surgery

|  | Penpulimab plus anlotinib and chemotherapy group (n=25) | |  | Penpulimab plus chemotherapy group (n=26) | |  | Penpulimab plus anlotinib group (n=21) | |  |
| --- | --- | --- | --- | --- | --- | --- | --- | --- | --- |
|  | n/N | pCR rate |  | n/N | pCR rate |  | n/N | pCR rate |  |
| Age, years | |  |  |  |  |  |  |  |  |
| <65 | | 8/15 | 53.3 (26.6-78.7) |  | 7/18 | 38.9 (17.3-64.3) |  | 5/12 | 41.7 (15.2-72.3) |
| ≥65 | | 5/10 | 50.0 (18.7-81.3) |  | 6/8 | 75.0 (34.9-96.8) |  | 3/9 | 33.3 (7.5-70.1) |
| Gender | |  |  |  |  |  |  |  |  |
| Male | | 12/23 | 52.2 (30.6-73.2) |  | 13/25 | 52.0 (31.3-72.2) |  | 7/20 | 35.0 (15.4-59.2) |
| Female | | 1/2 | 50.0 (1.3-98.7) |  | 0/1 | 0 |  | 1/1 | 100.0 (2.5-100.0) |
| Smoking status | |  |  |  |  |  |  |  |  |
| Never | | 0/3 | 0 |  | 0/3 | 0 |  | 1/4 | 25.0 (0.6-80.6) |
| Former | | 11/19 | 57.9 (33.5-79.8) |  | 13/18 | 72.2 (46.5-90.3) |  | 6/16 | 37.5 (15.2-64.6) |
| Current | | 2/3 | 66.7 (9.4-99.2) |  | 0/5 | 0 |  | 1/1 | 100.0 (2.5-100.0) |
| ECOG performance status | |  |  |  |  |  |  |  |  |
| 0 | | 8/14 | 57.1 (28.9-82.3) |  | 10/16 | 62.5 (35.4-84.8) |  | 5/16 | 31.3 (11.0-58.7) |
| 1 | | 5/11 | 45.5 (16.8-76.6) |  | 3/10 | 30.0 (6.7-65.3) |  | 3/5 | 60.0 (14.7-94.7) |
| Pathology | |  |  |  |  |  |  |  |  |
| Squamous carcinoma | | 11/19 | 57.9 (33.5-79.8) |  | 10/20 | 50.0 (27.2-72.8) |  | 6/18 | 33.3 (13.3-59.0) |
| Non-squamous | | 2/6 | 33.3 (4.3-77.7) |  | 3/6 | 50.0 (11.8-88.2) |  | 2/3 | 66.7 (9.4-99.2) |
| Clinical stage | |  |  |  |  |  |  |  |  |
| IIB | | 4/8 | 50.0 (15.7-84.3) |  | 9/17 | 52.9 (27.8-77.0) |  | 5/9 | 55.6 (21.2-86.3) |
| IIIA/IIIB | | 9/17 | 52.9 (27.8-77.0) |  | 4/9 | 44.4 (13.7-78.8) |  | 3/12 | 25.0 (5.5-57.2) |
| Node stage | |  |  |  |  |  |  |  |  |
| N0 | | 1/4 | 25.0 (0.6-80.6) |  | 5/6 | 83.3 (35.9-99.6) |  | 1/3 | 33.3 (0.8-90.6) |
| N1 | | 5/8 | 62.5 (24.5-91.5) |  | 4/11 | 36.4 (10.9-69.2) |  | 5/9 | 55.6 (21.2-86.3) |
| N2 | | 7/13 | 53.9 (25.1-80.8) |  | 4/9 | 44.4 (13.7-78.8) |  | 2/9 | 22.2 (2.8-60.0) |
| Tumor PD-L1 expression | |  |  |  |  |  |  |  |  |
| <1% | | 2/8 | 25.0 (3.2-65.1) |  | 2/5 | 40.0 (5.3-85.3) |  | 2/9 | 22.2 (2.8-60.0) |
| ≥1% | | 4/8 | 50.0 (15.7-84.3) |  | 7/12 | 58.3 (27.7-84.8) |  | 1/3 | 33.3 (0.8-90.6) |
| Unknown | | 7/9 | 77.8 (40.0-97.2) |  | 4/9 | 44.4 (13.7-78.8) |  | 5/9 | 55.6 (21.2-86.3) |

Data are n or % (95% CI). pCR, pathological complete response; ECOG, Eastern Cooperative Oncology Group; PD-L1, programmed cell death-ligand 1.

## Table S3. Subgroup analyses for 6-month and 12-month EFS in the full analysis set population

|  | Penpulimab plus anlotinib and chemotherapy group (n=27) | | |  | Penpulimab plus chemotherapy group (n=29) | | |  | Penpulimab plus anlotinib group (n=30) | | |
| --- | --- | --- | --- | --- | --- | --- | --- | --- | --- | --- | --- |
|  | n/N | 6-month EFS rate | 12-month EFS rate |  | n/N | 6-month EFS rate | 12-month EFS rate |  | n/N | 6-month EFS rate | 12-month EFS rate |
| Age, years |  |  |  |  |  |  |  |  |  |  |  |
| <65 | 1/17 | 100.0 (100.0-100.0) | 91.7 (53.9-98.8) |  | 3/18 | 100.0 (100.0-100.0) | 90.9 (50.8-98.7) |  | 4/17 | 79.4 (48.8-92.9) | 71.5 (40.4-88.3) |
| ≥65 | 0/10 | 100.0 (100.0-100.0) | 100.0 (100.0-100.0) |  | 1/11 | 85.7 (33.4-97.9) | 85.7 (33.4-97.9) |  | 2/13 | 80.2 (40.3-94.8) | 80.2 (40.3-94.8) |
| Gender |  |  |  |  |  |  |  |  |  |  |  |
| Male | 1/25 | 100.0 (100.0-100.0) | 93.3 (61.3-99.0) |  | 4/28 | 95.0 (69.5-99.3) | 88.7 (61.4-97.1) |  | 5/28 | 81.4 (57.4-92.7) | 75.6 (50.4-89.2) |
| Female | 0/2 | 100.0 (100.0-100.0) | NE |  | 0/1 | 100.0 (100.0-100.0) | 100.0 (100.0-100.0) |  | 1/2 | 50.0 (0.6-91.0) | NE |
| Smoking status |  |  |  |  |  |  |  |  |  |  |  |
| Never | 0/3 | 100.0 (100.0-100.0) | NE |  | 1/4 | 100.0 (100.0-100.0) | 50.0 (0.6-91.0) |  | 2/6 | 60.0 (12.6-88.2) | 60.0 (12.6-88.2) |
| Former | 1/20 | 100.0 (100.0-100.0) | 93.3 (61.3-99.0) |  | 3/20 | 93.8 (63.2-99.1) | 93.8 (63.2-99.1) |  | 4/22 | 83.0 (55.8-94.3) | 76.6 (48.7-90.6) |
| Current | 0/4 | 100.0 (100.0-100.0) | NE |  | 0/5 | 100.0 (100.0-100.0) | 100.0 (100.0-100.0) |  | 0/2 | NE | NE |
| ECOG performance status |  |  |  |  |  |  |  |  |  |  |  |
| 0 | 1/14 | 100.0 (100.0-100.0) | 90.0 (47.3-98.5) |  | 2/17 | 92.3 (56.6-98.9) | 92.3 (56.6-98.9) |  | 4/19 | 82.0 (53.5-93.9) | 75.2 (46.1-90.0) |
| 1 | 0/13 | 100.0 (100.0-100.0) | 100.0 (100.0-100.0) |  | 2/12 | 100.0 (100.0-100.0) | 83.3 (27.3-97.5) |  | 2/11 | 71.4 (25.8-92.0) | 71.4 (25.8-92.0) |
| Pathology |  |  |  |  |  |  |  |  |  |  |  |
| Squamous carcinoma | 1/21 | 100.0 (100.0-100.0) | 92.9 (59.1-99.0) |  | 3/22 | 93.3 (61.3-99.0) | 93.3 (61.3-99.0) |  | 5/26 | 79.9 (54.6-92.0) | 74.2 (48.3-88.5) |
| Non-squamous | 0/6 | 100.0 (100.0-100.0) | 100.0 (100.0-100.0) |  | 1/7 | 100.0 (100.0-100.0) | 80.0 (20.4-96.9) |  | 1/4 | 75.0 (12.8-96.1) | 75.0 (12.8-96.1) |
| Clinical stage |  |  |  |  |  |  |  |  |  |  |  |
| IIB | 0/10 | 100.0 (100.0-100.0) | 100.0 (100.0-100.0) |  | 3/19 | 92.3 (56.6-98.9) | 83.9 (49.4-95.7) |  | 1/12 | 87.5 (38.7-98.1) | 87.5 (38.7-98.1) |
| IIIA/IIIB | 1/17 | 100.0 (100.0-100.0) | 90.0 (47.3-98.5) |  | 1/10 | 100.0 (100.0-100.0) | 100.0 (100.0-100.0) |  | 5/18 | 72.1 (41.5-88.6) | 63.1 (32.1-83.0) |
| Node stage |  |  |  |  |  |  |  |  |  |  |  |
| N0 | 0/5 | 100.0 (100.0-100.0) | 100.0 (100.0-100.0) |  | 1/8 | 83.3 (27.3-97.5) | 83.3 (27.3-97.5) |  | 1/4 | 75.0 (12.8-96.1) | 75.0 (12.8-96.1) |
| N1 | 0/9 | 100.0 (100.0-100.0) | 100.0 (100.0-100.0) |  | 2/11 | 100.0 (100.0-100.0) | 83.3 (27.3-97.5) |  | 2/13 | 80.8 (42.4-94.9) | 80.8 (42.4-94.9) |
| N2 | 1/13 | 100.0 (100.0-100.0) | 88.9 (43.3-98.4) |  | 1/10 | 100.0 (100.0-100.0) | 100.0 (100.0-100.0) |  | 3/13 | 77.8 (36.5-93.9) | 64.8 (25.3-87.2) |
| Tumor PD-L1 expression |  |  |  |  |  |  |  |  |  |  |  |
| <1% | 0/8 | 100.0 (100.0-100.0) | 100.0 (100.0-100.0) |  | 2/6 | 75.0 (12.8-96.1) | 75.0 (12.8-96.1) |  | 2/10 | 87.5 (38.7-98.1) | 70.0 (22.5-91.8) |
| ≥1% | 1/9 | 100.0 (100.0-100.0) | 83.3 (27.3-97.5) |  | 0/13 | 100.0 (100.0-100.0) | 100.0 (100.0-100.0) |  | 0/3 | 100.0 (100.0-100.0) | 100.0 (100.0-100.0) |
| Unknown | 0/10 | 100.0 (100.0-100.0) | 100.0 (100.0-100.0) |  | 2/10 | 100.0 (100.0-100.0) | 83.3 (27.3-97.5) |  | 4/17 | 68.8 (36.4-87.1) | 68.8 (36.4-87.1) |

Data are n or % (95% CI). EFS, event-free survival; ECOG, Eastern Cooperative Oncology Group; PD-L1, programmed cell death-ligand 1; NE, not evaluable.

## Table S4. Summary of adverse events in the safety analysis set population

|  | Penpulimab plus anlotinib and chemotherapy group (n=30) | Penpulimab plus chemotherapy group (n=30) | Penpulimab plus anlotinib group (n=30) |
| --- | --- | --- | --- |
| Treatment-emergent adverse events |  |  |  |
| Any grade | 26 (86.7) | 29 (96.7) | 28 (93.3) |
| Grade ≥3 | 15 (50.0) | 12 (40.0) | 12 (40.0) |
| Leading to any dose reduction or interruption | 12 (40.0) | 4 (13.3) | 8 (26.7) |
| Leading to any treatment discontinuation | 3 (10.0) | 3 (10.0) | 6 (20.0) |
| Leading to death | 0 | 0 | 1 (3.3) |
| Treatment-related adverse events |  |  |  |
| Any grade | 22 (73.3) | 18 (60.0) | 23 (76.7) |
| Grade ≥3 | 8 (26.7) | 6 (20.0) | 9 (30.0) |
| Leading to any dose reduction or interruption | 9 (30.0) | 3 (10.0) | 8 (26.7) |
| Leading to any treatment discontinuation | 1 (3.3) | 2 (6.7) | 4 (13.3) |
| Leading to death | 0 | 0 | 0 |

Data are n (%).

## Table S5. Treatment-emergent adverse events occurring in ≥10% of patients in the safety analysis set population

|  | Penpulimab plus anlotinib and chemotherapy group (n=30) | | |  | Penpulimab plus chemotherapy group (n=30) | | |  | Penpulimab plus anlotinib group (n=30) | | |
| --- | --- | --- | --- | --- | --- | --- | --- | --- | --- | --- | --- |
|  | Any grade | Grade 1-2 | Grade 3-5 |  | Any grade | Grade 1-2 | Grade 3-5 |  | Any grade | Grade 1-2 | Grade 3-5 |
| Nausea | 12 (40.0) | 10 (33.3) | 2 (6.7) |  | 3 (10.0) | 2 (6.7) | 1 (3.3) |  | 0 | 0 | 0 |
| Productive cough | 10 (33.3) | 10 (33.3) | 0 |  | 8 (26.7) | 8 (26.7) | 0 |  | 3 (10.0) | 3 (10.0) | 0 |
| Postoperative wound complications | 9 (30.0) | 9 (30.0) | 0 |  | 16 (53.3) | 16 (53.3) | 0 |  | 17 (56.7) | 17 (56.7) | 0 |
| Cough | 7 (23.3) | 7 (23.3) | 0 |  | 6 (20.0) | 6 (20.0) | 0 |  | 5 (16.7) | 5 (16.7) | 0 |
| Weakness | 7 (23.3) | 7 (23.3) | 0 |  | 4 (13.3) | 4 (13.3) | 0 |  | 5 (16.7) | 5 (16.7) | 0 |
| Anorexia | 7 (23.3) | 5 (16.7) | 2 (6.7) |  | 3 (10.0) | 3 (10.0) | 0 |  | 1 (3.3) | 0 | 1 (3.3) |
| Elevated ALT | 7 (23.3) | 7 (23.3) | 0 |  | 2 (6.7) | 2 (6.7) | 0 |  | 0 | 0 | 0 |
| Pneumothorax | 7 (23.3) | 6 (20.0) | 1 (3.3) |  | 2 (6.7) | 2 (6.7) | 0 |  | 0 | 0 | 0 |
| Hypertension | 7 (23.3) | 4 (13.3) | 3 (10.0) |  | 1 (3.3) | 0 | 1 (3.3) |  | 7 (23.3) | 3 (10.0) | 4 (13.3) |
| Pleural effusion | 6 (20.0) | 5 (16.7) | 1 (3.3) |  | 2 (6.7) | 1 (3.3) | 1 (3.3) |  | 0 | 0 | 0 |
| Insomnia | 5 (16.7) | 5 (16.7) | 0 |  | 4 (13.3) | 4 (13.3) | 0 |  | 2 (6.7) | 2 (6.7) | 0 |
| Chest pain | 5 (16.7) | 5 (16.7) | 0 |  | 4 (13.3) | 4 (13.3) | 0 |  | 3 (10.0) | 3 (10.0) | 0 |
| Constipation | 5 (16.7) | 5 (16.7) | 0 |  | 3 (10.0) | 3 (10.0) | 0 |  | 3 (10.0) | 3 (10.0) | 0 |
| Hypoalbuminemia | 5 (16.7) | 5 (16.7) | 0 |  | 2 (6.7) | 2 (6.7) | 0 |  | 0 | 0 | 0 |
| Anemia | 5 (16.7) | 4 (13.3) | 1 (3.3) |  | 6 (20.0) | 5 (16.7) | 1 (3.3) |  | 0 | 0 | 0 |
| Thrombocytopenia | 5 (16.7) | 2 (6.7) | 3 (10.0) |  | 1 (3.3) | 1 (3.3) | 0 |  | 0 | 0 | 0 |
| Rash | 4 (13.3) | 4 (13.3) | 0 |  | 6 (20.0) | 5 (16.7) | 1 (3.3) |  | 8 (26.7) | 8 (26.7) | 0 |
| Oral ulceration | 4 (13.3) | 4 (13.3) | 0 |  | 1 (3.3) | 1 (3.3) | 0 |  | 4 (13.3) | 4 (13.3) | 0 |
| Elevated AST | 4 (13.3) | 4 (13.3) | 0 |  | 2 (6.7) | 2 (6.7) | 0 |  | 0 | 0 | 0 |
| Alopecia | 4 (13.3) | 4 (13.3) | 0 |  | 1 (3.3) | 1 (3.3) | 0 |  | 0 | 0 | 0 |
| Fever | 3 (10.0) | 3 (10.0) | 0 |  | 5 (16.7) | 5 (16.7) | 0 |  | 3 (10.0) | 2 (6.7) | 1 (3.3) |
| Toothache | 3 (10.0) | 3 (10.0) | 0 |  | 2 (6.7) | 2 (6.7) | 0 |  | 2 (6.7) | 2 (6.7) | 0 |
| Diarrhea | 3 (10.0) | 3 (10.0) | 0 |  | 1 (3.3) | 1 (3.3) | 0 |  | 2 (6.7) | 2 (6.7) | 0 |
| Arthralgia | 3 (10.0) | 2 (6.7) | 1 (3.3) |  | 0 | 0 | 0 |  | 3 (10.0) | 3 (10.0) | 0 |
| Abdominal distension | 3 (10.0) | 3 (10.0) | 0 |  | 0 | 0 | 0 |  | 0 | 0 | 0 |
| Oropharyngeal discomfort | 2 (6.7) | 2 (6.7) | 0 |  | 2 (6.7) | 1 (3.3) | 0 |  | 3 (10.0) | 3 (10.0) | 0 |
| Hypothyroidism | 2 (6.7) | 2 (6.7) | 0 |  | 1 (3.3) | 1 (3.3) | 0 |  | 3 (10.0) | 3 (10.0) | 0 |
| Leukopenia | 2 (6.7) | 2 (6.7) | 0 |  | 3 (10.0) | 1 (3.3) | 2 (6.7) |  | 1 (3.3) | 1 (3.3) | 0 |
| Infectious pneumonia | 1 (3.3) | 1 (3.3) | 0 |  | 3 (10.0) | 1 (3.3) | 2 (6.7) |  | 2 (6.7) | 0 | 2 (6.7) |
| Pain | 1 (3.3) | 1 (3.3) | 0 |  | 3 (10.0) | 3 (10.0) | 0 |  | 0 | 0 | 0 |
| Gastrointestinal distention | 1 (3.3) | 1 (3.3) | 0 |  | 3 (10.0) | 3 (10.0) | 0 |  | 0 | 0 | 0 |
| Hemoptysis | 1 (3.3) | 1 (3.3) | 0 |  | 0 | 0 | 0 |  | 3 (10.0) | 2 (6.7) | 1 (3.3) |

Data are n (%). ALT, alanine aminotransferase; AST, aspartate aminotransferase.

## Table S6. Serious adverse events in the safety analysis population

|  | Penpulimab plus anlotinib and chemotherapy group (n=30) | Penpulimab plus chemotherapy group (n=30) | Penpulimab plus anlotinib group (n=30) |
| --- | --- | --- | --- |
| Total | 8 (26.7) | 9 (30.0) | 9 (30.0) |
| Serious adverse events |  |  |  |
| Nausea | 2 (6.7) | 1 (3.3) | 0 |
| Myocardial infarction | 1 (3.3) | 0 | 0 |
| Pneumothorax | 1 (3.3) | 0 | 0 |
| Intestinal obstruction | 1 (3.3) | 0 | 0 |
| Pleural effusion | 1 (3.3) | 0 | 0 |
| Lacunar cerebral infarction | 1 (3.3) | 0 | 0 |
| Anorexia | 1 (3.3) | 0 | 1 (3.3) |
| Bone marrow suppression | 1 (3.3) | 0 | 0 |
| COVID-19 pneumonia | 0 | 0 | 1 (3.3) |
| Fever | 0 | 0 | 1 (3.3) |
| Febrile neutropenia | 0 | 1 (3.3) | 0 |
| Hemoptysis | 0 | 0 | 1 (3.3) |
| Cerebral infarction | 0 | 1 (3.3) | 2 (6.7) |
| Cerebral aneurysm | 0 | 0 | 1 (3.3) |
| Shingles | 0 | 1 (3.3) | 0 |
| Acute cholangitis | 0 | 0 | 1 (3.3) |
| Confusion | 0 | 0 | 1 (3.3) |
| Infectious pneumonia | 0 | 3 (10.0) | 2 (6.7) |
| Lymphocytic hypophysitis | 0 | 1 (3.3) | 0 |
| Rash | 0 | 1 (3.3) | 0 |
| Autoimmune hypothyroidism | 0 | 1 (3.3) | 0 |

Data are n (%). COVID-19, coronavirus disease 2019.

## Table S7. Immune-related adverse events in the safety analysis population

|  | Penpulimab plus anlotinib and chemotherapy group (n=30) | Penpulimab plus chemotherapy group (n=30) | Penpulimab plus anlotinib group (n=30) |
| --- | --- | --- | --- |
| Total | 11 (36.7) | 6 (20.0) | 7 (23.3) |
| Immune-related adverse events |  |  |  |
| Elevated ALT | 4 (13.3) | 1 (3.3) | 0 |
| Nausea | 3 (10.0) | 3 (10.0) | 0 |
| Anemia | 3 (10.0) | 2 (6.7) | 0 |
| Anorexia | 3 (10.0) | 0 | 0 |
| Arthralgia | 2 (6.7) | 0 | 1 (3.3) |
| Vomiting | 2 (6.7) | 0 | 0 |
| Toothache | 2 (6.7) | 2 (6.7) | 1 (3.3) |
| Hypothyroidism | 2 (6.7) | 0 | 3 (10.0) |
| Leukopenia | 2 (6.7) | 1 (3.3) | 1 (3.3) |
| Rash | 2 (6.7) | 2 (6.7) | 1 (3.3) |
| Neurological disorders | 2 (6.7) | 0 | 0 |
| Hypertension | 2 (6.7) | 0 | 3 (10.0) |
| Upper respiratory infection | 1 (3.3) | 1 (3.3) | 0 |
| Arthritis | 1 (3.3) | 0 | 0 |
| Oral ulceration | 1 (3.3) | 0 | 0 |
| Hemoptysis | 1 (3.3) | 0 | 1 (3.3) |
| Elevated AST | 1 (3.3) | 1 (3.3) | 0 |
| Myocardial infarction | 1 (3.3) | 0 | 0 |
| Myocardial ischemia | 1 (3.3) | 0 | 0 |
| Atrial fibrillation | 1 (3.3) | 0 | 0 |
| Pruritus | 1 (3.3) | 0 | 0 |
| Dermatitis | 1 (3.3) | 0 | 0 |
| Dizziness | 1 (3.3) | 0 | 0 |
| Conjunctival hemorrhage | 1 (3.3) | 0 | 0 |
| Myasthenia | 1 (3.3) | 0 | 0 |
| Gastrointestinal distention | 1 (3.3) | 1 (3.3) | 0 |
| Chest discomfort | 1 (3.3) | 0 | 0 |
| Alopecia | 1 (3.3) | 0 | 0 |
| Lacunar cerebral infarction | 1 (3.3) | 0 | 0 |
| Diarrhea | 1 (3.3) | 1 (3.3) | 0 |
| Abdominal distension | 1 (3.3) | 0 | 0 |
| Weakness | 1 (3.3) | 0 | 1 (3.3) |
| Thrombocytopenia | 1 (3.3) | 0 | 0 |
| Psoriasis | 1 (3.3) | 0 | 0 |
| Musculoskeletal pain | 1 (3.3) | 0 | 0 |
| Constipation | 0 | 1 (3.3) | 0 |
| Febrile neutropenia | 0 | 1 (3.3) | 0 |
| Oral mucositis | 0 | 0 | 1 (3.3) |
| Proteinuria | 0 | 0 | 1 (3.3) |
| Arrhythmia | 0 | 0 | 1 (3.3) |
| Lymphocytic hypophysitis | 0 | 1 (3.3) | 0 |
| Hyperthyroidism | 0 | 0 | 2 (6.7) |
| Skin pain | 0 | 1 (3.3) | 0 |
| Hypothyroidism | 0 | 1 (3.3) | 0 |

Data are n (%). ALT, alanine aminotransferase; AST, aspartate aminotransferase.

**Supplementary figure**


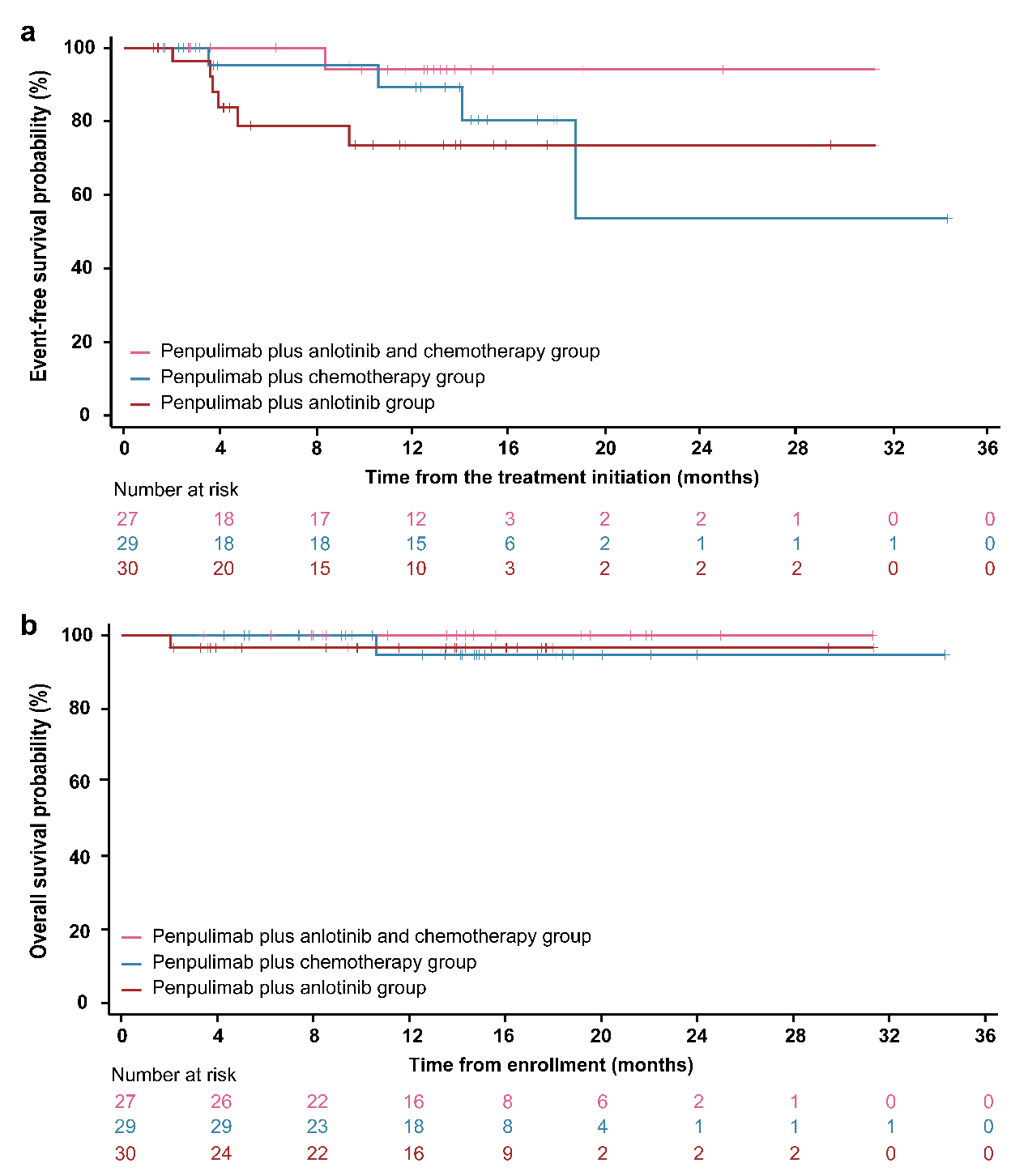


## Figure S1. Investigator-assessed event-free survival and overall survival in the full analysis set population

(a) Kaplan-Meier estimates of event-free survival in the three groups. (b) Kaplan-Meier estimates of overall survival in the three groups.
