# Supplementary material for: Perioperative penpulimab-based combination therapy in patients with resectable non-small cell lung cancer (ALTER-L043): an open-label, multicenter, randomized, phase II trial
Source: Signal Transduct Target Ther. 2026 Jan 16;11:21. doi: 10.1038/s41392-025-02544-w (PMC12811312; doi:10.1038/s41392-025-02544-w)
Supplement: Supplementary file 2 — Study Protocol [file 41392_2025_2544_MOESM2_ESM.pdf]

An Exploratory Phase II Clinical Study of a Penpulimab-Based Combination Regimen for Neoadjuvant/Adjuvant Treatment of Resectable Locally Advanced Non-Small Cell Lung Cancer

Study protocol

Principal Investigator: Prof. Changli Wang

Statistical unit: Tianjin Cancer Hospital

Co-organizer: Chia Tai Tianqing Pharmaceutical Group Co., Ltd.

Version No.: 3.0

Version Date: September 7, 2022

This document contains important confidential business information, which is proprietary to Chia Tai Tianqing Pharmaceutical Group Co., Ltd. and shall not be disclosed unless required by current laws or regulations, and in any case, this confidentiality requirement shall be communicated to all the persons who have received this material, and they may not disclose this information further. This

restriction of disclosure also applies to all documents that will be provided in the future and marked as confidential.

Program Signature Page

Signature of Investigator

I will conscientiously fulfill my duties as an investigator in accordance with Chinese GCP regulations by participating in or directly supervising this clinical study. We have read and acknowledge this protocol (Protocol No. ALTER-L043; Version No. 3.0; Version Date: September 7, 2022) and agree to its scientific and ethical validity. We will perform our duties in accordance with Chinese law, the Declaration of Helsinki, the Chinese GCP, and the protocol, and will modify the protocol only after notifying the sponsor, and will implement the protocol only with the consent of the Ethics Committee, except when measures are necessary to protect the safety, rights, and interests of the subjects.

We will keep this study protocol confidential.

Clinical research unit: Tianjin Cancer Hospital

Changli Wang		
Principal Investigator (Printed)	Principal Investigator (Signature)	Date of signature (year/month/day)

Program signature page

Signature of Statistical Unit

I have read and acknowledge this protocol (Protocol No. ALTER-L043; Version No. 3.0; Version Date: September 7, 2022) I agree to perform my duties in accordance with Chinese law, the Declaration of Helsinki, the Chinese GCP and this study protocol.

We will keep this study protocol confidential.

Tianjin Cancer Hospital

Principal person in charge (in print)	Principal person in charge (signature)	Date of signature (dd/mm/yyyy)

Signature page of the program

Signature of co-organizer

I have read and acknowledged this program (Program No. ALTER-L043; Version No. 3.0 ; Version Date: September 7, 2022) I agree to perform my duties in accordance with Chinese law, the Declaration of Helsinki, the Chinese GCP, and this study protocol.

We will keep this research program confidential.

Co-organizer: Chia Tai Tianqing Pharmaceutical Group Co.

Ma Leilei		
Principal (Printed)	Principal Officer (Signature)	Date of signature (dd/mm/yyyy)

Research Program Version Update Record

Version No.	Version Date
1.0	December 21, 2020
2.0	March 20, 2021
2.1	May 21, 2021
3.0	September 7, 2022

Note: Modifications are detailed in the Program Modifications and Approvals section.

Table of Contents

Program Summary 8
Clinical Study Flowchart 16
List of Abbreviations 21
1. Study Background 25
1.1 Study Feasibility 25
2The Study Drug 31
2.1 Anrotinib hydrochloride 31
2.2 Penpulimab 45
3. Purpose of the Study 49
3.1 Primary objective 49
3.2 Secondary objective 49
4. Trial Design 49
4.1 Overall design 49
4.2 Validity evaluation and analysis 53
4.3 Biomarker analysis 53
4.4 Survival follow-up 54
4.5 Adverse event follow-up 54
5. Subject selection and dropout 54
5.1 Inclusion Criteria 54
5.2 Exclusion Criteria 56
5.3 Exclusion Criteria 58
5.4 Subject termination criteria 59
5.5 Disposition of withdrawn subjects 59
6. Investigational Drugs 59
6.1 Overview of the investigational drug(s) 59
6.2 Dosage and dosing regimen 60
6.5 Symptomatic management of common adverse reactions and dose adjustments 63
6.6 Medication adherence 86
6.7 Drug administration, dispensing and recall 87
6.8 Concomitant medications 89
7. Study Steps 91
7.1 Screening visit 91
7.2 Treatment visits 95
7.3 Safety Follow-Up 98
7.4 Survival visits 98
7.5 Unplanned visits 99
8. Therapeutic Efficacy Evaluation Indicators 99
8.1 Main indicators and observation methods 99
9. Safety Evaluation 100
10. Data Management 107

10.1 Data collection 108
10.2 Data management and quality control 109
10.3 Review of data and monitoring of research organizations 109
10.4 Maintenance of test records 110
10.5 Data processing 111
11. Statistical analysis 111
11.1 Selection of data for statistical analysis 111
11.2 Principles and contents of statistical analysis 112
12. Ethics and informed consent 115
12.1 Laws and Regulations 115
12.2 Code of Ethics 115
12.3 Ethics Committee 115
12.4 Informed Consent 116
12.5 Confidentiality of Subject Information 116
13. Research Management 117
13.1 Research Management Organization 117
13.2 Standardized Practice 117
13.3 Training 117
13.4 Clinical monitoring 118
13.5 Recording and Retention of Study Data 119
13.6 Quality control and quality assurance 120
14. Publication of study results 120
15. Progress of clinical studies 121
16. Trial protocol modification and approval 121
17. Research Organization 121
17.1 Investigators 121
17.2 Co-organizers 122
Annex I Physical Condition Scoring Criteria (ECOG) 123
Annex II Classification of Cardiac Insufficiency 124
Annex III Calculation of creatinine clearance 125
Annex IV Eighth Edition Lung Cancer Staging Chart 126
References 130

Program Summary

Study Title	An Exploratory Phase II Clinical Study of Penpulimab-Based Combination Regimen for Neoadjuvant/Adjuvant Treatment of Resectable Locally Advanced Non-Small Cell Lung Cancer
Study Number	ALTER-L043
Version and Date	Version 3.0 Date September 7, 2022
Applicant	Tianjin Cancer Hospital
Co-organizer	Chia Tai Tianqing Pharmaceutical Group Co.
Nature of study	Exploratory study
Subjects	Stage IIB-IIIB(N2), radically resectable, driver gene-negative NSCLC
Purpose of the study	Primary objective: To observe the primary efficacy of Penpulimab-based combination regimen neoadjuvant/adjuvant for the treatment of resectable locally advanced NSCLC.
	Secondary Objective: To observe the safety of the Penpulimab-based combination regimen for the neoadjuvant/adjuvant treatment of resectable locally advanced NSCLC.
Study Endpoints	Primary study endpoint<br>• Major Pathologic Remission Rate (MPR) as assessed by the investigator, i.e., the percentage of residual surviving tumor cells in the tumor bed in the postoperative specimen≤ 10% based on the Pathologic Remission Assessment Criteria.<br>Secondary study endpoints<br>• Complete pathological remission pCR [defined as the absence of residual tumor cells (including lymph nodes free of tumor remnants) in postoperative tumor tissue specimens, based on the Pathological Remission Assessment Criteria], preoperative objective remission rate (ORR), 1-year event-free survival (1-y EFS%), event-free survival (EFS), overall survival (OS), as assessed by the investigator<br>• Safety: adverse events (AE)
Study Design.	This study was designed to evaluate and observe the primary efficacy and safety of the Penpulimab-based combination regimen neoadjuvant/adjuvant for the treatment of resectable locally advanced NSCLC

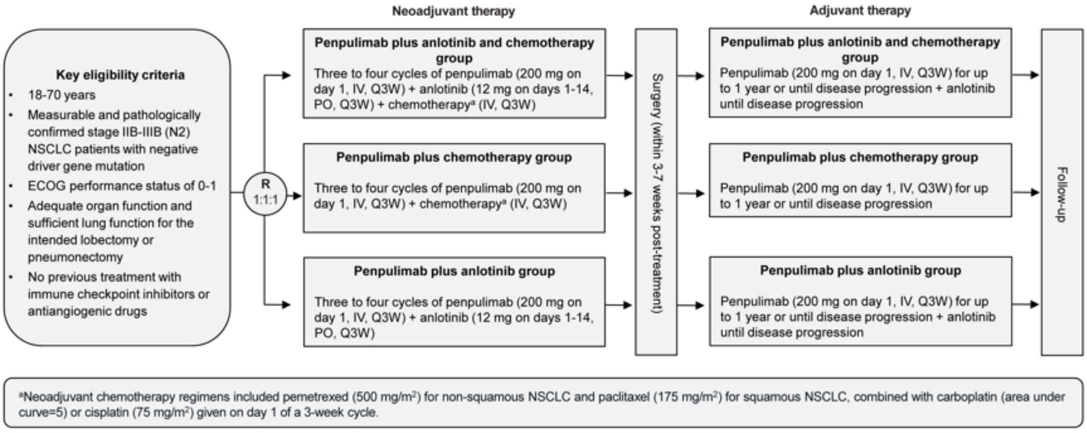

This was an exploratory study, and a total of 90 patients were enrolled and randomly assigned to group A/B/C, with 30 patients in each group.

Group A:

Neoadjuvant therapy (amlotinib+ Penpulimab, 4 cycles; chemotherapy 3-4 cycles): amlotinib, 12 mg, po, qd, continuous oral for 2 weeks with one week off, 3 weeks for 1 cycle; Penpulimab, 200 mg, iv, d1, 3 weeks for 1 cycle; chemotherapy (3-4 cycles): patients with non-squamous carcinoma chose pemetrexed+carboplatin/cisplatin (pemetrexed Pemetrexed,500mg/m$^{(2)}$,iv,d1,3 weeks for 1 cycle; Cisplatin,75mg/m$^{(2)}$,iv,d1,3 weeks for 1 cycle; Carboplatin AUC 5,iv,d1,3 weeks for 1 cycle), squamous carcinoma patients chose paclitaxel+carboplatin/cisplatin, (Paclitaxel,175mg/m$^{(2)}$,iv,d1,3 weeks for 1 cycle; Cisplatin,75mg/m $^{(2)}$,iv,d1,3 weeks as a cycle; carboplatin AUC 5,iv,d1,3 weeks as a cycle) (Carboplatin dose (mg): set AUC 5mg/ml/min× [urinary creatinine clearance (ml/min)+25],AUC:area under the curve).

Surgery was performed 3-7 weeks after the final dose of amlotinib, and was evaluated by the investigator for surgery

Adjuvant therapy 4-6 weeks postoperatively as assessed by the investigator

Adjuvant therapy: anilotinib, 12 mg, po, qd, 2 consecutive oral weeks off one week, 1 cycle of 3 weeks (until PD); Penpulimab, 200 mg, iv, d1, 1 cycle of 3 weeks (until PD or up to 1 year of treatment);

Group B:

Neoadjuvant therapy (Penpulimab, 4 cycles; chemotherapy 3-4 cycles):

	Penpulimab, 200 mg, iv, d1, 1 cycle in 3 weeks; chemotherapy (3-4 cycles): Patients with non-squamous cancers chose pemetrexed + carboplatin/cisplatin (pemetrexed, 500 mg/m$^{(2)}$,iv, d1, 1 cycle in 3 weeks; cisplatin, 75 mg/m$^{(2)}$,iv, d1, 1 cycle in 3 weeks; carboplatin AUC 5, iv, d1, 3 week for one cycle) and paclitaxel + carboplatin/cisplatin for patients with squamous carcinoma (paclitaxel,175mg/m$^{(2)}$,iv,d1,3 weeks for one cycle; cisplatin,75mg/m$^{(2)}$,iv,d1,3 weeks for one cycle; carboplatin AUC 5,iv,d1,3 weeks for one cycle) (Carboplatin dose (mg): the set AUC 5mg/ml/min× [urine creatinine clearance (ml/min)+25],AUC:area under the curve).<br>Surgery was performed 3-7 weeks after the final administration of Penpulimab and was evaluated by the investigator to be performed<br>Adjuvant therapy 4-6 weeks postoperatively as assessed by the investigator<br>Adjuvant therapy: penpulimab, 200 mg, iv, d1, 1 cycle of 3 weeks (until PD or up to 1 year of treatment);<br><br>Group C:<br>Neoadjuvant therapy (Anrotinib+ Penpulimab, 4 cycles): anrotinib, 12 mg, po, qd, continuous oral for 2 weeks off for one week, 3 weeks for 1 cycle; Penpulimab, 200 mg, iv, d1, 3 weeks for 1 cycle;<br>Surgery 3-7 weeks after final dose of amlotinib, as evaluated by the investigator<br>Adjuvant therapy 4-6 weeks postoperatively as assessed by the investigator<br>Adjuvant therapy: amlotinib, 12 mg, po, qd, 2 consecutive weeks by mouth with one week off, 1 cycle of 3 weeks (until PD); Penpulimab, 200 mg, iv, d1, 1 cycle of 3 weeks (until PD or up to 1 year of treatment);<br>All chemotherapeutic agents (carboplatin, cisplatin, pemetrexed, and paclitaxel) are required to be nationally consistently evaluated drugs.
Planned number of patients enrolled	90
Principal Investigator	Prof. Changli Wang
Organization in charge of the clinical	Tianjin Cancer Hospital

trial	
Patient Screening Criteria	**General Inclusion Criteria:**<br>• Subjects voluntarily enroll in the study, sign the informed consent form, good compliance, cooperate with follow-up visits<br>• Age≥ 18 years and≤ 70 years at the time of signing the informed consent form, male or female;<br>• Eastern Cooperative Oncology Group (ECOG) physical status score of 0 or 1;<br>• Expected survival of not less than 12 weeks;<br>• Male and female patients of childbearing age agree to use a reliable method of contraception before entering the trial, during the study and until 8 weeks after discontinuation.<br>• Consent to collect tumor histology specimens needed for the study and apply them to the study;<br>• Patients who agree to undergo radical surgical treatment;<br>• Patients who, in the judgment of the specialist, have no contraindications to surgery<br>**Disease-related inclusion criteria:**<br>• Patients with non-small cell lung cancer diagnosed by pathologic histology or cytology (according to the WHO 2015 classification); and patients with radically resectable stage IIB-IIIB (N2) non-small cell lung cancer (as judged by the International Association for the Study of Lung Cancer (IASLC) Manual of Thoracic Tumor Staging, 8th edition); and with primary or lymph node metastasis testing clearly EGFR/ALK/ROS1 negative (patients with squamous carcinoma will have the need for genetic testing at the discretion of the investigator);<br>• Subjects with primary non-small cell lung cancer not previously treated with surgery, chemotherapy, radiation therapy, and biologic therapy;<br>• Patients with resectable lesions as judged by the investigator, with clinical stage IIB-IIIB (N2), who can obtain sufficient tumor histology specimens (non-cytology) for molecular marker analysis;<br>• Evaluable disease with at least one single diameter measurable lesion with a longest diameter of ≥ 10 mm measured by spiral CT according to the criteria for the evaluation of the efficacy of solid tumors (RECIST 1.1)<br>• Subjects must have adequate lung function for the intended lung resection.<br>**Normal major organ function should meet the following criteria:**<br>• Pulmonary Ventilation Function Test, FEV1≥ 1.5L, or FEV1≥ 800 ml after anticipated lobectomy/total lung resection;<br>• Standard blood tests (no transfusion of blood or blood products within 14 days, not corrected

with G-CSF and other hematopoietic stimulating factors):

a) Hemoglobin (HB) ≥90g/L

b) Absolute neutrophil count (ANC) ≥1.5× 10^9/L

c) Platelets (PLT) ≥80× 10^9/L;

- Biochemical tests need to meet the following indicators:

a) Total bilirubin (TBIL) ≤ 1.5 times the upper limit of normal (ULN);

b) Alanine aminotransferase (ALT) and aspartate aminotransferase AST≤ 2.5× ULN;

c) Serum creatinine (Cr) ≤ 1.5× ULN or creatinine clearance (CCr) ≥ 60 ml/min.

- International normalized ratio (INR) of prothrombin time ≤ 1.5 and partial thromboplastin time (APTT) ≤ 1.5 times the upper limit of normal in patients who have not received anticoagulation therapy. Patients receiving full or parenteral anticoagulant therapy may be admitted to clinical trials as long as the dose of anticoagulant has been stable for at least 2 weeks prior to entry into a clinical study and the results of coagulation assays are within the local therapeutic limits;
- Women of childbearing potential (15～ 49 years of age) must have had a negative urine pregnancy test within 7 days prior to initiating treatment.

Exclusion Criteria:

- Large cell carcinoma and mixed cell lung cancer;
- Patients who, in the judgment of the investigator, have a high probability of fatal hemorrhage due to tumor invasion of vital blood vessels during subsequent studies; or who have significant cavitary or necrotic tumors in the lungs;
- Any systemic anticancer therapy, including cytotoxic drug therapy, immunologic drug therapy, or experimental therapy, for NSCLC;
- Have had localized radiotherapy for NSCLC;
- Patients who have had a cancer other than NSCLC in the five years prior to the start of treatment in this study. Excluding cervical carcinoma in situ, cured basal cell carcinoma, and bladder epithelial tumors [including Ta and Tis];
- Patients with prior use of amlotinib and other anti-angiogenic agents;
- Patients with prior use of Penpulimab, or other anti-PD-1, anti-PD-L1, anti-CTLA-4 antibodies, and any other antibody or drug therapy targeting the T-cell co-stimulatory or checkpoint pathways, such as ICOS or agonists (e.g., CD40, CD137, GITR, OX40, etc.);
- Hypersensitivity to amlotinib or Penpulimab or any component of the chemotherapeutic agent;
- Patients with multiple factors that interfere with oral administration of medications (e.g.,

	inability to swallow, chronic diarrhea, and intestinal obstruction); and<br>• Patients with the presence of any severe and/or uncontrolled medical condition, including:<br>d) Patients with suboptimally controlled blood pressure (systolic blood pressure ≥ 150 mmHg and diastolic blood pressure ≥ 100 mmHg);<br>e) Patients with class I or greater myocardial ischemia or myocardial infarction, arrhythmias (including QTc≥ 480ms) and class ≥2 congestive heart failure (New York Heart Association (NYHA) classification);<br>f) Abnormal coagulation (INR > 1.5 or prothrombin time (PT) > ULN + 4 seconds or APTT > 1.5 ULN), bleeding tendency or undergoing thrombolytic or anticoagulant therapy; Note: The use of low-dose heparin (daily dosage of 0.6 million in adults) or low-dose aspirin (daily dosage ≤ 100 mg) is permitted for prophylactic purposes, provided that the international normalized ratio of the prothrombin time (INR) ≤ 1.5 is used. ~12,000 U) or low-dose aspirin (≤ 100 mg daily) for prophylactic purposes.<br>g) Active or uncontrolled serious infections;<br>h) Cirrhosis, decompensated liver disease, active hepatitis or chronic hepatitis requiring antiviral therapy;<br>i) Renal failure requiring hemodialysis or peritoneal dialysis;<br>j) History of immunodeficiency, including being HIV-positive or having other acquired, congenital immunodeficiency diseases, or a history of organ transplantation;<br>k) Poorly controlled diabetes mellitus (fasting blood glucose (FBG) > 10 mmol/L);<br>l) Those with routine urinalysis suggestive of urinary protein ≥++ and confirmed 24-hour urine protein quantification > 1.0 g;<br>m) Patients with seizures and requiring treatment;<br>n) Prolonged unhealed wounds or fractures, etc;<br>o) Clinically significant hemoptysis (>50 ml per day) within 2 weeks prior to enrollment; or clinically significant bleeding symptoms or a definite bleeding tendency, such as gastrointestinal bleeding, bleeding gastric ulcer, fecal occult blood++ and above at baseline, or suffering from vasculitis;<br>• Pre-existing interstitial lung disease, drug-induced interstitial disease, radiation pneumonitis requiring hormonal therapy, or any clinically evidenced active interstitial lung disease;<br>• Those who have had an arterial/venous thrombotic event within 6 months, such as cerebrovascular accidents (including transient ischemic attacks), deep vein thrombosis, and

	pulmonary embolism;<br>• Presence of current peripheral neuropathy of ≥ CTCAE degree 2, except as a result of trauma;<br>• Patients requiring total right lung resection; subjects who have had major surgery or severe trauma have had the effects of surgery or trauma resolved for less than 14 days prior to enrollment at ;<br>• Patients who are participating in another clinical study or are less than 4 weeks from the end of treatment in a previous clinical study;<br>• Patients with mixed small cell lung cancer components;<br>• Have received a live or attenuated vaccine within 30 days prior to the first dose of Penpulimab or plan to receive a live or attenuated vaccine during the study period;<br>• Known history of severe hypersensitivity reactions to other monoclonal antibodies;<br>• Pregnant or lactating women;<br>• Prior history of definite neurologic or psychiatric disorders, including epilepsy or dementia;<br>• Patients who, in the judgment of the investigator, may have other factors that may force the mid-term termination of this study, such as other serious illnesses or serious laboratory test abnormalities or concomitant family or social factors that would compromise the safety of the subjects or the collection of trial data and samples.
Withdrawal Criteria	1. Subject voluntarily withdraws informed consent at any time;<br>2. Medical imaging or clinical features suggesting relapse of the disease;<br>3. Those who are unable to tolerate the toxicity of amlotinib after two dose downward adjustments;<br>4. Subjects who experience a pregnancy event during the course of the study;<br>5. The occurrence of any clinical adverse event, laboratory test abnormality, or other medical condition that results in the possibility that the subject may no longer benefit from continued dosing;<br>6. Subjects found to be ineligible after enrollment;<br>7. Other reasons why the investigator believes that the trial treatment cannot be continued.
Criteria for Termination of Study	Subjects must be terminated from study medication upon the occurrence of any of the following (including, but not limited to)<br>• An unintended, meaningful, or unacceptable risk to the subject is discovered;<br>• A significant failure of the protocol is discovered during the execution of the trial;<br>• The investigational drug/trial treatment is ineffective, or continuation of the trial is pointless;

	• Extreme difficulty in completing the trial due to, for example, significant lags in subject enrollment or frequent protocol deviations.
Exclusion Criteria	• Failure to administer the medication at the dose, method, and regimen specified in this study protocol (discontinuation of medication for a cumulative period of more than four weeks in a single dosing cycle will be recorded as a dropout);<br>• Those who were treated with other chemotherapy or experimental drugs other than this protocol during the trial;<br>• Those who did not meet the criteria and were included in error;<br>• Patients not on medication
Determination of sample size	Assuming an MPR of 42% for each arm, taking historical data (MPR of 19% for atalizumab monotherapy in LCMC3), taking α=0.05, β=0.2, and using the PASS15 software, and after a preset 10% dropout rate, it is proposed to enroll 30 subjects in each arm of the study, for a total enrollment of 90 subjects.
Study Duration	Anticipated start of trial: 2021__ August<br>Estimated time to completion of enrollment: 2023___Feb.<br>Anticipated Trial End Date: 2029___05___
Dosing regimen	• Anrotinib, 12 mg, po, qd, taken orally for 2 consecutive weeks with a one-week break in a 3-week cycle;<br>• Penpulimab, 200 mg, iv, d1, 1 cycle in 3 weeks;<br>• Pemetrexed, 500 mg/m2 , iv, d1, 1 cycle in 3 weeks;<br>• Paclitaxel, 175 mg/m2 , iv, d1, 1 cycle in 3 weeks;<br>• Cisplatin, 75 mg/m2, iv, d1, 1 cycle in 3 weeks;<br>• Carboplatin AUC 5, iv, d1, 3 weeks for 1 cycle;<br>(Carboplatin dose (mg): set AUC 5 mg/ml/min× [urinary creatinine clearance (ml/min) + 25],AUC: area under the curve).

Clinical study flow chart

Visits	Screening phase visit [1]		Preoperative treatment visit [2]				Postoperative visit [3]	Postoperative treatment period visit [4](first year)	Postoperative observation period visit [5](Years 2 to 5)	Safety follow-up ([6) (])	Survival follow-up [7]
	-28d to -7d	-7d to -1d	C1D21± 3d	C2D21± 3d	C3D21± 3d	Preoperative evaluation Within 7 days before surgery	After surgery 4-6 weeks	Every 12 weeks± 7d	Every 24 weeks ± 7d	Every 48 weeks ± 7d	Every 48 weeks ± 7d
Signed Informed Consent	X										
Demographic information	X										
Entry/exit criteria	X										
Physical examination [8]	X		X	X	X	X	X	X			
Vital Signs [9]		X	X	X	X	X	X	X	X		
History of tumor and other medical history [10]	X										
Blood count [11]		X		X	X	X	X	X	X		
Urine routine [12]		X		X	X	X	X	X	X		
Routine [13]		X		X	X	X	X	X	X		
Blood biochemistry [14]		X		X	X	X	X	X	X		
Thyroid function	X			X	X	X	X	X	X		

Visits	Screening phase visit [1]		Preoperative treatment visit [2]				Postoperative visit [3]	Postoperative treatment period visit [4](first year)	Postoperative observation period visit [5](Years 2 to 5)	Safety follow-up ([6) (])	Survival follow-up [7]
	-28d to -7d	-7d to -1d	C1D21± 3d	C2D21± 3d	C3D21± 3d	Preoperative evaluation Within 7 days before surgery	After surgery 4-6 weeks	Every 12 weeks± 7d	Every 24 weeks ± 7d	Every 48 weeks ± 7d	Every 48 weeks ± 7d
[15]											
**Coagulation** [16]		X		X	X	X	X	X	X		
**Tumor markers** [17]	X			X	X	X	X	X	X		
**12-lead electrocardiogram** [18]		X		X		X	X				
**Cardiac enzyme profile** [19]		X									
**Echocardiography** [20]	X										
**HIV, Hepatitis B, Hepatitis C screening** [21]	X										
**Pregnancy test** ([22) (])	X										
**Imaging evaluation** ([2) (3) (])				X		X		X	X		
**Abdominal ultrasound**						X					
**Pulmonary function tests** ([24) (])	X					X	X				

Visits	Screening phase visit [1]		Preoperative treatment visit [2]				Postoperative visit [3]	Postoperative treatment period visit [4](first year)	Postoperative observation period visit [5](Years 2 to 5)	Safety follow-up ([6) ([)	Survival follow-up [7]
	-28d to -7d	-7d to -1d	C1D21± 3d	C2D21± 3d	C3D21± 3d	Preoperative evaluation Within 7 days before surgery	After surgery 4-6 weeks	Every 12 weeks± 7d	Every 24 weeks ± 7d	Every 48 weeks ± 7d	Every 48 weeks ± 7d
**Pathologic evaluation ([25) ([)**	X						X				
**PD-L1 testing ([26) ([)**	X						X				
**ECOG score**		X	X	X	X	X	X	X			
**Blood pressure monitoring ([2) (7) ([)**	X	X	X	X	X						
**Adverse Events ([2) (8) ([)**			X	X	X					X	
**Dispensing/Recall of Medication ([2) (9) ([)**		X	X	X	X			X	X		
**Record concomitant medications ([) (30) ([)**	X		X	X	X	X	X	X	X	X	
**Medication adherence ([3) (1) ([)**	X		X	X	X			X	X		
**Time to disease progression ([3) (2) ([)**											X
**Time to death**											X
**Tumor treatment status ([3) (3) ([)**										X	X

Note: Each examination and test procedure was performed according to the timing of the study flow chart, independent of the length of drug withdrawal, but allowing for occasional

changes within the window of each examination due to holidays, vacations, or other administrative reasons.

[1]. Screening Visit: subjects must complete the screening visit component within 28-7 days prior to enrollment.

[2]. Preoperative Treatment Period Visit: 21 day± 3 day visit in Cycle 1, 21 day± 3 day visit in Cycle 2, 21 day± 3 day visit in Cycle 3, and one preoperative evaluation within 4 weeks (within 28 days) of the final dose.

[3]. Postoperative visits: one visit 4-6 weeks (21-42 days) postoperatively.

[4]. Postoperative treatment period visits: every 12 weeks± 7 days for one year postoperatively.

[5]. Postoperative observation period visits: every 24 weeks± 7 days for the 2nd to 5th year postoperatively.

[6]. Safety Follow-Up: 21-day safety follow-up after the patient's last dose.

[7]. Survival follow-up is performed at the end of the postoperative observation period visit into survival follow-up every 48 weeks ± 7 days.

[8]. Physical examination: height, weight, and examination of body systems (head, face, skin system, lymph nodes, respiratory system, cardiovascular system, abdomen, musculoskeletal, neurologic system, and mental status).

[9]. Vital signs: temperature, respiration, heart rate, and blood pressure monitoring.

[10]. Tumor history and other medical history: detailed questions about previous antitumor therapy: time of initiation of first-line chemotherapy, regimen, and duration of medication (including optimal efficacy, date of treatment failure/disease progression, and development of grade 3 or higher toxicity); disease stage prior to enrollment, history of smoking, and history of treatment of disease.

[11]. Blood count: hemoglobin, red blood cells, white blood cells, neutrophil count, lymphocyte count, and platelet count.

[12]. Urine routine: urinary protein, urinary glucose, urinary occult blood (urinary erythrocytes, leukocytes), urinary pH, and urinary ketone bodies. If a semiquantitative method shows urinary protein ≥2+ (e.g., urine test strip), a 24-hour quantitative urine protein test is performed.

[13]. Stool routine: occult blood.

[14]. Blood biochemistry: total bilirubin, conjugated bilirubin, ALT, AST, total protein, albumin, urea nitrogen, creatinine, uric acid, blood glucose, potassium, sodium, chloride, calcium.

[15]. Thyroid function: tsh, free t4, free t3.

[16]. Coagulation function: PT, APTT, TT, Fbg, INR.

[17]. Tumor markers: serum CEA, neurenolase NSE

[18].12-lead ECG: pay special attention to the QT interval. If ECG abnormalities are detected 2 additional confirmations must be performed (each 5 minutes apart, QT interval time should be labeled).

[19]. Cardiac enzyme profile: one test was done within the first 7 days of enrollment, and this study was supplemented thereafter only if symptoms such as precordial pain and palpitations, as well as ECG abnormalities, were present.

[20]. Echocardiography: one study within 7 days prior to enrollment, to be supplemented thereafter only if clinically significant symptoms such as ECG abnormalities, pain in the precordial area, or palpitations occur during treatment.

[21].HIV, Hepatitis B, Hepatitis C screening: two-half-pair test for Hepatitis B and Hepatitis C virus antibody (anti-HCV) test.

[22]. Pregnancy test: serum pregnancy test is used only for women of childbearing age.

[23]. Imaging evaluation:

a) Imaging methods during the screening period are standardized to the PET-CT method of testing. Tumor evaluations can be up to 4 weeks prior to the first dose of study drug, PET-CT results obtained prior to signing informed consent can be used for screening phase tumor evaluations as long as they meet the requirements of RECIST 1.1.
b) Imaging methods during the visit period are standardized to CT; brain MRI is required when brain metastases are suspected and diagnosed (in cases where MRI is contraindicated, PET-CT may be used instead), and bone scans are performed only when clinically indicated.
c) Comprehensive imaging evaluation should be performed within 7 days before lung cancer surgery.
d) New lesions suspected at a later stage should be examined in due course.
e) Timely imaging evaluation is required when subjects are discharged from the cohort for any reason.
f) The imaging evaluation schedule allows a window of ± 4 days. Unscheduled imaging assessments may be performed when disease progression is suspected (e.g., worsening of symptoms).
g) In addition to disease progression confirmed by imaging, subjects who end treatment for other reasons should also be imaged as often as possible at the frequency specified

in the protocol until documented confirmation of disease progression, initiation of new antitumor therapy, or death.

[24]. Lung function assessment: normal or mildly to moderately abnormal lung function (VC%>60%, FEV1>1.2L, FEV1%>40%, DLco>40%) to tolerate pneumonectomy; assessed within 7 days prior to the first dose, 7 days prior to pneumonectomy, and once postoperatively.

[25]. Pathologic assessment: diagnostic pathology assessment within 28 days prior to first dose, and assessment of pathological remission after lung cancer resection to clarify whether it is MPR or pCR.

[26]. Immunohistochemical detection using IHC 22C3 PharmDx (DAKO)

[27]. Blood pressure monitoring: blood pressure monitoring was done by the patients themselves and recorded by themselves, blood pressure was tested at least 3 times per week in the first 2 cycles of blood pressure, and if the blood pressure was abnormal, it was followed up every day; if the blood pressure was normal, it was tested 2 times per week after the 2 cycles of blood pressure; in addition, at each follow-up visit, the investigator then measured the blood pressure, and when the blood pressure measurement was done, smoking and coffee drinking were prohibited for 30 minutes before the measurement, and quiet rest was taken for at least 10 minutes The blood pressure was measured in a seated position with the elbow at the same level as the heart, and each blood pressure measurement was taken on the same side of the body.

[28]. Adverse events: including nausea, vomiting, diarrhea, and abdominal distension.

[29]. Dispensing/recall of medications: actual medication use, there is a possibility of dose downward adjustment, and complimentary dispensing of anrotinib with penpulimab is done on a per-cycle basis.

[30]. Recording of concomitant medications: concomitant medications and concomitant treatments within the first 28 days of enrollment and during the study period were recorded. Once a subject discontinues trial treatment, only concomitant medications and concomitant treatments used for new or unresolved adverse events related to trial treatment should be recorded.

[31]. MEDICATION ADHERENCE: At the beginning of the first day of each treatment cycle, medication doses, counts, and adherence for the previous cycle are calculated and recorded in the CRF.

[32]. Time to disease progression: for patients with non-imaging evidence of progression (intolerable, other conditions), imaging evaluations should be continued every 6 weeks until disease progression, initiation of other oncologic treatments, death, or end of the study (telephone or clinical follow-up is acceptable for out-of-hospital reports).

[33]. Tumor treatment: survival follow-up to document tumor treatment.

List of abbreviations

Abbreviations and full spelling	Chinese
AACR(American Association of Cancer Research)	American Association of Cancer Research
ACE-I (Angiotensin Converting Enzyme Inhibitors)	Angiotensin Converting Enzyme Inhibitors
ADR (Adverse Drug Reactions)	Adverse Drug Reactions
AE (Adverse Event)	Adverse Event (Adverse Event)
AKP (Alkline Phosphatase)	Alkaline Phosphatase
ALK (Anaplastic Lymphoma Kinase)	Anaplastic Lymphoma Kinase
ALT (Alanine amiotransferase)	ALT (Alanine aminotransferase)
ANC (Absolute Neutrophil Count)	Neutrophil Count
APTT (Activated Partial Thromboplastin Time)	Activated Partial Thromboplastin Time
ARB (Angiotensin Receptor Blocker)	Angiotensin II Receptor Blocker
ASCO (American Society of Clinical Oncology)	American Society of Clinical Oncology
AST(Aspartate Aminotransferase)	Aspartate Aminotransferase (AST)
BIL (Bilirubin)	Bilirubin
BSC Best Supportive Care	Best Supportive Care
BUN (Blood Urea Nitrogen)	Urea Nitrogen
CCr (Creatinine Clearance)	Creatinine Clearance
CL (Clearance)	Clearance
CR (Complete Response)	Complete Response
Cr (Creatinine)	Creatinine
CRF (Case Report Form)	Case Report Form
CSCO (Chinese Society of Clinical Oncology)	Chinese Society of Clinical Oncology (CSCO)
CT (Computed Tomography)	Computed Tomography
CTC AE (Common Terminology Criteria for Adverse Events)	Common Terminology Criteria for Adverse Events
CTLA-4 (Cytotoxic T-lymphocyte-Associated	Cytotoxic T-lymphocyte-Associated

Abbreviations and full spelling	Chinese
Protein-4)	Protein-4
DCR (Disease Control Rate)	Disease Control Rate
DLT (Dose Limiting Toxicity)	Dose Limiting Toxicity
DRQ (Data Request Query)	Data Request Query
ECG (Electro Cardio Gram)	Electrocardiogram (ECG)
ECOG (Eastern Cooperative Oncology Group)	Eastern Cooperative Oncology Group
EDC (Electronic Data Capturing)	Electronic Data Capturing
EFS (Event Free Survival)	Event Free Survival
EGFR (Epidermal Growth Factor Receptor)	Epidermal Growth Factor Receptor
EORTC (The European Organization for Research and Treatment for Cancer)	The European Organization for Research and Treatment for Cancer
FAS (Full Analysis Set)	Full Analysis Set
Fbg (Fibrinogen)	Fibrinogen
Fc (Fragment Crystallizable)	Fragment Crystallizable
GCP (Good Clinical Practice)	Good Clinical Practice
GLP (Good Laboratory Practice)	Good Laboratory Practice (GLP)
Glu (Glucose)	Glucose
Hb (Hemoglobin)	Hemoglobin
HCV (Hepatitis C virus)	Hepatitis C virus
HFS (Hand-Foot Syndroms)	Hand-Foot Syndrome (HFS)
HIV (Human immunodeficiency virus)	Human immunodeficiency virus
HR (Hazard Ratio)	Risk Ratio
IDMC (Independent Data Monitoring Committee)	Independent Data Monitoring Committee
INR (International Normalized Ratio)	International Normalized Ratio
irAE (Immune-related Adverse Events)	Immune-related Adverse Events
mAb (monoclonal antibody)	Monoclonal antibody
MPR (Major Pathologic Response)	Major Pathologic Response
MRI (Magnetic Resonance Imaging)	Magnetic Resonance Imaging (MRI)
MTD (Maximum Tolerated Dose)	Maximum Tolerated Dose
NMPA(National Medical Products	State Drug Administration (SDA)

Abbreviations and full spelling	Chinese
Administration)	
NSAID(Nonsteroidal Antiinflammatory Drugs)	Nonsteroidal Anti-inflammatory Drugs
NSCLC(Non-Small Cell Lung Cancer)	Non-Small Cell Lung Cancer
NYHA (New York Heart Association)	New York Heart Association
OB (Occult Blood)	Occult Blood
ORR (Objective Response Rate)	Objective Response Rate
OS (Overall Survival)	Overall Survival
PBMC (Peripheral blood mononuclear cell)	Peripheral blood mononuclear cell
pCR (Pathologic Complete Response)	Pathologic Complete Response
PD (Pharmacodynamics)	Pharmacodynamics
PD (Progressive Disease)	Progression
PD-1 (Programmed Cell Death-1)	Programmed Cell Death-1
PDGFR (Platelet-derived Growth Factor Receptor)	Platelet-derived growth factor receptor
PD-L1 (Programmed Cell Death-1 Ligand 1)	Programmed Cell Death-1 Ligand-1 (Programmed Cell Death Protein-1-Ligand-1)
PFS (Progression-Free Survival)	Progression-Free Survival
PI (Principal Investigator)	Principal Investigator
PK (Pharmacokinetics)	Pharmacokinetics
PLT (Platelets)	Platelets
PPS (Per Protocol Set)	Protocol Set Compliant
PR (Partial Response)	Partial Remission
PRO (Protein)	Protein
PS (Performance Status)	Physical Performance Status
PT (Prothrombin Time)	Prothrombin Time
RBC (Red Blood Cell)	Red Blood Cell
RECIST (Response Evaluation Criteria in Solid Tumor)	Response Evaluation Criteria in Solid Tumor
RR (Respose Rate)	Remission Rate
RTKs (Receptor Tyrosine Kinase)	Receptor Tyrosine Kinases

Abbreviations and full spelling	Chinese
SAE (Serious Adverse Event)	Serious Adverse Event
SAS (Safty Analysis Set)	Safety Analysis Set
SCLC (Small Cell Lung Cancer)	Small Cell Lung Cancer
SCr (Serum Creatinine)	Serum Creatinine
SD (Stable Disease)	Stable Disease
TBIL (Total Bilirubin)	Total Bilirubin
TKI (Tyrosine Kinase Inhibitor)	Tyrosine Kinase Inhibitor
TRAE (Treatment-Related Adverse Events)	Treatment-Related Adverse Events
TSH (Thyroid Stimulating Hormone)	Thyroid Stimulating Hormone
TT (Thrombin Time)	Thrombin Time
ULN (Upper Limit Of Normal)	Upper Limit Of Normal
VEGF (Vascular Endothelial Growth Factor)	Vascular Endothelial Growth Factor
VEGFR (VEGF Receptor)	Vascular Endothelial Growth Factor
WBC (White Blood Cell)	White Blood Cell
WCLC (World Conference on Lung Cance)	World Conference on Lung Cancer
WHO (World Health Organization)	World Health Organization
γ-GT (γ- Glutamyltransferase)	γ-Glutamyltransferase

1. Background of the study

This study was organized by Tianjin Cancer Hospital, in accordance with the national "Administrative Measures for Drug Registration" as well as the "Good Clinical Practice (GCP) for Quality Management of Drug Clinical Trials" and the information of chemical composition, pharmacokinetics, pharmacodynamics, and toxicology studies of anilotinib hydrochloride and Penpulimab, the combination of Penpulimab-based regimen for neoadjuvant/adjuvant treatment of resectable locally advanced non-small cell lung cancer in an exploratory phase II clinical study, this study protocol is developed.

1.1 Study feasibility

1.1.1 Epidemiology of lung cancer and current status of treatment

Lung cancer is the malignant tumor with the highest morbidity and mortality rate worldwide ([) (1]), with about 2,093,000 new lung cancer patients and 1,761,000 deaths globally annually, and about 787,000 and 631,000 new lung cancer cases and deaths in China in 2015, respectively ([) (2]). The incidence of lung cancer may further increase with the intensification of economic development, environmental pollution and population aging. In recent years, the incidence and mortality rates of lung cancer in China have shown a rapid growth trend, and it is expected that by 2025,

the number of lung cancer cases will reach 1 million, with an increasingly serious disease burden.

Based on the biological characteristics, treatment and prognosis of lung cancer, the World Health Organization (WHO) classifies it into two main categories: non-small cell lung cancer (NSCLC) and small cell lung cancer (SCLC). NSCLC accounts for about 80-85% of all lung cancer patients, and the main treatment modalities for NSCLC are surgery, radiotherapy, targeted therapy and immunotherapy. Surgery is still the first choice for patients with curable NSCLC, which significantly improves the quality of life of patients and prolongs the overall survival period. Preoperative neoadjuvant therapy for operable NSCLC patients can significantly reduce tumor stage, inhibit tumor cell recurrence and metastasis, reduce the difficulty of surgical operation, increase the rate of complete tumor resection, and reduce postoperative complications. From three SAKK studies [3](SAKK 16/96, SAKK 16/00, and SAKK 16/01), a total of 368 patients with stage IIIA-IIIB were enrolled in surgery after neoadjuvant treatment with platinum-containing two-drug or platinum-containing two-drug sequential radiotherapy, and the results showed that the 5-year and 10-year survival rates of patients with stage IIIA were 41% and 29%, and those with stage IIIB had a 5- and 10-year survival rates of 35% and 27%, respectively (TNM staging system, version 8). Preoperative neoadjuvant therapy combined with surgical treatment is of great significance for patients with stage II-III NSCLC and provides a reliable theoretical basis for clinical work: ① Reduce the tumor stage and shrink the tumor volume, thus increasing the surgical resection rate; ② Control and treat tiny metastases in vivo to reduce the recurrence rate; ③ Chemotherapeutic agents pass through the undamaged blood supply system to reach the tumor tissues in sufficient amounts; ④ Assess the in vivo sensitivity of chemotherapeutic agents to guide the (iv) assessthe in vivo sensitivity of chemotherapeutic agents to guide postoperative treatment ([) (4) (]). Meanwhile such patients receiving adjuvant therapy after surgery may also bring longer survival benefit to patients, in 2008 LACE [5]included a meta-analysis of five randomized controlled studies (ALPI/BLT/IALT/JBR10/ANITA) with a total of 4,584 patients aiming at evaluating the efficacy of adjuvant platinum-containing chemotherapy after surgery for NSCLC, and the results showed a significant benefit in OS (HR=0.89,P=0.005), this study established the status of adjuvant chemotherapy.

Currently, chemotherapy is still the mainstay of the adjuvant phase of treatment, with occasional targeted therapy, immunotherapy, and other related studies underway, and this direction may also be explored as a new treatment modality in the search for better therapeutic options.

1.1.2 Advances in immuno(neo)adjuvant therapy in NSCLC

Immunotherapy has become one of the important treatments for advanced lung cancer, and also shows good efficacy in (neo)adjuvant therapy for patients with early-stage lung cancer.The 2019 World Conference on Lung Cance (WCLC) reported that neoadjuvant therapy with sindilizumab in a Chinese population of patients with stage IA-IIIB NSCLC An exploratory phase II study of adjuvant therapy ([) (6) (]), evaluable 40 patients all received 2 cycles of sindilizumab neoadjuvant therapy, and of the 37 patients who underwent surgical resection, 15 (40.5%) achieved MPR, and 6 (16.2%) achieved pathologic complete response (pCR). Updated results from the open, single-arm, phase II LCMC3 study ([) (7) (]), evaluating atalizumab as a driver mutation-free phase IB/II or IIIA and selected IIIB at the 2019 American Society of Clinical Oncology (ASCO) and WCLC congresses were presented resectable NSCLC patients with neoadjuvant and adjuvant therapy, 4 (5%) patients achieved pCR and 15 (19%) patients achieved MPR among 77 patients who underwent surgical resection after neoadjuvant treatment with atalizumab.In another meta-analysis of immune drug neoadjuvant therapy for operable-stage NSCLC ([) (8) (])study, a total of 252 patients from seven studies, the efficacy of neoadjuvant immunotherapy was evaluated using MPR and pCR, and the safety evaluation metrics included the incidence of Treatment-Related Adverse Events (TRAE), surgical resection rate, surgical complication rate, and surgical delay rate. The results showed that the MPR and safety of neoadjuvant immunotherapy were superior to that of chemotherapy, with a surgical resection rate of 88.70% (OR=7.61; 95% CI, 4.90-11.81), which was similar to the previously reported surgical resection rate of 75-90% for neoadjuvant chemotherapy. It can be concluded from the above clinical studies that neoadjuvant immunotherapy regimens have significant efficacy and safety controllable characteristics in patients with resectable NSCLC.

1.1.3 Research progress of immunization combined with amlotinib for NSCLC treatment

A large number of basic and clinical studies have shown that immune combined antiangiogenic therapy has achieved better results than monotherapy in a variety of solid tumors, and that angiogenesis and immunosuppression influence each other and promote each other ([) (9]), and that antiangiogenesis combined with immunotherapy exerts synergistic antitumor effects [10], and that immunosuppression recruits immunosuppressive cells through the release of cytokines, such as CCL2, from tumor cells, which releases vascular Vascular Endothelial Growth Factor A (VEGFA) and other pro-angiogenic factors act on vascular endothelial cells, and ultimately form neovascularization; VEGFA and other growth factors reduce the adhesion and migration of immune cells, and inhibit the maturation of DC cells and the normal differentiation of hematopoietic precursor cells; and promote the programmed cell death protein-1-ligand in DC cells. expression of Programmed Cell Death-1 Ligand 1 (PD-L1); activation of Treg cells via NRP1, thereby inducing immunosuppression.

The results of a study evaluating intratumoral immune changes after treatment with amlotinib in a homologous lung cancer mouse model showed ([) (1) (1) (]): the combination of amlotinib with a Programmed Cell Death-1 (PD-1) inhibitor treatment group significantly increased the inhibitory effect on tumor growth. The effect of amlotinib on intrinsic immune cells in the tumor microenvironment and the potential synergistic antitumor effect with immune checkpoint inhibitors were confirmed. Based on the confirmation of various theoretical foundations, there are now a variety of studies of immune-combination amlotinib for advanced NSCLC, a study of amlotinib in combination with sindilizumab in patients with advanced NSCLC announced by WCLC 2019 ([1) (2) (]), which enrolled a total of 22 driver gene-negative, untreated patients with stage IIIB-IV NSCLC, and the phase I results showed that the overall ORR was 72.7%, with 16 patients in PR and 6 patients in SD; the Disease Control Rate (DCR) of all patients was 100%, and 81.8% of patients were still receiving treatment at the time of data cutoff (July 3, 2019). The 6-month Progression-Free Survival (PFS) rate was 93.8% in all patients. This result also confirms the effectiveness of anilotinib in combination with sindilizumab in patients

with advanced NSCLC in the clinic.

1.1.4 Progress of Penpulimab-related studies

Penpulimab's efficacy and safety have been confirmed in preclinical studies, as detailed in the< 2. Investigational Drugs> section. Current studies in progress are shown in the table below:

Country	Program number	Phase	Experimental design	Status
Australia	AK105-101	Phase I	Phase I Dose Crawl and Dose Extension Clinical Trial in Solid Tumors	In Progress
China	AK105-201*	Phase Ib/II	Single-Arm Study for the Treatment of Relapsed or Refractory Classic Hodgkin's Lymphoma	NDA
China	AK105-202*	Phase II	Single-Arm Study to Treat Metastatic Nasopharyngeal Carcinoma Failing Second-Line Chemotherapy (soon to be pre- NDA)	Ongoing
China	AK105-203	Phase II	A Single-Arm Study of Combination Anilotinib for First-Line Treatment of Unresectable Hepatocellular Carcinoma	Ongoing
China	AK105-204	Phase Ib/II	Single-Arm Study for the Treatment of Selected Advanced or Metastatic Solid Tumors	Ongoing
China	AK105-301*	Phase III	A Randomized Controlled Study of Combination Carboplatin Plus Pemetrexed and Combination Anilotinib for First-Line Treatment of Metastatic Non-Squamous Non-Small Cell Lung Cancer	Ongoing
China	AK105-302*	Phase III	A Randomized Controlled Study of Combining Paclitaxel Plus Carboplatin in the First-line Treatment of Metastatic Squamous Non-Small Cell Lung Cancer (Enrollment Closing)	Ongoing

*Registration study

1.1.5 Current Phase II/III study of neoadjuvant/adjuvant immune combination therapy for NSCLC Ongoing.

NCT	Study Phase	Samp le size	Drug	Treat ment Phase	Study Endpoin t

NCT02998528	III	350	Platinum-containing two-agent chemotherapy<br>Navulizumab+ Platinum-containing two-agent chemotherapy<br>Navulizumab + Ipilimumab	Neoadjuvant	EFS, pCR
NCT03425643	III - 786	786	Neoadjuvant: pembrolizumab+ platinum-containing two-agent chemotherapy; placebo+ platinum-containing two-agent chemotherapy<br>Adjuvant: pembrolizumab; placebo	Neoadjuvant+ Adjuvant	EFS, OS
NCT03456063	NCT03456063	450	Neoadjuvant: atilizumab + platinum-based chemotherapy; placebo+ platinum-based chemotherapy<br>Adjuvant: atalizumab; BSC	Neoadjuvant+ Adjuvant	MPR, EFS
NCT03800134	III	800	Duvarizumab + Platinum-based chemotherapeutic agent<br>Placebo + Platinum-based chemotherapeutic agents	Neoadjuvant	MPR, EFS
NCT04506242	II	74	Neoadjuvant: karelizumab+ Apatinib<br>Adjuvant: carrelizumab	Neoadjuvant+ Adjuvant	MPR
NCT04379739	II	82	Neoadjuvant: karelizumab + Apatinib/platinum-based chemotherapy	Neoadjuvant	MPR
NCT03872661	II	36	Neoadjuvant: Sindilizumab+ Bevacizumab+ Chemotherapy	Neoadjuvant	Surgical resection rate
NCT04379635	III	380	Neoadjuvant: tirilizumab/placebo cisplatin or carboplatin+ paclitaxel or pemetrexed<br>Adjuvant: tirilizumab/placebo	Neoadjuvant+ Adjuvant	MPR

Based on the above results and rationale, this study hopes to bring further clinical benefit to patients with resectable locally advanced NSCLC by utilizing an exploratory phase II clinical study of neoadjuvant/adjuvant treatment of resectable locally advanced NSCLC with a combination regimen based on Penpulimab.

2. Study Drugs

2.1 Amrotinib hydrochloride

2.1.1 Pharmacologic studies of amlotinib hydrochloride

Anrotinib hydrochloride is a multi-targeted receptor tyrosine kinase (RTK) inhibitor. The results of kinase inhibition assays showed that amlotinib inhibited the kinase activity of VEGFR1 (IC50 of 26.9 nM), VEGFR2 (IC50 of 0.2 nM), VEGFR3 (IC50 of 0.7 nM), c-Kit (IC50 of 14.8 nM), and PDGFRβ (IC50 of 115 nM).

The results of in vitro assays showed that amlotinib inhibited the proliferation of various tumor cell lines (786-O, A375, A549, Caki-1, U87MG, MDA-MB-231, HT-29, NCI-H526, and HMC-1), with IC50s ranging from 3.0 to 12.5 μM; and significantly inhibited, in HUVECs cells, the VEGFR2 phosphorylation level of VEGFR2 and downstream phosphorylation of related proteins in HUVECs cells, significantly inhibited the phosphorylation level of c-Kit and downstream phosphorylation of related proteins in Mo7e cells, and significantly inhibited the phosphorylation level of PDGFR and downstream phosphorylation of related proteins in U87MG cells; significantly inhibited the proliferation, migration, and tubule formation of VEGF-A-stimulated HUVECs; and inhibited the formation of microvessel-like structures of rat arteries. It can inhibit the formation of ring microvessel-like structures in rat arteries.

2.1.2 Anrotinib hydrochloride Toxicological studies

General toxicology: SD rats were given 0.2, 0.8 and 3.0 mg/kg of amlotinib orally for 26 consecutive weeks, and the drug was stopped and recovered for 6 weeks, the NOAEL was 0.8 mg/kg, which was about 0.65 times the clinical dose (12 mg/person) based on body surface area; a significant toxic reaction was observed at 3.0 mg/kg, and the toxic target organs were teeth and kidneys. Kidneys.Beagle dogs were given amlotinib 0.02, 0.08 and 0.32 mg/kg orally for 39 consecutive weeks,

with 6 weeks of discontinuation for recovery, and NOAEL＜ 0.02 mg/kg, which was approximately 0.05 times the clinical dose of the drug (12 mg/human) on a body surface area basis, with the main toxic effects being arteritis of the small arteries/microarterioles and its secondary alterations.The main toxic effects of amlotinib in dogs are the following: aortic and microarteriolar arteritis and its secondary alterations.

Genotoxicity: The results of Amrotinib Ames test, Chinese hamster lung fibroblast (CHL) chromosome aberration test and mouse bone marrow micronucleus test were negative.

Reproductive toxicity: In fertility and early embryonic development toxicity tests, rats given 0.25, 1.0, and 4.0 mg/kg of amlotinib orally were seen to have reduced bilateral epididymis volume (1/24), mild~ mild prostate atrophy (10/24), and mild~ moderate seminal vesicle gland atrophy (13/24) in males; and luteal body counts, luteal body counts, gestational luteal body counts, gestational pregnancy, conception rates, placenta, and uterus in females, ovarian weights or coefficients were reduced, uterine gland and gestational corpus luteum atrophy and corpus luteum cysts were seen, number of fetuses absorbed, pre/post/total loss of conception rate were elevated, and number of live fetuses was reduced. The NOAEL for this test was 1 mg/kg (approximately 0.8 times the clinical dose of 12 mg/person based on body surface area).

In the embryo-fetus developmental toxicity test, pregnant rats were given 0.3, 0.6, and 1.8 mg/kg orally of amlotinib, which was seen as a reduction in the weight of the placenta of the live fetus, an increase in the early resorption of the fetus, an increase in the post-implantation loss, a reduction in the weight of the gestational uterus, a small individual fetus, a significant increase in the number of teratogens and the number of litters where the teratogenous fetuses occurred (179/200 fetuses and 22/22 litters), and a decrease in the major teratogens manifesting themselves as hydatidiforms The main malformations were edema, short tail or no tail, curled tail;

increased incidence of enlarged ventricles in fetuses; decreased ossification points of tail vertebrae, sternal pedicle, raphe, metacarpals and proximal fingers (metatarsals) in fetuses; increased incidence of underdevelopment of the third and fourth sternum, cranial, lumbar and thoracic vertebrae in fetuses; and increased incidence of rib malformations. The test NOAEL＜ 0.3 mg/kg (approximately 0.25 times the clinical dose of 12 mg/person based on body surface area).

In pregnant rabbits, oral administration of amlotinib 0.15, 0.3, and 0.9 mg/kg was seen to result in a decrease in the weight of the pregnant uterus, placental uterus, placenta, and uterine and ovarian organs, a decrease in the number of corpus luteums, the number of implantation glands, and the pregnancy rate, an increase in the percentage of pregnant rabbits with resorbed fetuses, a decrease in the number of live fetuses, an increase in the number of resorbed fetuses, a higher rate of lost fetuses, and delayed fetal litter development (decrease in the weight and number/degree of ossification of the skeleton),. Elevated rates of cosmetic, visceral, and skeletal variability or malformations. The test NOAEL＜ 0.15 mg/kg (approximately 0.25 times the clinical dose of 12 mg/person based on body surface area).

In the perinatal reproductive toxicity test, rats given 0.3, 0.6, and 1.8 mg/kg of amlotinib orally were seen to die in the parental dams, with an increase in the number of absorbed fetuses, the number of stillborn fetuses, the percentage of pregnant rats with stillborn fetuses, and a decrease in body weights and intake of food, and pregnancy rates; and a decrease in the birth survival rate, nursing viability, and body weights of the offspring rats. The NOAEL for parental dams and F1 generation rats was 0.6 mg/kg (about 0.5 times the clinical dose of 12 mg/human based on body surface area). Anrotinib is secreted into breast milk, where its concentration is approximately 30-50% of the blood concentration.

Carcinogenicity: No carcinogenicity studies have been conducted with amlotinib

2.1.3 Pharmacokinetic Studies of Anrotinib Hydrochloride

Absorption

Twelve healthy subjects were administered 5 mg of amlotinib capsules orally on an empty stomach; the mean time to peak plasma concentration of amlotinib was 9.3 hours, and elimination in vivo was slower, with a mean elimination half-life of 113 hours. A high-fat diet reduces the oral bioavailability of amlotinib hydrochloride capsules, and the total in vivo exposure of amlotinib when taken concomitantly with a high-fat food is approximately 80% of that given on an empty stomach. The effect of a low to medium fat diet on the bioavailability of this product is unknown.

The mean time to peak plasma concentration of the prodrug after a single fasting oral dose of 10, 12, and 16 mg of amlotinib capsules in 19 subjects with solid tumors was approximately 6 to 11 h; the mean elimination half-life was 95 to 116 h. In the dose range of 10 to 16 mg, the in vivo exposure level of amlotinib was positively correlated with the administered dose, but the linear relationship was uncertain. No significant gender differences were observed.After 15 subjects with solid tumors were dosed with a 12-mg dose once daily for 2 consecutive weeks with a 1-week discontinuation for one dosing cycle, plasma drug concentrations of the prodrug in the subjects peaked after the 14th day of consecutive dosing. After the first cycle of 14-day dosing, the plasma concentration of amlotinib reached 21.1-121 ng/mL, and decreased to 5.05-28.5 ng/mL after one week of discontinuation; after the second cycle of 14-day dosing, the plasma concentration of amlotinib reached 22.1-101 ng/mL. after 2 cycles of dosing, no significant changes were observed in plasma concentrations of the pro-drugs in the second cycle and the first cycle. concentrations did not show significant changes.

Distribution

After a single fasting oral dose of 12 mg and 16 mg of amlotinib hydrochloride capsules in subjects with advanced tumors, the mean apparent volume of distribution

ranged from 2061 to 3312 L. Human plasma protein binding of amlotinib was 93% as measured by equilibrium dialysis (in vitro), with no concentration dependence in the range of 300 to 1200 ng/mL.

Metabolism

Anrotinib is metabolized primarily by CYP1A2 and CYP3A4/5, and to a lesser extent by CYP2B6, CYP2C8, CYP2C9, CYP2C19, and CYP2D6; anrotinib is not a substrate for P-glycoprotein.

Excretion

In a 14C-labeled human trial of substance balance of amlotinib, cumulative excretion of amlotinib and its major metabolites via feces and urine was detected to be approximately 62.04% of the administered dose after a single oral dose of 12 mg amlotinib hydrochloride capsules to subjects with tumors for 2648 hours (110 days), with fecal excretion being 48.52% of the administered dose and urinary excretion being The excretion through feces is 48.52% of the dose, and the excretion through urine is 13.52% of the dose.

Special Populations

Pharmacokinetic studies have not been performed in special populations, such as those with hepatic or renal insufficiency.

2.1.4 Safety and Tolerability of Anrotinib Hydrochloride with Two Stopping Points

Adverse reaction data were summarized for a total of 1,788 subjects with advanced tumors from 22 clinical trials that have been conducted with amlotinib, covering subjects with non-small cell lung cancer, soft tissue sarcoma, small cell lung cancer, clear cell carcinoma of the kidney, colorectal cancer, medullary carcinoma of the thyroid, squamous carcinoma of the esophagus, hepatocellular carcinoma, neuroendocrine tumors, gastric cancer, and bone tumors. These subjects

were started on an oral dose of 12 mg of amlotinib for 2 weeks and discontinued for 1 week. Adverse reactions with an incidence of ≥10% included hypertension, fatigue, hand-foot syndrome, hypertriglyceridemia, proteinuria, diarrhea, decreased appetite, elevated blood thyroid-stimulating hormone, hypercholesterolemia, and hypothyroidism.

Summary Table of Adverse Reactions

The following table summarizes these 22 clinical trials of Anrotinib, observing the occurrence of adverse reactions in 1788 subjects, and is listed by system-organ classification and frequency of occurrence. The frequency of occurrence is categorized as very common (≥10%), common (1% ~10%, including 1%), occasional (0.1% ~1%, including 0.1%), rare (0.01% ~0.1%, including 0.01%), and very rare (<0.01%). Within each frequency of occurrence subgroup, these adverse reactions are listed in descending order of severity.

**Systemic Disease**	
Very common	Fatigue, weight loss
Common	Chest pain, fever, flu-like reaction [a], edema [b], cancer pain
Occasionally	Hypersensitivity reaction[c], chills
Rare	Poor wound healing[d]
**Cardiovascular disease**	
Very common	Hypertension, sinus tachycardia
Common	Sinus bradycardia, palpitations, myocardial ischemia, sinus arrhythmia
Occasionally	Heart failure, vena cava thrombosis, atrial fibrillation, pulmonary artery thrombosis, myocardial infarction, venous thrombosis of the extremities, flushing
Rare	hot flashes
**Bleeding**	

Common	Hemoptysis[e], GI bleeding[f], other bleeding[g]
**Gastrointestinal disorders**	
Very common	Diarrhea, abdominal pain, oropharyngeal pain, vomiting, nausea, toothache, oral mucositis
Common	Bloating, constipation, mouth ulcers, dry mouth, mouth pain, gastroesophageal reflux disease, intestinal obstruction
Occasionally	Gastritis, pancreatitis, enteritis, black stools
**Skin and subcutaneous tissue diseases**	
Very common	Hand-foot syndrome[h]
Common	Rash, alopecia, pruritus, exfoliation, subungual bruising, excessive sweating
Occasionally	Skin pain, acne-like dermatitis, pigmentation disorders, dry skin, erythema, pustules, seborrheic dermatitis
Rare	Eczema, dermatitis herpetiformis, generalized erythema, herpes-like dermatitis, herpes simplex, blisters
**Kidney and urinary tract disorders**	
Very common	Proteinuria
Common	Urinary tract infection
**Metabolic and Nutritional Diseases**	
Very common	Hypertriglyceridemia, decreased appetite, hypercholesterolemia, hyperglycemia, low sodium emia, hypoalbuminemia
Common	Hypokalemia, hypophosphatemia, hypocalcemia, hyperuricemia, hypomagnesemia
**Respiratory, thoracic and mediastinal disorders**	
Very common	Dysphonia, cough
Common	Dyspnea, upper respiratory tract infection, epistaxis, lung infection, pneumothorax, pleural effusion
Occasionally	Interstitial lung disease
**Blood and lymphatic system diseases**	

Very common	Decreased white blood cell count, decreased platelet count, anemia, decreased neutrophil count
Common	Decreased lymphocyte count
Rare	Increased eosinophil count
**Musculoskeletal and connective tissue disorders**	
Very common	Musculoskeletal pain[i]
Common	Joint pain
**Endocrine diseases**	
Very common	Hypothyroidism
Common	Hyperthyroidism
**Mental and Nervous System Disorders**	
Common	Headache, dizziness, insomnia, hyperalgesia
Occasionally	Abnormal sensations, drowsiness, vertigo, oral hyperalgesia, epilepsy[j], taste disorders
**Eye disorders**	
Occasionally	Blurred vision, dry eyes
**Hepatobiliary system diseases**	
Common	Hyperbilirubinemia
Occasionally	Cholecystitis, Jaundice
Rare	Liver Failure
**Ear and Labyrinthine Disorders**	
Common	Tinnitus
**Various tests**	
Very common	Elevated blood thyrotropin, elevated aspartate aminotransferase, elevated gamma-glutamyltransferase, elevated blood bilirubin, elevated alanine aminotransferase, prolonged QT interval on ECG, elevated low-density lipoproteins, positive erythrocytes in the urine, elevated alkaline phosphatase in the blood, elevated bilirubin conjugated, positive occult blood

Common	Elevated lipase, elevated amylase, elevated creatinine, prolonged activated partial thromboplastin time, elevated blood urea

a Influenza-like reactions are characterized by chills, nasal congestion, runny nose, muscle aches, and malaise;

b Edema includes peripheral edema, facial edema, localized edema, and generalized edema;

c Hypersensitivity reactions include systemic anaphylactic reactions, upper respiratory tract hypersensitivity reactions, and acute anaphylactic reactions;

d Clinical trials excluded subjects with unhealed wounds;

e Hemoptysis includes bleeding events in the lungs and upper respiratory tract such as hemoptysis and bronchial hemorrhage;

f Bleeding from the digestive tract includes bleeding from the gums, bleeding from the mouth, pharyngeal congestion, gastric bleeding, intestinal bleeding, anal bleeding, and hemorrhoidal bleeding;

g Other bleeding includes tumor bleeding, nail bed bleeding, wound bleeding, subcutaneous bleeding, vaginal bleeding, menstrual bleeding, uterine bleeding, retinal bleeding, conjunctival bleeding, and cerebral bleeding;

h Hand-foot syndrome is termed palmoplantar erythema syndrome in MedDRA;

i Musculoskeletal pain includes back pain, limb pain, myalgia, skeletal muscle pain, neck pain, bone pain, thoracic musculoskeletal pain, and low back and rib pain;

j Epilepsy including partial seizures.

Anrotinib hydrochloride is being studied in China in combination with TQB2450 Injection (PD-L1) for the treatment of advanced soft-tissue sarcoma, small-cell lung cancer, cholangiocarcinoma, hepatocellular carcinoma, triple-negative breast cancer, non-small-cell lung cancer, and melanoma in a number of Phase Ib, and in combination with AK105 Injection for the first-line treatment of unresectable hepatocellular carcinoma, in a number of Phase Ib/II studies with a manageable and tolerable safety profile, and adverse events were consistent with the data summarized above. summarized data, with no unintended adverse events due to the combination.

Anrotinib hydrochloride is a multi-targeted receptor tyrosine kinase inhibitor that ①has significant inhibitory activity against angiogenesis-related kinases, such as VEGFR, FGFR, PDGFR, and c-Kit kinase, a kinase associated with tumor cell

proliferation. Inhibition of angiogenesis kinases has a broader spectrum of inhibition (e.g., against Met, FGFR1/2/3). ② It also has significant inhibitory activity against some of the kinase targets under investigation, such as Aurora-B, c-FMS, DDR1, and so on. ③ It has significant inhibitory activity against a variety of kinase mutants, such as PDGFRα, cKit, Met, EGFR, etc., and the inhibitory activity against mutants is even stronger than that against wild type.

Anrotinib hydrochloride capsules are hard capsules. The structural formula of the main ingredient, Anrotinib, is shown below:

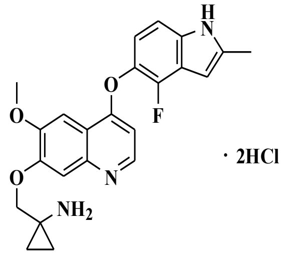

Molecular Formula: C23H22FN3O3-2HCl Molecular Weight: 480.36

Chia Tai Tianqing Pharmaceutical Group Co., Ltd. developed and declared Anrotinib hydrochloride capsule with independent intellectual property rights.In March 2011, we obtained the clinical research approval document from the State Food and Drug Administration (SFDA: 2011L00661), and permitted to carry out the clinical research.Since March 2016, under the leadership of Shanghai Jiaotong University Affiliated Chest Hospital, we have completed a non-small cell lung cancer Phase III preliminary efficacy and safety study, 439 patients were enrolled and randomized into the treatment group and placebo group according to 2∶1, and the treatment group was orally administered with amlotinib 12 mg /d. The study found that the PFS of the treatment group and the placebo group were 5.37 months and 1.4 months, and the OS was 9.63 months and 6.3 months, respectively, and the objective remission rate, disease control rate of the amlotinib group were 9.18% and 80.95%, which were significantly better than 0.7% and 37.06% in the placebo group. Adverse effects in the androtinib group mainly included hypertension, proteinuria,

and hand-foot syndrome, but they were mild or moderate and manageable. The results showed that the efficacy and safety of erlotinib in patients with advanced non-squamous NSCLC were high.

As the first vascular-targeted drug effective in the single-agent treatment of advanced NSCLC, the targets of amlotinib include VEGFR1/2/3, FGFR1/2/3, and PDGFRα/β, which is more comprehensive in inhibiting neovascularization than bevacizumab, and it can avoid the resistance caused by the signaling pathway complementarity, and achieve the effect of efficiently inhibiting the neovascularization, 2.1.5 Amlotinib hydrochloride has been shown to be highly effective and safe in patients with advanced nonsquamous NSCLC, but with mild or controlled symptoms.

2.1.5 Phase II Clinical Trial of Anrotinib Hydrochloride for Non-Small Cell Lung Cancer

From August 2013, 13 research centers across China, using a multicenter, randomized, double-blind, placebo 1:1 controlled design, observed 117 cases of pathologically confirmed advanced (Stage IIIB/IV) non-small cell lung cancer, non-small cell lung cancer patients who had received treatment in the third line or above or who were unable to tolerate treatment, and the main efficacy index was progression-free survival.

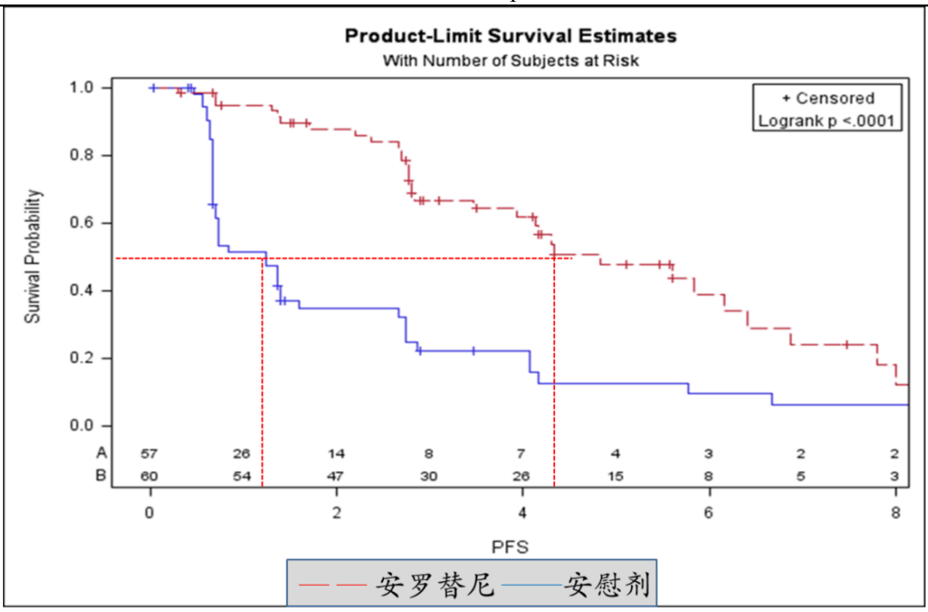

The upper panel shows the progression-free survival (PFS, months) after treatment in the two groups, with a median PFS of 4.83 months (95% CI 3.47-6.40) and 1.23 months (95% CI 0.70-1.60) in the amlotinib group (60 patients) and the placebo group (57 patients), respectively, (HR=0.320 95% CI 0.200-0.511 p&lt ;.0001).

Objective remission rates in both groups were 10.00% in the anrotinib group and 0 in the placebo group (p < .0001).

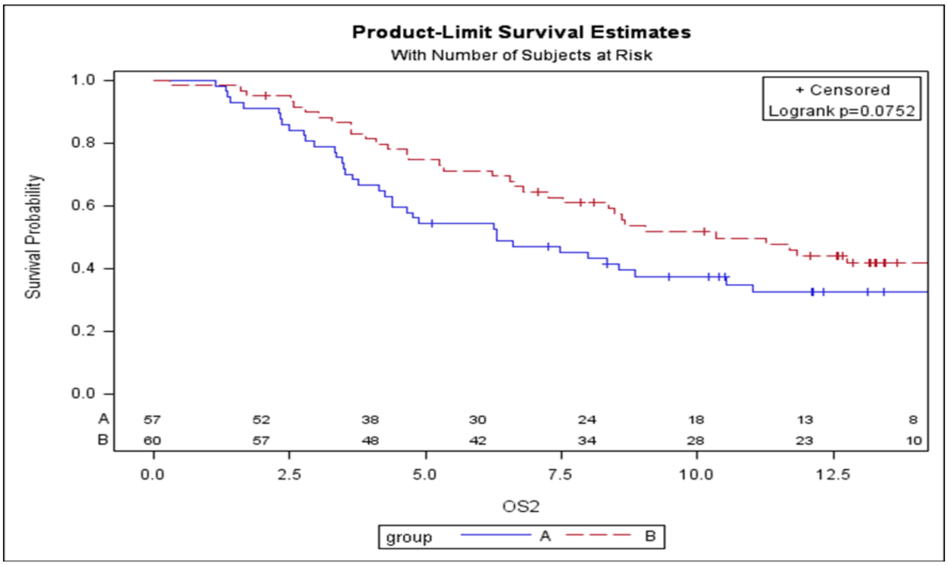

As of February 2, 2016, the median overall survival (OS, months) of patients in the two groups was 9.3 months (95% CI: 6.8-15.1) and 6.30 months (95% CI: 4.3-10.5) for the anrotinib group and placebo group, respectively, (HR = 0.78; 95% CI: 0.51-1.18 p= 0.2316).

The incidence of adverse events was 91.67% and 70.18% in the anrotinib and

placebo groups, respectively (p=0.004). AEs that occurred at a higher rate in the anrotinib group than in the placebo group were: hypertension, elevated TSH , hand-foot syndrome, elevated thyroglobulin, elevated total cholesterol, and diarrhea, with the most common treatment-related grade 3-4 AEs in the anrotinib group being hypertension (10%), elevated thyroglobulin (5%), and hand-foot syndrome (3.33%). All AEs resolved after dose adjustment or symptomatic management. No treatment-related lethal events occurred in the study. This phase II clinical study thus demonstrates the efficacy and safety of anilotinib monotherapy for the third-line or higher treatment of advanced NSCLC.

2.1.6 Phase III Clinical Trial of Anrotinib Hydrochloride for Non-Small Cell Lung Cancer

From April 2016, 31 research centers nationwide, using a multicenter, randomized, double-blind, placebo 2:1 controlled design, observed 437 patients with pathologically confirmed advanced (Stage IIIB/IV) non-small cell lung cancer who had received treatment for third-line or above or who were unable to tolerate the treatment, and the main efficacy index was overall survival.

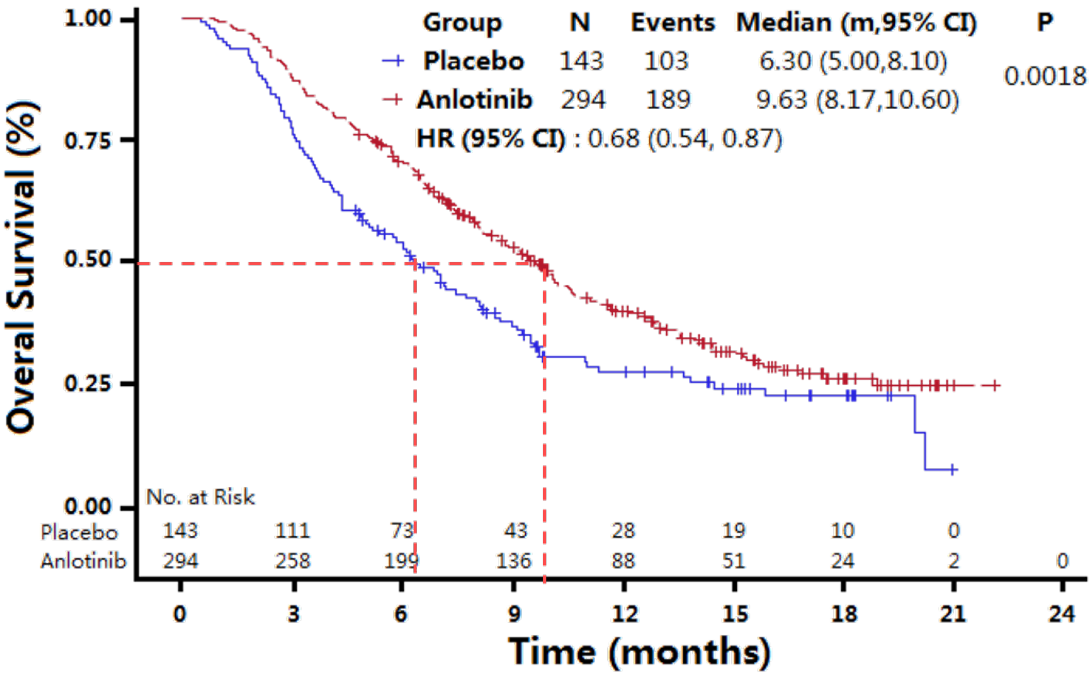

The graph above shows the overall survival (OS, months) after treatment in the two groups, with a median OS of 9.63 months (95% CI 8.17-10.60) and 6.3 months (95% CI 5.00-8.10) in the amlotinib group (294 patients) and the placebo group (143

patients), respectively, (HR=0.68 95% CI 0.54-0.87 p=0.0018) .

The objective remission rate in both groups was 9.18% in the anrotinib group and 0.7% in the placebo group (p < .0001).

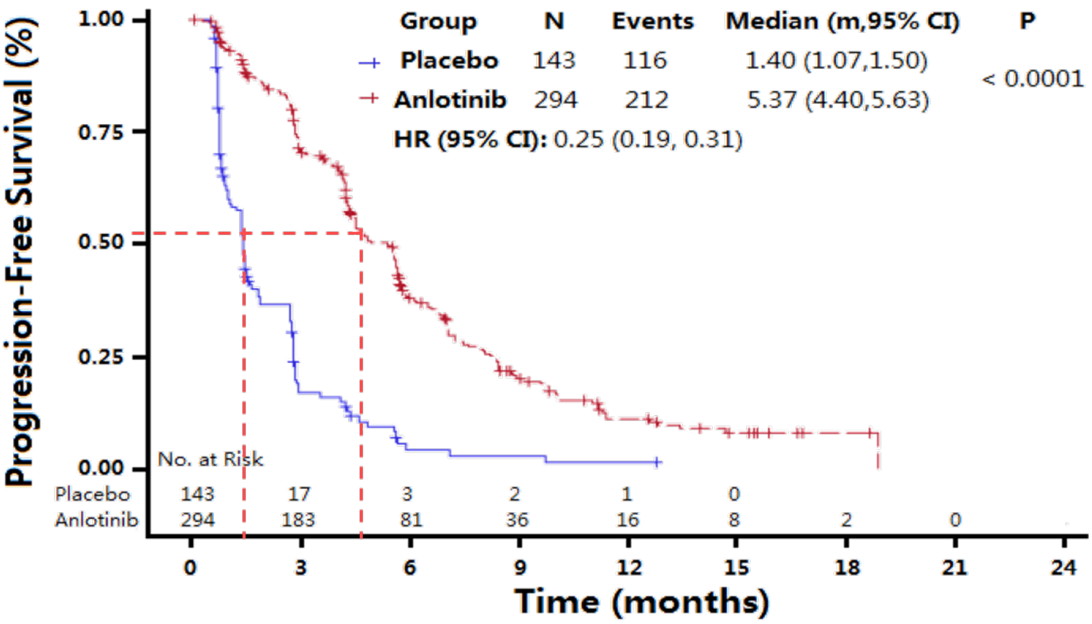

The upper panel shows the disease progression free period (PFS, months) after treatment for both groups, with a median PFS of 5.37 months (95% CI: 4.40-5.63) and 1.40 months (95% CI: 1.07-1.50) for the anrotinib group and the placebo group, respectively, (HR=0.25 95% CI: 0.19-0.31 p<0.0001).

The anrotinib group was also significantly better than the control group in both secondary endpoints of ORR (9.2% vs. 0.7%, p<0.0001) and DCR (81.0% vs. 37.1%, p<0.0001). In addition, anrotinib demonstrated a favorable safety profile, with an adverse event rate similar to that of the control group. During the course of the study, dose reductions in the anrotinib group were attributed to hand and foot skin reactions, hypertension, oral mucositis, diarrhea, anorexia, hepatic impairment, hypertriglyceridemia, proteinuria, and malaise. Application for dose reduction occurred mostly after 2 cycles of Anrotinib capsules, and most patients experienced relief of toxic reactions after taking the reduced dose of the test drug.

This study confirms that the efficacy of Anrotinib hydrochloride in patients with advanced non-small cell lung cancer is exact, providing a new treatment for patients with refractory advanced non-small cell lung cancer after failure of multiline chemotherapy and EGFR-TKI resistance. The safety findings of Anrotinib hydrochloride showed no other unintended adverse reactions compared with other similar anti-angiogenic targeted drugs already on the market, and its adverse

reactions could be controlled by dose adjustment, suspension of administration and symptomatic treatment, demonstrating the favorable safety profile of Anrotinib hydrochloride. Anrotinib hydrochloride can provide survival benefit for patients with advanced non-small cell lung cancer with a controlled safety profile.

2.2 Penpulimab

Penpulimab is a monoclonal antibody (mAb) to human immunoglobulin G1 (IgG1) that acts directly against human programmed cell death-1 (PD-1). penpulimab effectively prevents human PD-1 from interacting with its ligand, programmed cell death protein-1-ligand. Penpulimab effectively prevents binding of human PD-1 to its ligands programmed cell death-1 ligand 1 (PD-L1) and programmed cell death-1 ligand 2 (PD-L2).

Penpulimab has a typical antibody structure consisting of 2 heavy chains of the IgG1 isoform and 2 light chains of the κ isoform covalently linked to each other by disulfide bonds. The Penpulimab antibody was bioengineered to specifically carry a crystallizable fragment (Fc).

The amino acid mutation of the crystallizable (Fc) fragment carried by Penpulimab antibody effectively removes the ability to bind to the Fcγ receptor I, thus avoiding immune cell damage due to the ADCC effect and the possible weakening of the anti-tumor effect of PD-1 antibody ([) (1) (3) (]).

Penpulimab is expressed in a Chinese hamster ovary cell line with a total molecular weight, including oligosaccharides, of approximately 150 kDa. Each heavy chain consists of 448 amino acids and has a molecular weight of 48,923.56 Da (theoretically excluding glycosylation). Each light chain consists of 214 amino acids and has a molecular weight of 23,598.38 Da.

Penpulimab, known in Chinese as paianpulizumab, has been approved for marketing by the State Drug Administration in August 2021 for adult patients with relapsed or refractory classical Hodgkin's lymphoma who have undergone at least second-line systemic chemotherapy. Penpulizumab is the only anti-PD-1 monoclonal antibody with IgG1 subtype in China, meanwhile, it is potent and safer after structural modification and optimization.

2.2.1 Preclinical study results of Penpulimab

2.2.1.1 Summary of pharmacokinetics

The pharmacokinetic (PK) profile of Penpulimab was investigated in crab-eating monkeys. After Penpulimab was administered at doses of 1, 3, and 10 mg/kg, clearances (CL) of 0.33 and 0.32 mL/h/kg, 0.32 and 0.27 mL/h/kg, and 0.25 and 0.20 mL/h/kg were observed in males and females, respectively. In male and female animals, the volume of distribution of Penpulimab at 1, 3, and 10 mg/kg doses was 57.40 mL/kg and 63.69 mL/kg, 54.73 mL/kg and 62.54 mL/kg, and 22.04 mL/kg and 44.01 mL/kg, respectively.The half-life of Penpulimab at doses of 1, 3, and 10 mg/kg in males and females (t1/2) were 122.80h and 140.31h, 121.65h and 166.94h, 64.03h and 167.16h, respectively.

The mean values of Cmax ratio of Penpulimab in crab-eating monkeys were 1:2.94:13.03 and 1:2.90:12.01 for males and females, respectively, after intravenous administration of 1, 3, and 10 mg/kg doses.The mean values of AUC0-t ratio of Penpulimab in males and females Penpulimab were 1:3.14:14.63 and 1:4.07: respectively.These results demonstrated that the PK of Penpulimab was linear over the 1-10 mg/kg dose range. 19.44.These results demonstrated that the PK of Penpulimab was linear in the dose range of 1-10 mg/kg. There was no significant difference in PK parameters of Penpulimab between female and male monkeys.

Toxicokinetic parameters of Penpulimab were evaluated in repeated-dose toxicology studies in crab-eating monkeys after intravenous infusion of Penpulimab at doses of 4, 15, and 60 mg/kg every 2 weeks for 6 weeks (3 administrations). At doses of 4, 15, and 60 mg/kg, the AUC0-336h ratios of the last dose of Penpulimab to the first dose were 0.55, 0.70, and 1.35 in males and 0.05, 1.07, and 1.03 in females, respectively, and there was no significant accumulation in the animals; however, after repeated administration of Penpulimab to 4 mg/kg females, systemic Exposure was significantly reduced in 4 mg/kg females. These results suggest that systemic exposure to Penpulimab (in females in the 4 mg/kg dose group) may be reduced by the development of anti-drug antibodies (ADA) or that the bioassay results were interfered with by ADA.

2.2.1.2, Penpulimab Preclinical Pharmacology

PD-1 is a member of the superfamily of Ig molecules involved in the regulation of T-cell activation, a receptor found in T cells, B cells, macrophages, NK cells, dendritic cells, and mast cells.PD-1 is involved in the inhibitory activation of T cells, as well as the production of cytokines associated with effector T-cell function, proliferation, cell survival, and downstream effects of transcription factors.

Penpulimab exhibits the following activities as a PD-1 monoclonal antibody:

- Penpulimab was able to bind to human PD-1 with high affinity, in which the rate of dissociation of Penpulimab from PD-1 was significantly reduced, suggesting a more robust binding of Penpulimab to the target antigen.
- Penpulimab can effectively block the interaction between human PD-1/PD-L1 and PD-1/PD-L2.
- Penpulimab significantly increased IL-2 and IFN-γ secretion by human primary T cells co-cultured with PD-L1-expressing cells, such as dendritic cells.
- Penpulimab significantly inhibited tumor growth in the SCID/Beige mouse Raji-PD-L1 cell transplantation tumor model, in which Raji-PD-L1 was co-injected with human peripheral blood mononuclear cells (PBMCs) into immunocompromised mice to test the inhibitory effect of Penpulimab on the growth of inoculated tumor cells.
- Penpulimab inhibits the growth of mouse colorectal cancer tumors in a PD-1 knock-in mouse mouse colorectal cancer cell (MC38 cell) transplantation tumor model.

2.2.1.3. Penpulimab Preclinical Toxicology

Crab-eating monkeys are considered to be a relevant animal species suitable for use in non-clinical safety studies of Penpulimab. The safety of Penpulimab was explored in a 6-week repetitive dose toxicity study conducted in Crab-Eating Monkeys in accordance with the Good Laboratory Practice (GLP) for non-clinical drug studies, where the maximum dose tested was 60 mg/kg/2 weeks.

Penpulimab doses up to 60 mg/kg were well tolerated when administered intravenously. Treatment-related histopathologic changes in the kidneys (increased renal cortical tubular pigmented or pigmented interstitial macrophages) were

observed in some animals, but had no effect on renal function. No specific effects on vital functions of the cardiovascular system, central nervous system and respiratory system were observed. A toxic response dose level (NOAEL) of 60 mg/kg was not observed under study conditions.

2.2.2 Penpulimab Clinical Studies

The Phase Ia dose-escalation trial of Penpulimab-101 in Australia enrolled subjects with advanced solid tumors who had failed standard of care and evaluated three dose levels (1 mg/kg, 3 mg/kg, and 10 mg/kg Q2W) in a classic "3+3" design. As of February 12, 2019, a total of 16 subjects enrolled in the 1 mg/kg (n=3), 3 mg/kg (n=6), and 10 mg/kg (n=7) dose groups who had failed multiple treatments in advanced stages had completed enrollment, and 14 of these subjects had completed at least one post-administration tumor imaging assessment, with preliminary results showing an ORR of 29%, a DCR of 57%, and a remission that lasted for a long time and remained The Phase Ib extension study enrolled 18 subjects with colorectal cancer (n=1), gastric cancer or gastroesophageal adenocarcinoma (n=8), hepatocellular carcinoma (n=4), and esophageal squamous carcinoma (n=5) treated with Penpulimab at a fixed dose of 200mg Q2W, of which 9 subjects in advanced disease who had failed multiple treatment regimens had at least one post-administration tumor imaging assessment, an ORR of 22%, and a DCR of 67%. ORR 22%, DCR 67%. 34 subjects had a median of 5 administrations and a maximum of 29 administrations of Penpulimab, of which 14 subjects (41%) had a drug-related adverse event (TRAE), 4 (12%) had a drug-related Grade 3 adverse event, no Grade 4 adverse events were reported, no DLT events occurred, 3 (9%) were suspended due to an AE, and no AEs led to discontinuation of the drug. The most common TRAEs (>1%) were The most common TRAEs (>5%) were hyperthyroidism (9%), hypothyroidism (6%), fatigue (6%), and rash (6%). No drug-associated colitis or pneumonia of any grade was reported.

Results from the Phase III registry study of pembrolizumab in combination with chemotherapy for the first-line treatment of advanced squamous NSCLC have also been presented at the 2021 China Clinical Oncology Annual Meeting, which showed that pembrolizumab in combination with chemotherapy significantly prolonged progression-free survival (7.0 months vs. 4.2 months) and significantly

reduced the risk of disease progression or death by up to 60% compared to chemotherapy-treated groups. The incidence of grade ≥3 immune-related adverse events was only 2.9%. Based on the results of this study, the marketing application for pembrolizumab in combination with chemotherapy for the first-line treatment of advanced squamous NSCLC has also been submitted to the State Drug Administration in 2021, and will hopefully be approved for marketing in 2022.

3. Purpose of the study

- To evaluate the efficacy and safety of a Penpulimab-based combination regimen for the neoadjuvant/adjuvant treatment of resectable locally advanced NSCLC.

3.1 Main Objectives

- To observe the primary efficacy of the Penpulimab-based combination regimen neoadjuvant/adjuvant treatment of resectable locally advanced NSCLC.

3.2 Secondary objective

- To observe the safety of Penpulimab-based combination regimen for neoadjuvant/adjuvant treatment of resectable locally advanced NSCLC.

4 Trial design

4.1 Overall design

4.1.1 Overview of the study design

This study evaluates the efficacy and safety of the Penpulimab-based combination regimen neoadjuvant/adjuvant for the treatment of resectable locally advanced NSCLC, as shown in Figure 1.

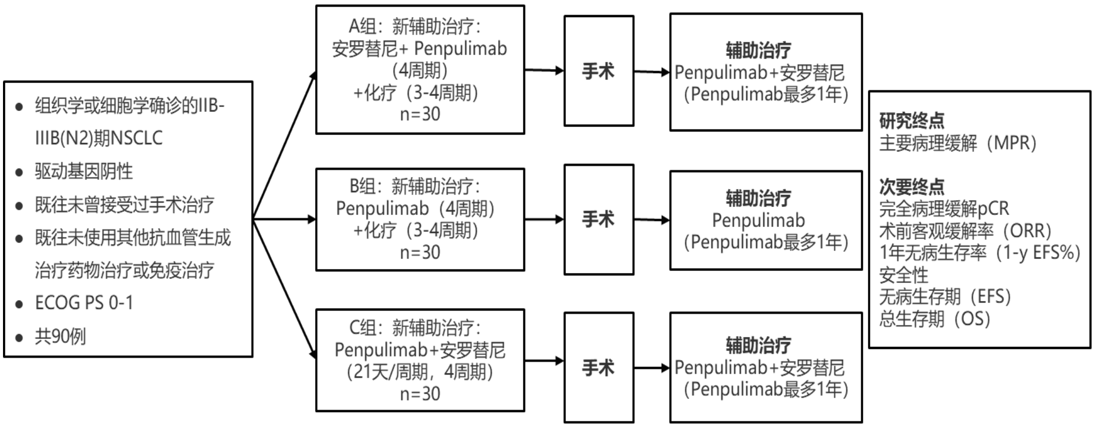

Figure 1. study design

This study is a multicenter, randomized, open-label trial planned to be conducted in Tianjin Cancer Hospital and other hospitals. Ninety patients are expected to be enrolled and randomly assigned to groups A/B/C with 30 patients in each group. Initiation is expected to start from March 2021, with an approximate end of enrollment in June 2022.

Study treatment until intolerable toxicity, confirmed disease progression according to RECIST v1.1, death, loss to follow-up, termination of the study by the sponsor, withdrawal of informed consent by the subject, or fulfillment of other termination criteria in the protocol, whichever occurs first.

4.1.2 Sample Size Setting

Assuming that the MPR for each arm of this study is 42%, and taking the historical data (the MPR for atalizumab monotherapy in LCMC3 is 19%), taking α=0.05, β=0.2, and using the PASS15 software, and after a preset dropout rate of 10%, it is proposed to enroll 30 subjects in each arm of the study, with a total of 90 subjects enrolled in the study.

4.1.3 Randomization method

The randomized envelope method of grouping was used in this study. Each patient will correspond to a random serial number after enrollment. Each random serial number will be divided into groups A, B and C according to certain rules.

4.1.4 Drug administration program

Group A (n=30):

Neoadjuvant therapy (amlotinib+ Penpulimab, 4 cycles; chemotherapy 3-4 cycles): amlotinib, 12 mg, po, qd, continuous oral for 2 weeks with one week off, 3 weeks for 1 cycle; Penpulimab, 200 mg, iv, d1, 3 weeks for 1 cycle; chemotherapy (3-4 cycles): non-squamous cancer patients chose pemetrexed+carboplatin/cisplatin (Pemetrexed Pemetrexed,500mg/m$^{(2)}$，iv,d1,3 weeks for one cycle; cisplatin,75mg/m$^{(2)}$，iv,d1,3 weeks for one cycle; carboplatin AUC 5,iv,d1,3 weeks for one cycle), patients with squamous carcinoma choose paclitaxel+carboplatin/cisplatin (paclitaxel,175mg/m$^{(2)}$，iv,d1,3 weeks for one cycle; cisplatin,75mg/m$^{(2)}$，iv,d1,3 weeks as a cycle; carboplatin AUC 5,iv,d1,3 weeks as a cycle) (carboplatin dose (mg): set AUC 5mg/ml/min× [urinary creatinine clearance (ml/min)+25],AUC:area under the curve).

Surgery was performed 3-7 weeks after the final dose of amlotinib, and was evaluated by the investigator for surgery

Adjuvant therapy 4-6 weeks postoperatively as assessed by the investigator

Adjuvant therapy: anilotinib, 12 mg, po, qd, 2 consecutive oral weeks off for one week, 1 cycle of 3 weeks (until PD); Penpulimab, 200 mg, iv, d1, 1 cycle of 3 weeks (until PD or up to 1 year of treatment);

Group B (n=30):

Neoadjuvant therapy (Penpulimab, 4 cycles; chemotherapy 3-4 cycles): penpulimab, 200 mg, iv,d1, 1 cycle in 3 weeks; chemotherapy (3-4 cycles): pemetrexed+ Carboplatin/cisplatin for patients with non-squamous carcinoma (pemetrexed,500 mg/m$^{(2)}$，iv,d1, 1 cycle in 3 weeks; cisplatin,75 mg/m^{2}，iv,d1,3 weeks for one cycle; carboplatin AUC 5,iv,d1,3 weeks for one cycle), and patients with squamous carcinoma chose paclitaxel+ Carboplatin/cisplatin, (paclitaxel, 175mg/m$^{(2)}$，iv,d1,3 weeks for one cycle; cisplatin, 75mg/m$^{(2)}$，iv,d1,3 weeks for one cycle; carboplatin AUC 5,iv,d1,3 weeks for one cycle) (Carboplatin dose (mg): set AUC 5mg/ml/min× [urinary creatinine clearance (ml/min)+25],AUC:area under the

curve).

Surgery 3-7 weeks after final Penpulimab administration, as assessed by the investigator

Adjuvant therapy 4-6 weeks postoperatively as assessed by the investigator

Adjuvant therapy: Penpulimab, 200 mg, iv, d1, 1 cycle of 3 weeks (until PD or up to 1 year of treatment);

Group C (n=30):

Neoadjuvant therapy (amlotinib+ Penpulimab, 4 cycles): amlotinib, 12 mg, po, qd, 2 consecutive oral weeks off for one week, 3 weeks for 1 cycle; Penpulimab, 200 mg, iv, d1, 3 weeks for 1 cycle;

Surgery 3-7 weeks after final dose of amlotinib, as evaluated by the investigator

Adjuvant therapy 4-6 weeks postoperatively as assessed by the investigator

Adjuvant therapy: amlotinib, 12 mg, po, qd, 2 consecutive oral weeks off for one week, 1 cycle in 3 weeks (until PD); Penpulimab, 200 mg, iv, d1, 1 cycle in 3 weeks (until PD or up to 1 year of treatment);

All chemotherapeutic agents (carboplatin, cisplatin, pemetrexed, and paclitaxel) are required to be nationally consistently evaluated drugs.

Patients who experienced complete remission (CR), partial remission (PR), and stable disease (SD) continued dosing until disease progression, intolerable toxicity, or the patient requested discontinuation. Dosing is discontinued in patients with progressive disease (PD).

There are no results from clinical studies in special populations including the elderly, children, pregnant women, or patients with hepatic or renal insufficiency. Analysis of blood concentration data in patients of different ages and genders has shown that patients' blood concentrations are not affected by factors such as age and gender; therefore, dosage adjustments based on age and gender are not recommended.

4.1.5 Dosing cycle

Take 1 capsule (12 mg) of amlotinib hydrochloride on an empty stomach once

daily before breakfast. Take 2 consecutive oral doses for 2 weeks and stop for 1 week, i.e. 3 weeks (21 days) as a treatment cycle. In the event of a missed dose, confirm that the time to the next dose is shorter than 12 hours, there will be no make-up dose.

Penpulimab, 200 mg, IV, day 1, 3 weeks (21 days) for one treatment cycle and four cycles of neoadjuvant therapy, up to 1 year of adjuvant therapy.

Chemotherapy (3-4 cycles): pemetrexed, 500 mg/m$^{(2)}$ ·iv,d1, one cycle in 3 weeks; cisplatin, 75 mg/m$^{(2)}$ ·iv,d1, one cycle in 3 weeks; carboplatin AUC 5,iv,d1, one cycle in 3 weeks; paclitaxel, 175 mg/m$^{(2)}$ ·iv,d1, one cycle in 3 weeks.

In subjects with disease control (CR+PR+SD) and tolerable adverse effects, amlotinib was continued until disease progression occurred, the subject voluntarily withdrew informed consent, and intolerable toxicity occurred. Dosing was terminated when the subject was deemed unsuitable by the investigator for continued dosing or was evaluated as PD according to RECIST 1.1 criteria.

4.2 Efficacy Evaluation and Analysis

During the neoadjuvant treatment phase, imaging assessments were performed once on Day 21 of Cycle 2 and once within 7 days prior to surgery. Postoperative MPR evaluation was performed; postoperative adjuvant treatment phase was evaluated every 12 weeks (up to 1 year postoperatively) and every 24 weeks starting in the second year (up to 5 years postoperatively); all subjects were required to keep all imaging data.

The expected duration of study treatment for each subject will continue until imaging-confirmed tumor progression, provided that none of the following conditions occur, such as withdrawal of informed consent by the subject, drug toxicities that are not tolerable, or the investigator's opinion that further testing is not appropriate.

4.3 Biomarker analysis

PD-L1 expression will be measured once during the screening period and once

after surgery, and data from the different PD-L1 expression groups will be analyzed to compare the efficacy of the treatment.PD-L1 will be detected by immunohistochemistry using the IHC 22C3 PharmDx (DAKO) assay.PD-L1 will be detected by immunohistochemistry using the IHC 22C3 PharmDx (DAKO) assay.

4.4 Survival Follow-up

Subjects entered the follow-up period after discontinuing trial administration; subjects remained in the trial period and should be followed up further until death or loss to follow-up. All subjects will be followed up 5 years after surgery at a frequency of every 48 weeks. Follow-up can be done by telephone by asking the subject himself/herself, his/her family or local physician.

4.5 Follow-up of Adverse Events

Adverse events that have not recovered by the time the study drug is discontinued should be followed up and a final evaluation made. All patients should be followed for a 21-day safety visit after final dosing to detect any new adverse events.

5. Subject selection and withdrawal

5.1 Inclusion Criteria

1. Subjects voluntarily enrolled in the study, signed the informed consent form, were compliant and cooperated with the follow-up visits
2. Age at the time of signing the informed consent≥ 18 years and≤ 70 years, male or female;
3. Eastern Cooperative Oncology Group (ECOG) physical status score of 0 or 1;
4. Expected survival of not less than 12 weeks;
5. Male and female patients of childbearing age agree to use a reliable method of contraception before entering the trial, during the study and until 8 weeks after discontinuation.
6. Consent to collect tumor histology specimens needed for the study and apply

them to the study;

7. Patients who agree to undergo radical surgical treatment;
8. Patients who, in the judgment of the specialist, have no contraindications to surgery;
9. Patients with non-small cell lung cancer diagnosed by pathologic histology or cytology (according to the WHO 2015 classification); and patients with radically resectable stage IIB-IIIB (N2) non-small cell lung cancer (as determined by the International Association for the Study of Lung Cancer (IASLC) Manual of Thoracic Tumor Staging, 8th edition); and with primary or lymph node metastasis testing clearly EGFR/ALK/ROS1 negative (patients with squamous carcinoma will have the need for genetic testing at the discretion of the investigator);
10. Subjects with primary non-small cell lung cancer not previously treated with surgery, chemotherapy, radiation therapy, and biologic therapy;
11. Patients with resectable lesions as judged by the investigator, with clinical stage IIB-IIIB (N2), who can obtain sufficient tumor histology specimens (non-cytology) for molecular marker analysis;
12. Evaluable disease with at least one measurable lesion according to the Criteria for the Evaluation of Efficacy in Solid Tumors (RECIST 1.1).
13. Subjects must have adequate lung function for the intended lung resection.
14. Normal major organ function should meet the following criteria:

(1) Pulmonary ventilation function test, FEV1≥ 1.5L, or expected FEV1≥ 800ml after lobectomy/total lung resection;

(2) Criteria for routine blood tests (no transfusion of blood or blood products within 14 days, not corrected with G-CSF and other hematopoietic stimulating factors):

a) Hemoglobin (HB) ≥90g/L
b) Absolute neutrophil count (ANC) ≥1.5× 10^9/L
c) Platelets (PLT) ≥80× 10^9/L;

(3) Biochemical tests need to meet the following indicators:

a) Total bilirubin (TBIL) ≤1.5 times the upper limit of normal (ULN);
b) Alanine aminotransferase (ALT) and aspartate aminotransferase AST≤ 2.5× ULN;
c) Serum creatinine (Cr) ≤ 1.5× ULN or creatinine clearance (CCr) ≥ 60 ml/min.

15. International normalized ratio (INR) of prothrombin time ≤ 1.5 and partial thromboplastin time (APTT) ≤ 1.5 times the upper limit of normal in patients

who have not received anticoagulation therapy. Patients receiving full or parenteral anticoagulant therapy may be admitted to clinical trials as long as the dose of anticoagulant has been stable for at least 2 weeks prior to entry into the clinical study and the results of coagulation assays are within local therapeutic limits;

16. Women of childbearing age (15～ 49 years) should agree that they must use contraception (e.g., IUDs, birth control pills, or condoms) during the study period and for 6 months after the end of the study; they must have had a negative serum or urine pregnancy test within 1 week prior to study enrollment and must be a non-breastfeeding patient; men should agree that they must use contraception during the study period and for 6 months after the end of the study period.

5.2 Exclusion Criteria

1. Large cell carcinoma and mixed cell lung cancer;
2. Patients who, in the judgment of the investigator, have a high probability of fatal hemorrhage due to tumor invasion of vital blood vessels during the subsequent study period; or the presence of significant cavitary or necrotic tumors in the lungs;
3. Any systemic anticancer therapy, including cytotoxic drug therapy, immunologic drug therapy, or experimental therapy, for NSCLC;
4. Have had localized radiotherapy for NSCLC;
5. Patients who have had a cancer other than NSCLC in the five years prior to the start of treatment in this study. Excluding cervical carcinoma in situ, cured basal cell carcinoma, and bladder epithelial tumors [including Ta and Tis];
6. Patients with prior use of amlotinib and other anti-angiogenic agents;
7. Patients with prior use of Penpulimab, or other anti-PD-1, anti-PD-L1, anti-CTLA-4 antibodies, and any other antibody or drug therapy targeting the T-cell co-stimulatory or checkpoint pathways, such as ICOS or agonists (e.g., CD40, CD137, GITR, OX40, etc.);
8. Hypersensitivity to amlotinib or Penpulimab or any component of the chemotherapeutic agent;
9. Patients with multiple factors that interfere with oral administration of medications (e.g., inability to swallow, chronic diarrhea, and intestinal

obstruction); and

10. Patients with the presence of any severe and/or uncontrolled medical condition, including:

1) Patients with poorly controlled blood pressure (systolic blood pressure ≥ 150 mmHg and diastolic blood pressure ≥ 100 mmHg);
2) Patients with class I or greater myocardial ischemia or myocardial infarction, arrhythmias (including QTc≥ 480ms) and class ≥2 congestive heart failure (New York Heart Association (NYHA) classification);
3) Abnormal coagulation (INR > 1.5 or prothrombin time (PT) > ULN + 4 seconds or APTT > 1.5 ULN), bleeding tendency or on thrombolytic or anticoagulant therapy; Note: The use of low-dose heparin for prophylactic purposes is permitted (daily dosage of 0.6 million for adults), provided that the International Normalized Ratio of the Prothrombin Time (INR) is ≤ 1.5 ~12,000 U) or low-dose aspirin (≤ 100 mg daily) for prophylactic purposes.
4) Active or uncontrolled serious infections;
5) Cirrhosis, decompensated liver disease, active hepatitis or chronic hepatitis requiring antiviral therapy;
6) Renal failure requiring hemodialysis or peritoneal dialysis;
7) History of immunodeficiency, including being HIV-positive or having other acquired, congenital immunodeficiency diseases, or a history of organ transplantation;
8) Poorly controlled diabetes mellitus (fasting blood glucose (FBG) > 10 mmol/L);
9) Those with routine urinalysis suggestive of urinary protein ≥++ and confirmed 24-hour urine protein quantification > 1.0 g;
10) Patients with seizures and requiring treatment;
11) Prolonged unhealed wounds or fractures, etc;
12) Clinically significant hemoptysis (>50 ml per day) within 2 weeks prior to enrollment; or clinically significant bleeding symptoms or a definite bleeding tendency, such as gastrointestinal bleeding, bleeding gastric ulcer, fecal occult blood++ and above at baseline, or vasculitis;

11. Pre-existing interstitial lung disease, drug-induced interstitial disease, radiation pneumonitis requiring hormonal therapy, or any clinically evidenced active interstitial lung disease;

12. Those who have had an arterial/venous thrombotic event within 6 months, such as cerebrovascular accidents (including transient ischemic attacks), deep vein thrombosis, and pulmonary embolism;
13. Presence of current peripheral neuropathy of ≥ CTCAE degree 2, except as a result of trauma;
14. Patients requiring total right lung resection; subjects who have had major surgery or severe trauma have had the effects of surgery or trauma resolved for less than 14 days prior to enrollment;
15. Patients who are participating in another clinical study or are less than 4 weeks from the end of treatment in a previous clinical study;
16. Patients with mixed small cell lung cancer components;
17. Have received a live or attenuated vaccine within 30 days prior to the first dose of Penpulimab or plan to receive a live or attenuated vaccine during the study period;
18. Known history of severe hypersensitivity reactions to other monoclonal antibodies;
19. Pregnant or lactating women;
20. Prior history of definite neurologic or psychiatric disorders, including epilepsy or dementia;
21. Patients who, in the judgment of the investigator, may have other factors that may force the mid-term termination of this study, such as other serious illnesses or serious laboratory test abnormalities or concomitant other factors that would compromise the safety of the subject, or the family or community in which the trial data and samples were collected.

5.3 Exclusion Criteria

1. Failure to administer the drug at the dose, method and regimen specified in this study protocol (discontinuation of the drug for a cumulative period of more than four weeks in one dosing cycle is recorded as a dropout);
2. Those who were treated with other chemotherapy or experimental drugs other than this protocol during the trial;
3. Those who did not meet the criteria and were included in error;
4. Patients who are not on medication.

5.4 Criteria for subject termination of dosing

1. Subject voluntarily withdraws informed consent at any time;
2. Subjects found to be ineligible after enrollment;
3. Medical imaging or clinical features indicating progression of the disease;
4. Intolerable toxicity;
5. The occurrence of any clinical adverse event, laboratory test abnormality, or other condition such that the subject may no longer benefit from continued dosing;
6. Poor patient compliance which, in the judgment of the investigator, would result in compromised study results;
7. Treatment with other antineoplastic agents that interfere with the judgment of efficacy (e.g., chemotherapy, targeted therapy, or biologic therapy)
8. Unintended pregnancy
9. Death

5.5 Handling of Withdrawn Subjects

Always make every effort to complete the efficacy and safety checks at the time of withdrawal from the trial as specified in the protocol, as well as to complete the safety follow-up period, and to keep a comprehensive record of adverse events (AEs) as well as regressions. The investigator may suggest or offer new or alternative treatments to the subject, depending on the patient's condition. Non-progressing patients should continue to be followed up for imaging evaluations, if possible, until the patient begins new treatment or the disease progresses.

6. Investigational Drugs

6.1 Overview of the trial medicinal product

6.1.1 Drug Information

Anrotinib hydrochloride and labeling are provided by Chia Tai Tianqing Pharmaceutical Group Co. and Penpulimab and labeling are provided by Kangfang Tiancheng (Guangdong) Pharmaceutical Co.

Basic drug information

Drug Name	Dosage form	Specification	Method of use	Expiry Date
Anrotinib Hydrochloride Capsules	Capsule	8mg/10mg/12mg*7capsules/box	Oral	24 months
Penpulimab Injection	Injection	100mg*1 bottle/box	Intravenous drip	24 months

6.1.2 Labeling of study drug

Specific drug dispensing specifications and quantities:

Anrotinib hydrochloride capsules are polyamide/aluminum/polyvinyl chloride cold-pressed molded solid pharmaceutical composite hard tablets and pharmaceutical aluminum foil packaging, 7 capsules/plate, 1 plate/box.

Penpulimab is available as an injection (solvent-based) in 100 mg/10mL/vial. Each glass vial contains 10 mL of study drug at a concentration of 10 mg/mL. Each vial is for single use only and should not be used to treat more than one subject.

6.2 Dosage and Dosing Regimen of Drugs

6.2.1 Administration of Anrotinib hydrochloride

Take 1 capsule (10 mg) of amlotinib hydrochloride once daily on an empty stomach before breakfast. Take 2 consecutive oral doses for 2 weeks and stop for 1 week, i.e. 3 weeks (21 days) as a treatment cycle.

In the event of a missed dose, it was confirmed that the time to the next dose was shorter than 12 hours, and no additional dose would be given. Patients with disease control (CR+PR+SD) and tolerable adverse effects are continuously dosed.

6.2.2 Use of Penpulimab

6.2.2.1 Composition, Formulation of Penpulimab

Penpulimab is an injectable (solvent-based), colorless to pale yellow, clear liquid in the form of 100 mg/10 mL/vial, with each vial containing 10 mL of study drug at a concentration of 10 mg/mL. The component content of each vial of the preparation is: Penpulimab Monoclonal Antibody (100mg), Sodium Acetate (16.4mg), Sorbitol (450.0mg), Polysorbate 80 (2mg).Penpulimab needs to be stored at 2-8℃ under the protection of light, refrigeration is prohibited, and violent shaking is prohibited.

Use with an IV bag containing 100mL of saline (0.9% (w/v) sodium chloride injection). First, wipe the injection port of the infusion bag and draw from the injection bag the same amount of saline as that to be administered.

Penpulimab preparation in a volume equal to 20mL of saline, then, 20mL of Penpulimab was added to the injection bag by injection. Gently invert the infusion bag to mix the solution completely. Do not shake the infusion bag. Visually inspect the final solution. If the infusion solution is not clear or the contents contain precipitate, discard the solution and record it on the Drug Inventory Log.

Penpulimab does not contain any antimicrobial preservatives; therefore, it is important to ensure that the prepared solution is sterile and used immediately after preparation. If Penpulimab dilutions cannot be used immediately and need to be stored, the total storage time from the opening of the Penpulimab vial to the start of injectable use should not exceed 24h *2-8℃) in the refrigerator or 4h at room temperature.

6.2.2.2 Penpulimab administration

Penpulimab 200 mg is administered by intravenous infusion (Q3W, 3-week cycle) and, after dilution in an intravenous infusion bag, the entire contents of the intravenous infusion bag should be administered by intravenous infusion using a 0.2 μm or 0.22 μm in-line filter. The infusion time is 60 min ± 15 min; for subjects who cannot tolerate a 60 min ± 15 min infusion, the infusion time may be extended to a maximum of 120 min ± 15 min. After completion of the infusion, the IV tubing is

flushed with a volume of saline equal to the volume of the infusion device to ensure that all of the medication is infused into the subject.

Because the compatibility of Penpulimab with other intravenously infused drugs and solutions other than saline (0.9% (w/v) sodium chloride injection) is unknown, Penpulimab solution should not be infused using an intravenous infusion line that has been infused with other solutions or drugs. and the date, start time, interruption time, and end time of Penpulimab administration must be documented in the source file.

6.2.2.3 Risks of Administration and Monitoring

Based on the mechanism of action of Penpulimab and similar PD-1/PD-L1 blockers (e.g., Pembrolizumab, Nivolumab, Atezomab, Durvalumab, and Avelumab), potential risks include immune-mediated reactions (immune-associated adverse events, irAEs). irAEs may be associated with other anti PD-1/L1 inhibitors that present with adverse events similar to those seen with other anti-PD-1/L1 inhibitors, including immune-mediated colitis, dermatitis, pneumonia, hepatitis, encephalitis, nephritis, and endocrine disorders ([) (1) (4) (]). Based on available clinical data for anti-PD-1 monoclonal antibody drugs, despite the high incidence of adverse reactions, they are well tolerated, with only a small percentage of subjects discontinuing their medication due to adverse reactions, and most of the adverse reactions resolving with treatment.

Similar to certain therapeutic antibodies, other potential risks associated with Penpulimab include infusion-related or allergic reactions. Subjects may develop ADA, which may neutralize Penpulimab and cause infusion reactions or systemic allergic reactions, or may induce or potentiate the toxicity of Penpulimab. Standard clinical assessments and interventions for infusion-related reactions or systemic anaphylactic reactions are recommended.

The study provides exclusion criteria to ensure that subjects who may be at risk are not enrolled (i.e., exclude patients with prior autoimmune disease, inflammatory bowel disease, toxicity unrelieved by prior therapy, or problems with hematologic or organ function to avoid exacerbation of pre-existing disease by activation of the immune system). Guidelines for toxicity management have been developed based on other anti-PD-1/L1 drugs, with a focus on frequent monitoring

and early detection of potential immune-related adverse events. Measures used to manage toxicity include suspension of dosing and/or use of glucocorticoids or more potent immunosuppressive agents as needed.

6.5 Symptomatic management of common adverse reactions and dose adjustment

6.5.1 Symptomatic management of common adverse reactions to amlotinib hydrochloride and dose adjustment

Delayed Dosing and Dose Adjustment Provisions for Anrotinib Hydrochloride:

If an adverse reaction related to amlotinib occurs during the dosing period (1 to 14 days) of each cycle of amlotinib hydrochloride capsules, and if a delay in dosing is required, the dose will be administered one dose down when the adverse reaction recovers to< Grade 2. Delays should be limited to a maximum of 4 weeks, beyond which failure to recover to < Grade 2 requires permanent discontinuation of amlotinib (exceptions may be made for events not due to safety reasons).

When a dose adjustment is required for anrotinib hydrochloride, it is first adjusted downward to 10 mg once a day; if 10 mg is still not tolerated, it is then adjusted downward to 8 mg once a day. If 8 mg remains intolerant, discontinue the drug permanently.

The principles of adverse event management are as follows:

In the event of non-bleeding adverse events, refer to the principles in Table 6.3(1) for dose adjustments; in the event of bleeding adverse events, refer to Table 6.3(2) for dose adjustments.

Table 6.3(1): Principles of dose adjustment for non-bleeding events

Adverse reaction grade (NCI-CTC AE 5.0)	Time of administration	Principle of adjustment
Grade 3	Suspend dosing and wait for adverse effects to return to＜ Grade 2	After the symptoms recover to＜ Grade 2, adjust one dose downward and continue to administer the drug;

		Consider permanent discontinuation of the drug if it has not recovered to＜ Grade 2 after 4 weeks.
Grade 4	Suspend the drug until the adverse effects return to＜ level 2.	Continue dosing one dose down after symptoms return to＜ Grade 2;<br>If recovery to＜ Grade 2 has not occurred after 4 weeks, consider permanent discontinuation of the drug; or consider permanent discontinuation of the drug at the physician's discretion.

Table 6.3(2): Principles of dose adjustment for bleeding adverse events

Bleeding event*	Adjustment principle
Grade 2	Suspend the drug and treat with active symptomatic therapy; if it can recover to < Grade 2 within 4 weeks, continue the drug after the investigator judges it safe; if it occurs again, consider permanent discontinuation of the drug.
≥ Grade 3	Discontinue drug permanently and treat with urgent medical intervention.

*Bleeding adverse reactions included hemoptysis, gastrointestinal bleeding, nasal bleeding, bronchial bleeding, gingival bleeding, hematuria, fecal occult blood, and cerebral hemorrhage.

For adverse reactions occurring during the trial, investigators are advised to take medical treatment according to the actual clinical situation (the following treatments are for reference)

1) Liver function abnormalities

The following table shows the recommended regimen for delayed dosing and/or dose level changes when liver function abnormalities (elevated ALT, elevated AST, or elevated total bilirubin) occur:

Liver Function Abnormalities	Treatment Recommendations and Dose Adjustments
Level 1:	Maintain original dose and follow as planned
Grade 2 (normal at baseline).	➢ Delay dosing and if recovery to < Grade 2 can be achieved within 4 weeks, lower the dose level by one and continue dosing; if recovery to < Grade 2 is not achieved after 4 weeks, consider permanent discontinuation of the drug; or consider permanent discontinuation of the drug according to the judgment of the physician.<br>➢ Aggressive hepatoprotective treatment and close monitoring of liver function once a week
Grade 2 (baseline abnormality)	Maintain the original dose; active hepatoprotective treatment and close monitoring of liver function, once a week
Grade 3	➢ Delayed dosing, recovery to < Grade 2 within 4 weeks, reduce dosage level by one to continue; if no recovery to < Grade 2 after 4 weeks, consider permanent discontinuation; or consider permanent discontinuation based on physician's judgment.<br>➢ Aggressive hepatoprotective treatment and close monitoring of liver function twice weekly; until toxicity recovers to < grade 2 or until there is a reason for
Grade 4	Permanent discontinuation of therapy; aggressive hepatoprotective treatment and close monitoring of liver function 1-2 times per week; until toxicity returns to < grade 2 or until there is an explanation for the cause

2) Hand-foot syndrome (HFS)

Hand-foot syndrome (HFSR), which is a dulling of sensation in the palms-sole of the feet or erythema of the extremities, is a skin toxicity that occurs with more pronounced manifestations in areas of pressure or stress. It can occur in patients with tumors undergoing chemotherapy or molecularly targeted therapies.HFSR is characterized by numbness, dulled sensation, abnormal sensation, tingling, painless or painful sensation, swollen, or erythematous skin, desquamation, cracking, sclerotinia-like blisters, and severe pain.

Hand-foot syndrome	Treatment Recommendations and Dosage Adjustments
Grade 1: Numbness/dysesthesia/abnormal sensation, painless swelling or erythema of the hands and/or feet and/or discomfort that does not interfere with normal	Take some necessary symptomatic supportive treatment, including:

<table>
<tr><td>activities.</td><td rowspan="3">intensive skin care to keep the skin clean and avoid secondary infections; avoid pressure or friction; use emollient creams or lubricants, topical lotions or lubricants containing urea and corticosteroid ingredients; and topical antifungal or antibiotic treatment if necessary.</td></tr>
<tr><td>Grade 2: Painful erythema and swelling of the hands and/or feet and/or discomfort that interferes with the patient's daily activities.</td></tr>
<tr><td>Grade 3: Wet flaking, ulcers, blisters, or severe pain in the hands and/or feet and/or severe discomfort that prevents the patient from working or performing daily activities. Intense pain and loss of skin function are relatively rare.</td></tr>
</table>

Note: **If there are 3 consecutive occurrences of Grade II or higher hand and foot syndrome with a tendency for exacerbation, terminate the drug and withdraw from the clinical study.**

3) Hypertension

Before patients are enrolled in the study, they should be enrolled in strict accordance with the blood pressure requirements in the enrollment criteria. Hypertensive patients can complete their blood pressure control by adjusting the dose of antihypertensive medication or adding new antihypertensive medication before taking the experimental drug, and their blood pressure must be controlled within 140/90 mmHg before randomization (with an interval of 24 hours and above, and the average of 2 times of blood pressure monitoring).

MONITORING AND MANAGEMENT OF SUCH HYPERTENSION: Blood pressure monitoring should be performed at least 3 times per week during the first 2 cycles of initiating targeted drug therapy.

Since anti-VEGF/VEGFR targeted therapeutic agents cause a decrease in NO synthesis and eventually activate the renin-angiotensin-aldosterone system to cause hypertension, it is preferable to use angiotensin-converting enzyme (ACE) inhibitors (e.g., captopril, enalapril, benazepril, and cilazapril, etc.) to treat this type of

hypertension. Patients who are allergic or intolerant to ACE inhibitors can be treated with angiotensin II receptor blockers (ARBs, such as chloretin, valsartan, irbesartan, and timosartan, etc.) In addition to lowering blood pressure, ARBs can also be beneficial in relieving proteinuria. Angiotensin-converting enzyme inhibitors may be used in patients with chronic kidney disease, proteinuria, and metabolic syndrome; dihydropyridine calcium antagonists are indicated in elderly patients.

Patients who develop hypertension or worsening of hypertension while on the medication are recommended to use the following drugs for the treatment of hypertension: 1) angiotensin-converting enzyme inhibitors (ACEIs); 2) angiotensin II receptor antagonists (ARBs); 3) dihydropyridine calcium channel antagonists; and 4) β-blockers.

Diuretic antihypertensive agents, antihypertensive agents with CYP3A4 inhibition such as nicardipine, diltiazem, and verapamil are not recommended and are contraindicated while taking the test drug.

Dose adjustment after the development of hypertension follows the principles outlined below:

Hypertension	Management Recommendations and Dose Adjustments
Systolic blood pressure 120-139 mmHg or diastolic blood pressure 80-89 mmHg	Close monitoring and maintenance of the original dose level
Grade 2 hypertension without symptomatic manifestations: persistent (≥24h) or reoccurring systolic blood pressure to 140-159 mmHg or diastolic blood pressure to 90-99 mmHg; diastolic blood pressure ≥20 mmHg higher than before	➢ Maintain original dose level<br>➢ Initiation of antihypertensive medication or adjustment of existing antihypertensive medication dose<br>➢ Effective control of blood pressure through two weeks of antihypertensive drug titration<br>(systolic blood pressure <140 mmHg, diastolic blood pressure <90 mmHg), continue to use the drug at the original dose level; if blood pressure is not effectively controlled within 2 weeks, consider permanent discontinuation of the drug;

Grade 2 hypertension with symptomatic manifestations, or Grade 3 hypertension: systolic blood pressure ≥160 mmHg or diastolic blood pressure ≥100 mmHg; or Grade 2 hypertension that cannot be effectively controlled within two weeks	➢ Suspension of medication<br>➢ Starting antihypertensive medication or adjusting the dose of existing antihypertensive medication<br>➢ Effective control of blood pressure (systolic blood pressure <140 mmHg and diastolic blood pressure <90 mmHg) through two weeks of antihypertensive medication titration, adjust the medication downward by one dose or maintain it at the original dose level (based on the investigator's judgment), and consider permanent discontinuation of the medication if the blood pressure is not under effective control within 2 weeks;

Note: **After antihypertensive drug treatment and dose adjustment of amlotinib, there is still the occurrence of multiple (two or more) symptoms of hypertension or the development of hypertensive crises, the amlotinib dosing should be terminated to withdraw from the pilot study.**

4) Bleeding

The occurrence of gastrointestinal bleeding, including fecal occult blood (++) and above, vomiting blood or fresh blood stools, should be actively treated symptomatically.

Patients with upper gastrointestinal bleeding should be fasted and given antacid, protection of gastric mucosa, hemostasis (hemostatic cyclic acid, lisinopril, etc.), and octreotide can be used if necessary; patients with lower gastrointestinal bleeding should be given hemostasis, blood transfusion, and supportive therapy, etc.; for those with uncontrollable bleeding, it is necessary to immediately ask for surgical assistance to deal with the bleeding.

Patients with coughing blood or hemoptysis should be given hemostasis, blood transfusion and supportive therapy; for those with uncontrolled bleeding, surgical assistance should be requested.

In the event of bleeding and coagulation abnormalities (except for cerebral hemorrhage and grade 2 or higher pulmonary hemorrhage), the following principles should be followed for dose adjustment:

Bleeding and coagulation abnormalities	Treatment recommendations and dose adjustment

Grade 1	Close monitoring, maintain the original dose level of amlotinib
Grade 2	Suspend drug and wait for adverse events to recover to < grade 2, then continue drug at one dose level down and monitor closely; if no recovery after 4 weeks, consider permanent discontinuation;
Recurrence of ≥ grade 3 or ≥ grade 2 (after discontinuation/dose adjustment)	Withdrawal from the pilot study

Note: ***Confirmed diagnosis of cerebral hemorrhage, grade II or higher pulmonary hemorrhage, and grade III or higher hemorrhage requires immediate discontinuation of the drug, symptomatic management and withdrawal from the pilot study.***

5) Proteinuria

Closely monitor proteinuria in all patients during the entire treatment period, and strengthen the monitoring of those with a history of hypertension; for those who have urinary protein≥++ for 2 consecutive times, 24-hour urine protein measurement should be performed.

Dose adjustments after proteinuria are based on the following principles:

Proteinuria	Treatment recommendations and dose adjustments
Grade 1: Urine protein+ or 24-hour urine protein less than 1.0g.	Maintain the original dose level of amlotinib
Grade 2: Urine protein++ ~+++ or 24-hour urine protein 1.0-3.4g	➢ For symptomatic treatment, maintain the original dose level of amlotinib and continue the drug if proteinuria returns to ≤ grade 1 within 4 weeks;<br>➢ If proteinuria does not improve or worsens, suspend amlotinib and continue amlotinib at the original dose level if proteinuria recovers to ≤ grade 1 within 4 weeks; and, if proteinuria recovers to ≤ grade 1 within 4 weeks then continue the drug;<br>➢ If the proteinuria does not return to ≤ grade 1 after 4 weeks then discontinue the drug permanently.

Grade 3: urine protein++++ or 24-hour urine protein >3.5g	➢ Suspend dosing and treat symptomatically; if proteinuria recovers to ≤ grade 1 within 4 weeks, then continue dosing with a downward adjustment of one dose level of amlotinib; permanently discontinue dosing if it has not recovered to ≤ grade 1 after 4 weeks.

Note: **If nephrotic syndrome develops, discontinue the drug permanently and withdraw from this clinical study.**

6) Thrombosis

In the event of any arterial thrombosis (e.g., cerebral ischemia, stroke, angina pectoris, myocardial infarction, etc.), discontinue the drug immediately and withdraw from the pilot study. If symptomatic IV venous thrombosis occurs, the drug should be discontinued and withdrawn from the pilot study.

Symptoms of thrombosis should be treated immediately with symptomatic therapy, surgery, or anticoagulant medication.

In the event of venous thrombosis, the principles to be followed for dose adjustment are as follows:

**Venous thrombosis**	**Treatment recommendations and dose adjustments**
Grade 2	Maintain the original dose level of amlotinib with close monitoring
Grade 3 or asymptomatic Grade 4	➢ Discontinuation of erlotinib<br>➢ Patient is symptomatically treated with anticoagulants (small molecular weight heparin)<br>➢ Anticoagulants are used for at least one full week, and when thrombotic symptoms improve and no patients with severe (grade 3 or 4) bleeding have occurred, the drug may be continued at one dose down, at the investigator's discretion; if there is no return to ≤ grade 1 after 4 weeks the drug is permanently discontinued.

8) Fatigue and weakness

Fatigue and weakness are common tumor-related clinical symptoms, electrolyte disorders, liver function abnormalities, cardiac function abnormalities, etc. may lead to fatigue and weakness. At the same time, fatigue and weakness are also common clinical adverse effects of neovascular targeting agents such as sunitinib, pazopanib and sorafenib. Clinical reports suggest that neovascular-targeted agents may increase the incidence of fatigue and weakness by causing

hypothyroidism.

In previously completed clinical trials of amlotinib, the incidence of fatigue and weakness was higher in patients in the amlotinib dosing group than in patients in the control group, and the specific mechanism by which amlotinib triggers an increased incidence of fatigue and weakness is unclear.

Therefore, patients should be taken seriously when they present with, and report, grade 2 or higher fatigue and weakness;

When grade III and above fatigue and weakness occurs, patients should be immediately admitted to the hospital for examination, one by one, to exclude electrolyte disorders, liver function abnormalities, cardiac function disorders (electrocardiogram, cardiac ultrasound), hormone level abnormalities (adrenal hormones, thyroid hormones) and other possible causes, to be treated as symptomatic, and according to the principle of dosage adjustment, the dose of the test drug should be suspended or adjusted accordingly.

9) Abdominal pain

Abdominal pain is not rare in Anrotinib lung cancer treatment, and it is mostly a symptom accompanying the tumor. Meanwhile, gastrointestinal perforation occasionally occurs in clinical trials of Anrotinib and other types of anti-neovascular drugs. Investigators should be alert to the possibility of gastrointestinal perforation in patients presenting with abdominal pain. Note: **If gastrointestinal perforation is detected, the drug must be immediately discontinued, the trial withdrawn, and aggressive symptomatic treatment instituted.**

6.5.2 Management of Penpulimab Adverse Reactions and Dose Adjustments

Guidelines for treatment adjustment and toxicity management of infusion-related reactions, immune-related AEs, and non-immune-related AEs associated with the study drug Penpulimab are provided in the table below, respectively. Additional guidelines for therapeutic adjustments and toxicity management for other immune-related AEs not mentioned in the table can be found in the Management of Immune-Related Adverse Events in Subjects Treated with Immune Checkpoint Inhibitors : Clinical Practice Guidelines of the American Society of Clinical Oncology (Brahmer JR, 2018).

6.5.2.1 Infusion reactions

Subjects will be monitored for signs and symptoms of infusion reactions (e.g., fever and/or chills, flushing and/or itching, changes in heart rate and blood pressure, dyspnea or chest discomfort, rash, etc.) and systemic anaphylactic reactions (e.g., generalized urticaria, angioedema, asthma, hypotension, tachycardia).

In this study, to avoid confounding potential safety signals regarding the assessment of infusion reactions:

1) Implementation of primary prevention (prophylaxis in subjects who did not experience an infusion event) for infusion reactions was not permitted in this study.
2) Appropriate secondary prophylaxis (i.e., prophylaxis of infusion-related reactions after the initial episode) is at the discretion of the investigator:

- Acetaminophen/paracetamol and/or antihistamines (e.g., diphenhydramine) may be administered approximately 30 min prior to the start of the subsequent infusion and/or glucocorticoids or equivalents may be given according to each center's diagnostic and treatment routine.
- Non-sedating antihistamines (e.g., cetirizine) may be considered for subjects with recurrent infusion reactions.
- If symptoms persist after these treatments, glucocorticoid therapy should be considered.
- Pethidine/morphine sulfate and promethazine or their equivalents may be given via IV before the start of the subsequent infusion at the discretion of the investigator, depending on each center's practice.

Repeated doses of pretreatment medications may be required during the infusion; therefore, this should be taken into account when determining the dose of pretreatment medications. Any significant reactions and requirements for use related to glucocorticoids must be discussed with the Medical Monitor.

In the event of a ≤ Grade 2 infusion reaction, the Penpulimab infusion rate may be reduced by 50% or the infusion may be interrupted until the event subsides (up to 4 h) and restarted at 50% of the initial infusion rate after the event has subsided until completion of the infusion. Once the infusion rate of Penpulimab is reduced

by 50% or the infusion is interrupted due to an infusion reaction, all subsequent infusions must continue at the reduced infusion rate. If the subject experiences a second≥ Grade 2 infusion reaction at the slower infusion rate, the infusion should be stopped and the subject must discontinue Penpulimab therapy. If a Grade 3 or Grade 4 infusion reaction occurs at any time in a subject, treatment with Penpulimab must be discontinued.

If a severe hypersensitivity reaction (CTCAE Grade 3 or 4) is observed during the infusion, the infusion will be stopped immediately and no further Penpulimab treatment will be given to the subject. Supportive treatment will be provided in accordance with standard medical practice. Full guidance from the Resuscitation Council (UK) Working Group on the emergency treatment of systemic anaphylactic reactions can be found at https://www.resus.org.uk/anaphylaxis/emergency-treatment-of-anaphylactic-reactions/. If a subject develops signs or symptoms of a systemic anaphylactic reaction or Type 1 hypersensitivity reaction during Penpulimab administration, he/she will be treated with appropriate medications and medical equipment, which will be readily available at all study sites. Glucocorticoids, epinephrine, allergy medications (antihistamines), or equivalents should be readily available. Subjects should be advised to report any delayed reactions to the investigator immediately.

Penpulimab treatment adjustments related to infusion reactions and recommended toxicity management guidelines are shown in the table below. ***In the event of multiple concurrent low-grade AEs (which, when occurring alone, would not require permanent discontinuation of Penpulimab therapy), permanent discontinuation of Penpulimab therapy will be at the discretion of the investigator.***

Treatment Adjustments for Penpulimab-Induced Infusion Reaction Symptoms

NCI-CTCAE Grade	Treatment Adjustments for Penpulimab
**Grade 1 - Mild**<br>Mild, transient reaction; no need to interrupt infusion; no treatment required.	➢ Reduce Penpulimab infusion rate by 50% and monitor closely for any deterioration.<br>➢ The total infusion time of Penpulimab should not exceed 120 min.
**Grade 2 - Moderate**<br>Treatment or infusion interruption required, but symptomatic (e.g., antihistamines, NSAIDs, glucocorticoids, IV fluids) with rapid	➢ Interruption of Penpulimab infusion.<br>Restart the infusion at 50% of the previous infusion rate when the infusion reaction has subsided or decreased in severity to at least Grade 1, and monitor closely for any

NCI-CTCAE Grade	Treatment Adjustments for Penpulimab
onset of action; prophylactic administration < 24 h.	deterioration.<br>➢ *If a subject experiences a second≥ Grade 2 infusion reaction at a slower infusion rate, the infusion should be discontinued and the subject withdrawn from Penpulimab treatment.*
**Grade 3 or 4 - Severe or Life-Threatening**<br>Grade 3: Delay in symptomatic relief (e.g., failure to respond rapidly to symptomatic therapy and/or infusion interruptions); recurrence after symptomatic improvement; hospitalization required to treat sequelae.<br>Grade 4: Life-threatening; requires urgent treatment.	➢ Immediately stop Penpulimab infusion and disconnect the subject from the infusion line.<br>➢ *Subject must be immediately withdrawn from Penpulimab therapy and must not receive any further Penpulimab therapy.*

Abbreviations: IV = intravenous; NCI-CTCAE = National Cancer Institute Common Terminology Criteria for Adverse Events; NSAID = nonsteroidal anti-inflammatory drug.

6.5.2.2 Immune-related Adverse Events (irAE)

An irAE is defined as an AE associated with drug exposure that is consistent with an immune-mediated mechanism of action in which there is no clear alternative etiology. Based on the mechanism of action of Penpulimab, which targets PD-1, leading to T-cell activation and proliferation, it is possible that irAEs were observed during the conduct of this study. potential irAEs may be similar to the adverse events that occur with the use of anti-PD-1/L1 medications, including immune-mediated colitis, dermatitis, pneumonitis, hepatitis, encephalitis, nephritis, and endocrinopathy. Subjects should be monitored for signs and symptoms of irAE. In the absence of other alternative etiologies (e.g., infection or PD), immune-related etiologies associated with signs or symptoms of colitis, dermatitis, pneumonia, hepatitis, encephalitis, nephritis, and endocrine disease should be considered.

Penpulimab treatment adjustments will be made to manage potential irAE based on the severity of toxicity occurring on therapy as graded according to the NCI CTCAE version 5.0 The maximum interval allowed for Penpulimab drug suspension is 12 weeks. If return to a state where Penpulimab can be reintroduced within 12 weeks is not possible, the subject permanently discontinues Penpulimab and enters the follow-up phase. Except in the following two cases: glucocorticoids are applied for the treatment of irAE and the glucocorticoid tapering process results in the

suspension of Penpulimab for more than 12 weeks or for the treatment of an AE that may or may not be unrelated to Penpulimab, resulting in the suspension of Penpulimab for more than 12 weeks. In such cases, a decision on whether Penpulimab treatment can be continued needs to be discussed with the Medical Monitor.

In general, it is recommended that irAE be managed in accordance with the guidelines described below. subjects should be thoroughly evaluated to exclude any other alternative etiologies (e.g., disease progression, coadministration of medications, infections, etc.). Serologic, immunologic, and histologic (biopsy) data should be used, as appropriate, to support the diagnosis of irAE.

- In the absence of a clear alternative etiology, all events should be considered as possibly immunologically related.
- Symptomatic and localized therapy should be considered for low-grade (Grade 1 or 2, unless otherwise specified) events.
- Systemic glucocorticoid (e.g., prednisone or intravenous equivalent) therapy should be considered for persistent low-grade or severe (≥ grade 3) events.
- If symptoms recur or worsen during glucocorticoid tapering, increase the glucocorticoid dose until symptoms stabilize or improve, and then re-taper the glucocorticoid at a slower rate.
- For events that do not respond to systemic glucocorticoids, a more potent immunosuppressive agent, i.e., a TNF antagonist (e.g., infliximab, mescaline), should be considered after discussion with the medical monitor.
- Discontinuation of investigational drug therapy is not mandatory for grade 3 or 4 inflammatory responses (e.g., metastatic disease sites, inflammatory responses in lymph nodes) due to a local tumor response.
- A consultation with a physician in another field (e.g., cardiology or autoimmune disease specialty) should be arranged promptly so that can make appropriate treatment decisions.
- Discontinuation of Penpulimab treatment will be at the discretion of the Investigator in the event of multiple concurrent low-grade AEs (which alone

would not require discontinuation of treatment).

- Following discontinuation of study drug therapy due to a Grade 3 or 4 event, Penpulimab therapy may be restarted after discussion with the Medical Monitor if the subject rapidly develops a favorable response and the toxicity resolves rapidly.
- For abnormal laboratory test results, the decision to suspend or permanently discontinue dosing should be based on concomitant clinical signs/symptoms and in accordance with the investigator's clinical judgment.

Guidelines for Penpulimab treatment modification and toxicity management for immune-related AEs are provided in the table below.

Penpulimab Dose Adjustments and Recommended Toxicity Management Guidelines for Penpulimab-Associated irAEs

**Gastrointestinal irAE**<br>(Rule out non-inflammatory causes. If a non-inflammatory cause is determined, treat accordingly and continue Penpulimab therapy. Opioids/anesthetics may mask perforation symptoms. Infliximab should not be used in cases of perforation or sepsis.)		
**Severity of diarrhea/colitis (NCI-CTCAE v5.0)**	**Treatment**	**Follow-up**
**Grade 1**<br>• Diarrhea: increase in stool frequency <4 times per day from baseline; mild increase in fistula discharge<br>• Colitis: asymptomatic; seen only on clinical examination or diagnosis; no treatment required	• Continue Penpulimab treatment<br>• Symptomatic treatment (e.g., loperamide)	• Monitor closely for worsening of symptoms<br>• Instruct subjects to report deterioration immediately if it occurs<br><u>If worsening:</u><br>• Treat as grade 2 or 3-4
**Grade 2**<br>• Diarrhea:	• Delayed Penpulimab	<u>If improvement to grade 1:</u><br>• Restart Penpulimab therapy (if

increased frequency of stools 4-6 times per day compared to baseline; IV rehydration <24 h, moderate increase in fistula discharge.<br>• Colitis: abdominal pain; mucus or bloody stools	treatment<br>• Symptomatic treatment	glucocorticoids have been given, glucocorticoid dose should be tapered before restarting therapy)<br>If persistent for > 5 to 7 days or relapse:<br>• 0.5 to 1.0 mg/kg/day methylprednisolone or equivalent<br>• When symptoms improve to Grade 1, at least taper the glucocorticoid dose over 1 month, consider prophylactic antibiotics to prevent opportunistic infections, and then restart Penpulimab therapy according to the regimen.<br>If symptoms worsen or persist for > 3 to 5 days after oral glucocorticoids:<br>• Treat as Grade 3 to 4
**Grade 3 to 4**<br>• Diarrhea (Grade 3): increase in stool frequency ≥ 7 times per day from baseline; fecal incontinence; requires hospitalization; severe increase in fistula discharge; interferes with personal activities of daily living<br>• Colitis (grade 3): severe abdominal pain; change in bowel habits; need for medical treatment; positive signs of peritoneal irritation	Grade 3: delayed Penpulimab treatment<br>Grade 4: permanent discontinuation of Penpulimab therapy<br>• Methylprednisolone or equivalent administered intravenously at 1.0 to 2.0 mg/kg/day<br>• Add prophylactic antibiotics to prevent opportunistic infections<br>• Consider lower abdominal endoscopy	If improved:<br>• Continue glucocorticosteroid therapy until resolution to grade 1, then taper over at least 1 month<br>If persists > 3 to 5 days, or recurs after improvement:<br>• Increase infliximab 5 mg/kg (if not contraindicated) Note: Infliximab should not be used in cases of perforation or sepsis.

• Grade 4: life-threatening, perforation is life-threatening; requires urgent treatment		
**Skin irAE**<br>(Rule out non-inflammatory causes. If a non-inflammatory cause is determined, treat accordingly and continue Penpulimab therapy)		
**Skin Rash Grade (NCI-CTCAE v5.0)**	**Treatment**	**Follow-up**
**Grade 1 to 2**<br>Coverage≤ 30% of body surface area	• Symptomatic treatment (e.g., antihistamines, topical glucocorticoids)<br>• Continue Penpulimab treatment	If persistent for > 1 to 2 weeks or recurrence:<br>• Consider skin biopsy<br>• Delay Penpulimab treatment<br>• Consider IV methylprednisolone 0.5 to 1.0 mg/kg/day or equivalent. If improvement, taper glucocorticoid dose over at least 1 month, consider prophylactic antibiotics to prevent opportunistic infections, and restart Penpulimab therapy<br>If worsening:<br>• Treat as grade 3 to 4
**Grade 3 to 4**<br>Coverage of > 30% of body surface area;	Grade 3: delay Penpulimab treatment<br>Grade 4: permanent discontinuation of Penpulimab treatment<br>• Consider skin biopsy<br>• Dermatology consultation<br>• Methylprednisolone or equivalent administered intravenously at	If grade 3 rash does not improve to≤ grade 1 or baseline level within 30 days after temporary delay of Penpulimab, then<br>• Permanently discontinue Penpulimab therapy.<br>If improvement to Grade 1:<br>• Taper the glucocorticoid dose for at least 1 month and increase prophylactic antibiotics to prevent opportunistic infections.<br>• Restart Penpulimab therapy (for grade 3 events that improve to grade 1)

	1.0 to 2.0 mg/kg/day	
**Pulmonary irAE**<br>(Rule out non-inflammatory causes. If a non-inflammatory cause is determined, treat accordingly and continue Penpulimab therapy. Evaluate with imaging and lung condition consult results)		
**Pneumonia Grade (NCI-CTCAE v5.0)**	**Treatment**	**Follow-up**
**Grade 1**<br>Asymptomatic; clinical examination or diagnostic findings only; no intervention required	• Continue Penpulimab treatment<br>• Monitor symptoms every 2-3 days<br>• Consider respiratory and infectious disease specialty consultation	• Re-imaging at least every 3 weeks<br>If worsening:<br>• Treat as grade 2 or 3-4
**Grade 2**<br>Symptomatic; requires intervention; interferes with instrumental activities of daily living	• Delay Penpulimab treatment<br>• Respiratory and infectious disease specialty consultation<br>• Monitor symptoms daily; consider hospitalization<br>• Methylprednisolone or equivalent via IV 1.0 mg/kg/day<br>• Consider bronchoscopy, lung biopsy	• Re-imaging every 1 to 3 days<br>If improvement:<br>• When symptoms return to near baseline levels, taper glucocorticoid dose for at least 1 month, then restart Penpulimab therapy and consider prophylactic antibiotics<br>If no improvement or worsening after 2 weeks<br>• Treat as grade 3 to 4
**Grade 3 to 4**<br>Severe symptoms; limited personal autonomy; need for	• *Permanent discontinuation of Penpulimab therapy*	If improvement to baseline levels:<br>• Taper glucocorticosteroid dose over at least 6 weeks

oxygen; life-threatening respiratory compromise; need for emergency treatment (tracheotomy or intubation)	• Hospitalization<br>• Respiratory and infectious disease specialist consultation<br>• Methylprednisolone or equivalent via IV 2 to 4 mg/kg/day<br>• Add prophylactic antibiotics for prevention of opportunistic infections<br>• Consider bronchoscopy, lung biopsy	If no improvement or worsening after 48 h:<br>• Increase other immunosuppression (e.g., infliximab, cyclophosphamide, IVIG, or mortimecrolide).
**Liver irAE**<br>(Rule out non-inflammatory causes. If a non-inflammatory cause is determined, treat accordingly and continue Penpulimab therapy. Consider imaging the obstruction)		
**Elevated grade of liver test parameters (NCI-CTCAE v5.0)**	**Treatment**	**Follow-up**
**Grade 1**<br>Grade 1 AST or ALT > ULN to 3.0 × ULN and/or TBil > ULN to 1.5 × ULN	• Continue Penpulimab treatment	• Continue to monitor liver function<br>If worsening:<br>• Treat as grade 2 or 3-4
**Grade 2**<br>AST or ALT > 3.0 to ≤ 5 × ULN and/or TBil > 1.5 to ≤ 3 × ULN	• Delay Penpulimab treatment<br>• Increase monitoring frequency to every 3 days	If return to baseline levels:<br>• Resume routine monitoring and restart Penpulimab therapy<br>If elevation persists > 5 to 7 days or worsens:<br>• 0.5 to 1 mg/kg/day methylprednisolone or equivalent, when LFTs return to grade 1 or baseline, taper glucocorticosteroid dose for at least 1 month, consider prophylactic antibiotics for

		opportunistic infections, and restart Penpulimab therapy
**Grade 3 to 4**<br>AST or ALT > 5 × ULN and/or TBil > 3 × ULN	• Permanently discontinue Penpulimab therapy (may delay Penpulimab administration if AST/ALT ≤ 8 × ULN and total bilirubin ≤ 5 × ULN)<br>• Increase monitoring frequency to every 1-2 days<br>• 1.0 to 2.0 mg/kg/day of methylprednisolone or equivalent intravenously (in the presence of grade 4 hepatitis, the recommended starting dose is 2.0 mg/kg/day of methylprednisolone or equivalent intravenously)<br>• Addition of prophylactic antibiotics to prevent opportunistic infections<br>• Consultation with gastroenterologist<br>• Consider magnetic resonance imaging (MRI)/computed tomography (CT) of the liver, or	If recovery to grade 2:<br>• Taper the glucocorticoid dose over at least 1 month.<br>• If no improvement, worsening, or rebound within > 3 to 5 days:<br>• Increase 1 gram (g) of mescaline twice daily (BID)<br>• If no response in additional 3-5 days, consider other immunosuppressive agents according to local guidelines.

	liver biopsy, if clinically warranted.	
**Renal irAE**<br>(Rule out non-inflammatory causes. If a non-inflammatory cause is determined, treat accordingly and continue Penpulimab therapy)		
**Elevated creatinine grade (NCI-CTCAE v5.0)**	**Treatment**	**Follow-up**
**Grade 1**<br>Creatinine > ULN and > baseline level, but ≤ 1.5 × baseline level	• Continue Penpulimab treatment<br>• Monitor creatinine levels weekly	If return to baseline level:<br>• Restart routine creatinine monitoring according to protocol<br>If worsening:<br>• Treat as grade 2 to 3 or 4
**Grade 2 to 3**<br>Creatinine > 1.5 × baseline and ≤ 6 × ULN	• Delay Penpulimab treatment<br>• Monitor creatinine levels every 2 to 3 days<br>• Methylprednisolone or equivalent via IV 0.5 to 1.0 mg/kg/day<br>• Consider renal biopsy	If recovery to grade 1:<br>• Taper glucocorticoid dose over at least 1 month, consider prophylactic antibiotics to prevent opportunistic infections, restart Penpulimab therapy according to regimen, and begin routine creatinine monitoring If elevation persists for > 7 days or worsens:<br>• Treat as grade 4
**Grade 4**<br>Creatinine > 6 × ULN	• Discontinue Penpulimab therapy permanently<br>• Monitor creatinine level once daily<br>• Methylprednisolone or equivalent administered intravenously at	If recovery to grade 1:<br>• Taper glucocorticosteroids for at least 1 month and add prophylactic antibiotics to prevent opportunistic infections

	1.0 to 2.0 mg/kg/day<br>• Nephrology consultation<br>• Consider renal biopsy	
**Neurologic irAE**<br>(Rule out non-inflammatory causes. If a non-inflammatory cause is determined, treat accordingly and continue Penpulimab therapy)		
**Neurotoxicity grade (NCI-CTCAE v5.0)**	**Treatment**	**Follow-up**
**Grade 1**<br>Asymptomatic or mildly symptomatic; no therapeutic intervention required	• Continue Penpulimab treatment	• Continued monitoring of subjects<br>If worsening:<br>• Treat as grade 2 or 3-4
**Grade 2**<br>Moderate symptoms; affects instrumental activities of daily living	• Delay Penpulimab treatment<br>• Treat symptoms according to local guidelines<br>• Consider intravenous 0.5 to 1.0 mg/kg/day methylprednisolone or equivalent	If improvement to baseline levels:<br>• Restart Penpulimab treatment<br>If worsening:<br>• Treat as grade 3 to 4
**Grade 3 to 4**<br>Severe symptoms; limited personal autonomy; life-threatening; requires urgent intervention	• Permanent discontinuation of Penpulimab therapy<br>• Neurology consultation<br>• Treat symptoms according to local guidelines<br>• Consider intravenous methylphenidate 1.0 to 2.0 mg/kg/day	If improvement to grade 2:<br>• Taper the glucocorticoid dose over at least 1 month.<br>If worsening or atypical presentation:<br>• Consider IVIG or other immunosuppressive therapy according to local guidelines

	methyl prednisolone or equivalent<br>• Add prophylactic antibiotics to prevent opportunistic infections	
**Endocrine irAE**<br>(Rule out non-inflammatory causes. If a non-inflammatory cause is identified, treat accordingly and continue Penpulimab therapy. Consider visualization, endocrinology consultation and imaging)		
**Grade of endocrine toxicity (NCI-CTCAE v5.0)**	**Treatment**	**Follow-up**
**Grade 1**<br>(depending on type of endocrinopathy, refer to NCI CTCAE v5.0 for definition of CTCAE Grade 1)	No dose adjustment	For Grade 1 (including subjects with asymptomatic TSH elevation):<br>• Perform appropriate endocrine function tests to monitor subject<br>• If TSH < 0.5 × LLN or TSH > 2 × ULN, or if results are consistently out of range on 2 subsequent measurements, add free T4 levels to subsequent cycles as clinically indicated and consider endocrinology consultation
**Level 2**	Continue, suspend, or permanently discontinue	For Level 2 (including subjects with symptomatic endocrine disease):<br>• Initiate hormone replacement therapy as needed<br>• Evaluate endocrine function and consider pituitary scanning as clinically indicated<br>• For subjects with abnormal endocrine function, short-term high-dose glucocorticoid therapy (e.g., 1 to 2 mg/kg/day methylprednisolone or intravenous equivalent) and

		related hormone replacement therapy (e.g., levothyroxine, hydrocortisone, or sex hormones)<br>• If improvement occurs, gradually reduce glucocorticoid dose over ≥ 4 weeks and administer prophylactic antibiotics to prevent opportunistic infections.<br>• Repeat laboratory tests/MRI as clinically indicated in subjects with normal endocrine function (lab or MRI scan)<br>• In cases of pituitary inflammation, adrenocortical insufficiency, hypothyroidism, and type 1 diabetes mellitus, treat with alternative therapies without interruption of therapy or glucocorticoids.
**Level 3**	Continue, suspend or permanently discontinue medication	• Initiate IV glucocorticoid therapy (e.g., methylprednisolone IV or equivalent) at a dose of 1 to 2 mg/kg/day<br>• Treat pituitary inflammation, adrenocortical insufficiency, hypothyroidism, and type 1 diabetes mellitus with alternative therapies without interrupting therapy or glucocorticoids<br>• Hormone replacement therapy if necessary<br>• For adrenal crisis, severe dehydration, hypotension, or shock: start intravenous glucocorticoids with salocorticoid activity immediately<br>• Endocrinology consultation

		• If improvement occurs, gradually reduce glucocorticoid dose over ≥ 4 weeks and administer prophylactic antibiotics to prevent opportunistic infections
**Grade 4**	Continue, suspend, or permanently discontinue	Same treatment as for grade 3 events above

For other immune-related AEs: suspend dosing for intolerable/persistent Grade 2 AEs, suspend or permanently discontinue for Grade 3 AEs, and permanently discontinue for Grade 4 AEs or recurrent Grade 3 AEs.

Subjects receiving IV glucocorticosteroids may be converted to an equivalent dose of oral glucocorticosteroids (e.g., prednisone) at the start of the taper or earlier if sustained clinical improvement is observed. When converting to an equivalent dose of oral glucocorticoid, the low bioavailability of oral glucocorticoid should be considered.

If the above principles are inconsistent with the guidelines, please refer to the guidelines after communicating with the sponsor's medical manager.

6.5.2.3 Non-Immunization-Related Adverse Events

For Grade 3 and higher non-immunization-related adverse events associated with Penpulimab, Penpulimab administration may be suspended and clinical management given. Missing doses will not be given thereafter. For AEs clearly not attributable to Penpulimab or abnormal laboratory tests that are not clinically significant, no missing doses will be given and no dose reductions will be allowed.

6.6 Medication Adherence

The Co-organizer will provide a medication distribution log, which must be kept up-to-date by the investigator with the following information:

- Identifying information of the patient receiving the medication
- Date and quantity of medication dispensed
- Date and quantity of medication returned by the patient
- Inventory must be available for inspection by the clinical supervisor. All unused anrotinib capsules and penpulimab injection must be returned by the

patient to the investigator at the beginning of the next cycle and by the investigator to the co-sponsor at the end of the study.

– The investigator should verify the medications (if any) taken by the patient, calculate adherence, and encourage the patient to use the appropriate dose of amlotinib hydrochloride/penpulimab injection according to the treatment plan. Therefore, investigators should encourage patients to report the number of medications not taken, including all acquired boxes/vials (unused or empty boxes/vials) for the purpose of calculating the number of medications.

6.7 Administration, Dispensing and Recall of Medications

6.7.1 Dispensing of medication

Dispensing of drugs should be strictly managed. The center should have a person in charge of issuing and storing the drugs, and the storage conditions of Anrotinib should be shaded from light, airtight, and kept below 25℃ . Detailed records of drug collection and use should be filled in by the investigator at each visit.

Penpulimab is stored at 2-8°C℃ , protected from light. Avoid exposure to temperatures outside the recommended range and avoid violent shaking. If the storage temperature is higher than 8℃ or lower than 2℃ , a temperature excursion needs to be reported and the co-sponsor notified in a timely manner so that the co-sponsor can evaluate the effect of the excursion on the product.

In accordance with GCP requirements, investigational drugs are stored, dispensed and recalled by the trial unit.

Patients start on the first day of the first cycle in which they receive the complimentary medication, and when the medication is dispensed on the first day of each subsequent cycle, the investigator should:

- Indicate the number of vials of Penpulimab injection or the number of capsules of different doses of anrotinib that the patient will need to use in the next treatment cycle
- Record this information on the CRF page of the study medication and record it in the medication logbook

- Instruct patients to adhere to the treatment regimen and report all unused capsules/and all drug boxes at each visit, including empty boxes and boxes still containing capsules.

6.7.2 Counting of medications

Adherence should be verified by the investigator or research nurse or CRC **at each follow-up visit**. The first day of each cycle is required:

- Ask the patient about the number of Anrotinib capsules/ Penpulimab injection vials used per day during the previous cycle
- Record this information on the treatment page of the CRF
- Calculate the number of Anrotinib capsules reported by the patient
- Record the number of Anrotinib capsules purchased and remaining/Penpulimab injection vials in the appropriate section of the CRF
- Determine whether the patient used all medications according to the study plan in the previous cycle, record the patient's remaining number of Anrotinib capsules/Penpulimab injection dose on the CRF's Anrotinib Capsules Count/Penpulimab Injection Dose Recall page at each patient visit, document the gap between the two (with an explanation) and the need to adjust the treatment record to the actual medication dosage.

6.7.3 Drug recall

The total quantity of the test drug is 120% of the design dosage, and the remaining test drug should be returned to the co-organizer Zhengda Tianqing Group Co. Ltd. at the end of the trial, and the remaining drug should be recovered by the supervisor.

After notifying the co-organizer in writing, the investigator may destroy the used drugs (non-nationally controlled chemicals) and empty boxes on the premise of not endangering the health of the population. The investigator should maintain records of all trial drug disposals.

These records must show the certification and quantity of drugs destroyed in

each batch, as well as the method of disposal (in accordance with the requirements of local law), and the person who disposed of the drugs.

6.8 Concomitant Medication

6.8.1 Drugs to be used with caution during the study period

1) Drugs that interfere with hepatic P450 enzymes

Anrotinib has a strong inhibitory effect on CYP3A4, YP2C9 and CYP2C19 ($IC_{(50)}$ <0.5 μM), and should be used with caution during treatment with inducers (dexamethasone, catamizine, rifampicin, and phenobarbital) and inhibitors (ketoconazole, itraconazole, erythromycin, and clasen) of CYP3A4, substrates (simvastatin, cyclosporine, and pemirol) of CYP3A4, and other drugs metabolized by CYP3A4 (e.g., benzodiazepines, dihydropyridines, calcium antagonists and HMG-COA reductase). and permethrin), and other drugs metabolized by CYP3A4 (e.g., benzodiazepines, dihydropyridines, calcium antagonists, and HMG-COA reductase inhibitors). Use substrates of CYP2C9 and CYP2C19 with caution, as detailed in the table below.

P450 enzymes	Substrate
CYP2C9	Diclofenac, phenytoin, piroxicam, S-warfarin, toluene sulfobutylurea
CYP2C19	Diazepam, promethazine, lansoprazole, S-mephenytoin

2) Drugs that cause prolongation of the cardiac QT interval

Due to the clinical toxicity of prolonging the QT interval with anrotinibtinib analogs, caution is required in the use of drugs that prolong the QT interval during the pilot study. This primarily includes, but is not limited to, the classes of drugs listed below:

1) Antimicrobials (clarithromycin, azithromycin, erythromycin, roxithromycin, metronidazole, moxifloxacin);

2) Antiarrhythmics (quinidine, sotalol, amiodarone, propyzamide, procainamide);

3) antipsychotics (risperidone, fluphenazine, haloperidol, haloperidol, thioridazine, pimozide, olanzapine, clozapine);

4) Antifungals (fluconazole, ketoconazole);

5) antimalarials (mefloquine, chloroquine);

6) antidepressants (amitriptyline, promethazine, clomipramine, dutasteride, doxepin).

6.8.2 Prohibited Medications during the Study Period

1) Any other investigational drug.

2) Any anticancer therapy, antibody-based therapy, retinoid therapy, hormone therapy, treatment with nitrosoureas, mitomycin C, small molecule tyrosine kinase inhibitors, or radiotherapy (except palliative radiotherapy to known metastatic sites as long as it does not interfere with tumor imaging evaluation). Palliative radiotherapy to target lesions is not allowed.

Note: Concomitant use of hormones for non-cancer related conditions (e.g., insulin and hormone replacement therapy for diabetes mellitus) is acceptable.

3) Immunosuppressive drugs include, but are not limited to, systemic glucocorticoids at doses greater than 10 mg per day of prednisone or equivalent, methotrexate, azathioprine, and TNF-α antagonists.

NOTE: The use of immunosuppressive medications for the treatment of investigational drug-associated AE or in subjects with contrast sensitization is acceptable. In addition, inhaled, topical, and intranasal glucocorticoids are permitted. Short-term use of glucocorticoids may be permitted for the treatment of underlying or concurrent disease.

4) Immunomodulators (e.g., thymosin, interferon, interleukin-2, zearalgonkin, and shiitake polysaccharide)

5) Live or attenuated vaccines (30 days prior to the study period through Day 120 after the last dose).

6) Prior to the first Penpulimab infusion, premedication to prevent infusion-related

reactions (i.e., primary prevention) is not permitted; after the occurrence of an infusion-related reaction, any planned prophylactic medication is permitted for subsequent infusions of Penpulimab (i.e., secondary prevention).

6.8.3 Drugs and Treatments That May Be Combined During the Study Period

Patients may receive other symptomatic supportive therapy while taking study drug. Non-conventional therapeutic treatments (e.g., herbs or acupuncture) and vitamins/minerals may be used if the investigator believes there is no impact on the study endpoints. Patients may receive bisphosphonates for bone metastases during treatment. If painful bone metastatic lesions are not effectively controlled by systemic therapy or local analgesia, small-area (radiotherapy area must be <5% bone marrow area) irradiation with palliative radiotherapy is permitted. Clinical co-morbidities and the presence of various types of AE should be treated and managed aggressively. Any combination of various drugs should be recorded in the case report form (CRF) in strict accordance with GCP.

7. Study Steps

Before starting the study, the patient must read and sign the informed consent form which is currently approved by the Ethics Committee (EC). The examinations and experimental procedures will be carried out according to the time of the study flow chart, and the length of discontinuation of medication will not be accepted. However, variations in the window period of each screening program due to festivals, holidays or other administrative reasons are allowed.

7.1 Screening Period Visits

Unless otherwise indicated, the following screening steps must be completed within 4 weeks prior to initiation of study drug therapy:

- Sign the informed consent form.

- Collection of demographic information: gender, year of birth, ethnicity, height, weight, etc.
- Physical examination: head, face, dermatologic system, lymph nodes, eyes, ear, nose, and throat, oral cavity, respiratory system, cardiovascular system, abdomen, genitourinary system, musculoskeletal, neurologic system, and mental status.
- Smoking history: duration of smoking, amount of smoking (cigarettes/day).
- Tumor diagnosis and treatment history: date of diagnosis, part of the lesion (primary or metastatic), stage of disease before enrollment; first-line chemotherapy/radiotherapy regimen (time of initiation, dose of radiotherapy, duration of dosing, time of progression, optimal efficacy), reason for change of medication (if change of medication is due to progression of the disease, the imaging basis should be recorded; if change of treatment regimen is due to intolerance, the adverse effects and severity should be recorded), grade 3 or above toxicity; type of relapse.
- History of treatment for co-morbidities: e.g., diabetes mellitus, hypertension, or chronic obstructive pulmonary disease.
- Blood pressure monitoring: Blood pressure was measured by the investigator during the screening period; each blood pressure measurement was taken from the same side of the body.
- Imaging examination: PET-CT was used uniformly. Patients with stable brain metastases are required to confirm the absence of brain hemorrhage symptoms within 4 weeks of enrollment.

Remarks: According to the criteria of RECIST version 1.1, if there is more than one measurable lesion at the time of baseline evaluation, all lesions should be recorded and measured, and the total number of lesions should not be more than 5 (not more than 2 per organ), for example, patients with only one or two cumulative organs should select at most 2 or 4 target lesions as the baseline measurement

lesions.

Target lesions must be selected based on size (longest diameter), be representative of all involved organs, and measurements must be well reproducible. When the largest lesion is not reproducible, the largest lesion that is reproducible may be re-selected.

Measurable lymph nodes must meet the following criteria: a short diameter of ≥15 mm measured by CT. baseline only To detect the short diameter, the node is usually judged to have existing tumor metastasis with the help of the short diameter of the node, and the size of the node is usually represented by two-dimensional data from imaging tests (CT uses the axial plane, and MRI selects a plane from the axial, sagittal, or coronal planes), and the smallest value is taken as the short diameter. For example, a 20 mm × 30 mm abdominal nodule with a short diameter of 20 mm could be considered a malignant, measurable nodule. In this example, 20 mm is the measurement of the nodule. Nodules ≥10 mm but <15 mm in diameter should not be considered as target lesions, whereas nodules <10 mm do not fall into the category of pathological nodules and do not need to be recorded and further observed.

The calculated sum of the diameters of all target lesions (including the longest diameter of non-nodular lesions and the shortest diameter of nodular lesions) will be reported as the sum of the baseline diameters. If lymph node diameters are included, only the short diameters will be counted as mentioned above. The sum of the baseline diameters will be used as a reference value for the baseline level of disease.

All remaining lesions, including pathologic lymph nodes, can be considered non-target lesions and do not need to be measured, but should be documented at the baseline assessment as "present," "absent," or in rare cases, "definite progression. ". Widespread target lesions may be recorded with the target organ (e.g. extensive liver metastases).

- Thyroid function: TSH, free T4, free T3.

- Tumor marker (CEA/NSE) tests.
- PD-L1 test.
- Echocardiography.
- Hepatitis B and C tests: Hepatitis B 2-half test, Hepatitis C virus antibody (anti-HCV).
- Blood pregnancy test for women of childbearing age.
- Lung function test with normal or mildly to moderately abnormal lung function (VC%>60%, FEV1>1.2L, FEV1%>40%, DLco>40%) to tolerate lung cancer resection.
- Pathologic diagnosis
- Blood pressure monitoring (patient self-recorded)
- Comorbidities: need to document comorbidities within 28 days prior to enrollment

--The following screening steps must be completed within 7 days prior to initiation of study drug therapy:

- ECOG score.
- Vital signs: heart rate, respiratory rate, temperature, blood pressure.
- Routine blood count: hemoglobin, red blood cells, white blood cells, neutrophil count, lymphocyte count, and platelet count.
- Urine: urine protein, urine glucose, urine occult blood (urine erythrocytes, leukocytes), urine pH, and urine ketone bodies. If semiquantitative methods show protein ≥2+ (eg, urine test strips), perform a quantitative 24-hour urine protein test; enrolled patients must have a quantitative 24-hour urine protein count of <1g.
- Stool routine: occult blood, if stools are reviewed+ , gastroscopy is requested.
- Blood biochemistry: total bilirubin, conjugated bilirubin, ALT, AST, total protein, albumin, urea nitrogen, creatinine, uric acid, blood glucose, potassium, sodium, chloride, calcium.
- Thyroid function: TSH, free T4, free T3.

- Coagulation function tests: PT, APTT, TT, Fbg, INR.
- Electrocardiogram: 12-lead electrocardiogram.
- Cardiac enzyme profile: one test only within 7 days before enrollment, no subsequent tests are required, and this test is supplemented only if the ECG is abnormal or if symptoms such as precordial pain or palpitations are present.

The following study steps must be performed in patients who have been screened and meet the inclusion criteria and do not meet the exclusion criteria:

- Trial drug dispensing/administration.

7.2 Treatment Period Visit

During the preoperative treatment period visit phase, the visit is performed on Cycle 1, Day 21 (C1D21) and the following steps are completed:

- Physical examination: head, face, dermatologic system, lymph nodes, eyes, ear, nose and throat, oral cavity, respiratory system, cardiovascular system, abdomen, genitourinary system, musculoskeletal, nervous system, and mental Status.
- Vital signs: heart rate, respiratory rate, temperature, blood pressure.
- ECOG score.
- Blood pressure was checked at least 3 times a week for the first 2 cycles, and followed up daily if the blood pressure was abnormal.

Blood pressure is checked 2 times a week after 2 cycles if blood pressure is normal. Blood pressure monitoring was done by the patients themselves and was recorded in the patient diary card.

The blood pressure monitoring was done by the patients themselves and recorded in the patients' diary cards. At each follow-up visit, blood pressure was measured again by the investigator.

- Recording of adverse events and concomitant medications
- Medication Adherence

at 21 days in cycle 2 (C2D21), 21 days in cycle 3 (C3D21), preoperative assessment (7 days before surgery

within), postoperative visits (3-6 weeks after surgery), visits every 12 weeks during the postoperative treatment period (within the first postoperative year), and every 24 weeks during the postoperative observation period (postoperative years 2-5), and completion of the following steps at the above visits:

- Vital signs: heart rate, respiratory rate, temperature, blood pressure.
- Physical examination (except during the postoperative observation period): head, face, dermatologic system, lymph nodes, eyes, ear, nose, and throat, mouth, respiratory system, cardiovascular system, abdomen, genitourinary system, musculoskeletal, neurologic system, and mental status.
- Blood pressure monitoring (performed only in C2D21 and C3D21): blood pressure was tested at least 3 times per week for the first 2 cycles of blood pressure, with daily follow-up if blood pressure was abnormal, and 2 times per week after 2 cycles if blood pressure was normal. Blood pressure monitoring was done by the patients themselves and recorded in a patient diary card. At each follow-up visit, blood pressure was measured again by the investigator.
- Blood tests: hemoglobin, red blood cells, white blood cells, neutrophil count, lymphocyte count, and platelet count.
- Urine routine: urine protein, urine glucose, urine occult blood (urine erythrocytes, leukocytes), urine pH, and urine ketone bodies; 24-hour urine protein quantification was performed if a semiquantitative method showed protein ≥2+ (eg, urine test strip).
- Blood biochemistry: total bilirubin, conjugated bilirubin, ALT, AST, total protein, albumin, urea nitrogen, creatinine, uric acid, blood glucose, potassium, sodium, chloride, and calcium.
- Stool routine: occult blood. If there is any abnormality, seek medical advice at any time.
- Thyroid function: TSH, free T4, free T3.

- Coagulation function test: PT, APTT, TT, Fbg, INR.
- Blood sample collection: 10mL each time
- Tumor markers: CEA, NSE.
- Electrocardiogram (performed only for C2D21, preoperative evaluation and postoperative visit): 12-lead electrocardiogram. If symptoms such as precordial pain and palpitations occur, ECG should be examined immediately, and cardiac enzyme profile should be added; additional cardiac ultrasound (LVEF) is needed for clinically significant ECG abnormalities
- Imaging: Chest CT is used to characterize and evaluate lesions, except for C3D21 and the postoperative visit where no imaging evaluation is performed. An additional abdominal ultrasound was performed within 7 days preoperatively. If new lesions are suspected they may be examined in due course; imaging beyond the planned may be performed when disease progression is suspected (e.g., worsening of symptoms).
- Pulmonary function tests (preoperative evaluation and postoperative visit only).
- Pathologic evaluation (performed at postoperative visit only) for MPR and pCR. MPR is defined as neoadjuvant

tumor specimen surgically resected after treatment with ≤10% residual tumor cells. The formula was calculated as: residual

Region of surviving tumor cells (region of residual surviving tumor cells / + region of necrosis+ region of stromal tissue).

pCR was defined as the absence of residual viable tumor cells within the tumor bed in the pathological response assessment of postoperative specimens after neoadjuvant therapy.

viable tumor cells.

- PD-L1 assay (performed only at the postoperative visit).
- ECOG score (except for postoperative observation visit).
- Adverse Events: Adverse events were recorded from the first dose of study drug until at least 21 days after the last dose and followed until the adverse event

resolved or stabilized.

- Comorbid Medications: concomitant medications and concomitant treatments during the study period. Once a subject discontinues trial treatment, only concomitant medications and concomitant treatments used for new or unresolved adverse events related to trial treatment should be recorded.
- Study Drug: Dispensing/recall of trial drug. Weekly at the end of the period. Except for preoperative assessments and postoperative visits.
- Study Drug Adherence: at the beginning of the first day of each visit cycle, drug doses, counts, and adherence for the previous visit cycle are calculated and documented in the CRF, except for preoperative assessments and postoperative visits.

7.3 Safety Follow-Up

Safety follow-up refers to the visit procedure that should be completed by the subject within 21 days of termination of treatment by contacting the patient himself/herself, his/her family, or a local physician by telephone to collect information on new AEs or follow-up of AEs experienced at the time of termination of treatment, as well as on all comorbidities including antineoplastic therapies. If possible, follow-up should be done until the SAE has an outcome. This specifically includes the following:

- Documentation of adverse events (new AE or follow-up of AE experienced at termination of therapy)
- Documentation of comorbid medications (all comorbid medications including antineoplastic therapy)

7.4 Survival Follow-up

Subjects entered follow-up at the end of the postoperative observation period, which took place every 48 weeks ± 7 days. Survival (date of death and cause of death) and study post-treatment information (including treatments

received) is collected by telephone interviews with the subject himself/herself, his/her family, or a local physician until the end-point of death or until the subject is lost to follow-up or the Sponsor terminates the study:

7.5 Unplanned Visits

Subjects may experience AEs during the course of the trial requiring unplanned follow-up visits, including documentation of the following items:

- Documentation of concomitant medications/treatments
- Documentation of concomitant medications/treatments
- Documentation of relevant investigations performed (including imaging, if any)

8. Efficacy evaluation indicators

8.1 Main indicators and observation methods

8.1.1 Assessment of main efficacy indicators

Major pathologic remission rate (MPR).

Defined as ≤10% of residual tumor cells in surgically resected tumor specimens after neoadjuvant therapy. The formula is: residual surviving tumor cell area/(residual surviving tumor cell area+ necrotic area+ stromal tissue area). See Appendix V for specific assessment forms.

8.1.2 Assessment of secondary efficacy indicators

Complete Pathologic Remission Rate (pCR).

Defined as the absence of residual viable tumor cells within the tumor bed and in the lymph nodes as assessed by the pathological response of postoperative specimens after neoadjuvant therapy. See Appendix V for specific assessment forms.

Preoperative objective remission rate (ORR):

Refers to the proportion of patients with a certain amount of tumor shrinkage that is achieved and maintained for a certain period of time, and encompasses both CR and PR cases. Objective tumor remission was assessed using the Solid Tumor Remission Assessment Criteria (RECIST 1.1 criteria). Subjects had to be accompanied by measurable tumor lesions at baseline, and the efficacy assessment criteria were classified as complete remission (CR), partial remission (PR), stable (SD), and progression (PD) according to the RECIST 1.1 criteria.

1-year event-free survival percentage (EFS%).

Defined as the proportion of subjects who did not experience imaging-confirmed disease progression, local progression leading to inoperable disease, unresectable tumor, local or distant recurrence, or death due to any cause from the time of initiation of the drug to 1 year as a proportion of the total number of subjects in the group.

Event-free survival (EFS)

Defined as the period from initiation of medication until imaging-confirmed disease progression, local progression leading to inoperable disease, unresectable tumor, local or distant recurrence, and death due to any cause.

Overall Survival (OS):

Overall survival (OS) was defined as the period from the date of enrollment to the date of death from any cause. Subjects who were alive at the time of the final follow-up visit had an OS that was data censored at the time of the final follow-up visit. The OS of subjects who were lost to follow-up was counted as data censored at the time of last confirmed survival prior to the loss of follow-up. OS for data deletion was defined as the time from enrollment to deletion.

For safety (SAFETY) evaluation, refer to Section 9.

9 Safety evaluation

9.1 Adverse events

Adverse Event (AE) refers to all adverse medical events that occur after a subject receives a test drug, which may manifest as signs and symptoms, disease, or abnormal laboratory tests, but are not necessarily causally related to the test drug.

The collection period for AEs in this trial begins when the subject signs the informed consent form and continues until 30 days after the last dose of the drug or the initiation of treatment for a new target indication.

9.2 Evaluation of Adverse Events

Criteria for evaluating the nature and severity of adverse events followed the National Cancer Institute's Common Toxicity Criteria [NCI -CTC v5.0].

9.3 Documentation of Adverse Events

It is the responsibility of the investigator to collect all AEs (including SAEs) during the protocol-specified AE reporting period and record them on the CRF/eCRF form. When recording AEs, the investigator should use correctly standardized medical terminology and avoid colloquialisms and abbreviations. The time of onset of the AE, the NCI CTCAE v5.0 grading of the highest degree, the time of termination, relevance to the study medication, impact to the study, presence or absence of concomitant therapy, and recovery need to be documented.

Diagnosis vs. signs and symptoms

If a diagnosis already exists, the diagnosis should be recorded on the CRF/eCRF rather than individual signs and symptoms (e.g. record liver failure rather than jaundice, elevated aminotransferases and fluttering tremor). However, if the symptoms and signs cannot be categorized as a single diagnosis at the time of reporting, each individual event should be recorded as an AE on the CRF/eCRF. If the diagnosis is later established, it should be updated on the CRF/eCRF to document the diagnosis.

Adverse events secondary to other events

In general, AEs secondary to other events (e.g., caused by other events or clinical sequelae) should be documented as primary events unless the secondary event is of greater severity or is an SAE; however, clinically significant secondary events should be documented as separate AEs on the CRF/eCRF if they do not occur at the same time as the primary event, or separately if the association between the events is not clear. record the primary event and the secondary event.

Persistent, intermittent, or separate adverse events (frequency of adverse events)

A persistent AE (continuous AE) is an AE that persists throughout the course without remission, e.g., an upper respiratory infection that lasts 5 days. This type of AE should be recorded with only one entry on the CRF/eCRF. For severity assessment, the most severe of the event over the full course should be recorded.

An intermittent AE is an AE in which symptoms, signs, or laboratory markers change or resolve throughout the course of the event, but no clinically significant outcome occurs, e.g., nausea and vomiting that lasts for multiple days, with relative remission during the course of the event, or hypertension in a patient who has had intermittent remissions in multiple blood pressure tests, but the course of the hypertension has been relatively continuous. Such AEs may be recorded as a single event on the CRF/eCRF. For intensity assessment, the most severe of the full course of the event should be recorded.

A single AE (Single AE) is an AE that logically can only occur in isolation or that occurs independently only once during the trial, e.g., an incidental fall of the patient during medication administration; vomiting by the patient that occurs only once during the trial. Such AEs are recorded only once on the CRF/eCRF.

It should be noted that if there has been a clinically significant recovery from the above AE, but the same AE occurs later and the latter is not considered to be in continuity with the former in terms of course, the occurrence of the event should be recorded separately on the CRF/eCRF.

Abnormal laboratory tests or abnormal vital signs

All laboratory test results may be documented on the CRF Laboratory Results page. Not all laboratory test abnormalities/abnormal vital signs should be documented as an AE. It is the responsibility of the investigator to review all abnormal laboratory results and abnormal vital signs and make a medical judgment as to whether they should be documented as an AE. Any of the above abnormalities should be documented as an AE when clinically significant, e.g., when one or more of the following conditions are met:

- Accompanying clinical symptoms
- Result in a change in study medication (e.g., dose adjustment, temporary or permanent discontinuation)
- Requires medical intervention or change in combination therapy (e.g., addition, suspension, discontinuation, or other change in combination medication, treatment, or

therapy)

- Clinically significant in the judgment of the investigator

If clinically significant laboratory test abnormalities or vital sign abnormalities are symptomatic of a disease or syndrome (e.g., elevated ALT/AST and blood bilirubin due to hepatic impairment), record the diagnosis (i.e., hepatic impairment) only on the Adverse Events form of the CRF/eCRF. Conversely, record an abnormal laboratory test or abnormal vital signs on the Adverse Events form of the CRF/eCRF and indicate whether the test value is above or below the normal range. If the abnormal laboratory test or abnormal vital sign has a standard clinical term associated with it, the clinical term should be recorded on the CRF/eCRF (e.g., an elevated potassium level of 7.0 mmol/L should be recorded as "hyperkalemia").

Death

When recording a death, if there is an AE that led to the death, record the AE that led to the death using a single medical concept in the CRF/eCRF and report the event as an SAE; if the cause of the death is unknown, record the "Unexplained Death" in the AE table of the CRF/eCRF and report the "Unexplained Death" as an SAE first. If the cause of death is unknown, record "Unexplained Death" on the AE form of the CRF/eCRF and report the event as an SAE, then investigate the exact cause of death and update the record and SAE report when the cause of death is known.

Pre-existing medical conditions

Symptoms/signs that were present during the screening period of the trial should be recorded as AEs only if there is an exacerbation of the severity, frequency, or nature of the symptoms/symptoms (other than a worsening of the disease condition under study) after entry into the trial. Changes in status relative to previous status should be documented, e.g., "increased frequency of headache," "worsening of hypertension," etc.

Hospitalization, prolonged hospitalization

A hospitalization or prolonged hospital stay should not be reported as an SAE if it is: 1) planned in accordance with protocol requirements (e.g., for medication administration, efficacy assessment, etc.); or 2) due to a pre-existing medical condition that has not changed since participation in the study, such as elective surgery or treatment scheduled prior to participation in the study, and then

hospitalized after participation in the study to receive the surgery or treatment, if this is not considered to be an adverse event. considered an adverse event. However, if the condition of a pre-existing medical condition worsens in the study (e.g., surgery or treatment is performed earlier than originally planned), then hospitalization for surgery or treatment due to the worsening of the medical condition will require hospitalization for surgery or treatment, and the worsening of the condition will be considered an SAE.

Surgery

If the condition for which the surgery was performed is clearly identified, the condition should be recorded as the AE, not the surgery itself (e.g., if the patient underwent an inguinal hernia repair, "inguinal hernia" should be recorded, not "inguinal hernia repair"); however, if the reason for the surgery is not clear, the name of the surgery may be recorded as the AE. However, if the reason for the procedure is unclear, the procedure name may be recorded as AE (e.g., if the patient underwent an exploratory laparotomy, "exploratory laparotomy" may be recorded as AE).

Pregnancy

The Investigator should be notified immediately if a pregnancy occurs in a female subject or a female partner of a male subject during the clinical trial. The investigator should report the pregnancy to the sponsor by completing the Serious Adverse Event Report Form within 24 hours of learning of the pregnancy event, and follow-up should continue until the pregnancy is terminated (e.g., termination of pregnancy, labor and delivery) and the results reported to the sponsor. If a female subject becomes pregnant, the investigational drug should be discontinued immediately and the investigator should discuss with the subject the risks of continuing the pregnancy and the possible effects on the fetus.

In the event of an induced/spontaneous abortion, termination of pregnancy for medical reasons, or congenital anomalies or malformations of the fetus/neonate during pregnancy, this is considered an SAE and will need to be documented and reported in accordance with the timeframe requirements for SAEs.

Disease progression

If an event occurs that is unequivocally consistent with the expected pattern of progression of the primary tumor, it should not be considered an AE. hospitalization due solely to the progression of that disease should also not be considered an SAE.

if the symptoms are not unequivocally due solely to the progression of the disease or do not coincide with the expected pattern of progression of the tumor, the associated clinical symptoms may be documented as an AE, and a SAE should be reported if it is consistent with an SAE.

9.4 Follow-up of adverse events

Investigators should follow all AEs until any of the following occurs.

- The AE resolves or improves to baseline levels;
- The investigator confirms that no further improvement is expected;
- The patient dies;
- The patient has been lost to contact;
- The investigator confirms that the AE is not related to the study treatment;
- The patient begins a new anticancer treatment;
- Clinical or safety data are no longer being collected, or the database is finally closed.

The final outcome of each AE (including the date of AE remission or death) must be recorded on the CRF/eCRF.

9.5 Criteria for determining drug-adverse event association

The investigator should make an assessment of the possible association between the adverse event and the test drug, which can be determined in the manner listed in the table with reference to the following five categories of criteria.

(1) Whether there is a reasonable chronological sequence between the administration of the drug and the appearance of the adverse reaction;

(2) Whether the reaction is consistent with the types of adverse reactions known to occur with the drug;

(3) Reduction or disappearance of the reaction after discontinuation or reduction of the drug;

(4) Whether the same reaction recurs after reuse of the suspected drug;

(5) Whether the reaction can be explained by the effects of the combined drug,

the progression of the patient's condition, or other therapeutic measures;

Table 10.5.1 Adverse Event-Drug Relationship Determination Forms

	1	2	3	4	5
Definitely relevant.	+	+	+	+	-Likely.
Most likely.	+	+	+	Most likely.	-Likely?
Possibly.	+	-Possibly.	±Possibly.	What's going on?	±?
Maybe not.	-It's probably not relevant.	-It's probably not.	±What's the matter?	What? - What?	±?
Definitely not.	-I'm sure it's not.	-It's not.	It's definitely not--	-It's not.	-

Note: + means yes, - means no,± means difficult to confirm or deny, ? means the situation is unknown.

Adverse reactions were counted as those that were definitely related, probably related, probably related, or probably unrelated, and the incidence rate of adverse reactions was calculated accordingly.

9.6 Serious adverse events

Adverse events are classified as serious adverse events when one or more of the following criteria are met: death, life-threatening (e.g., immediate risk of death), resulting in hospitalization or prolonged hospitalization, permanent or severe disability, congenital deformity or defect, and some medical events that do not result in death, life-threatening injury, or hospitalization, but which are judged by the physician to be potentially harmful to the patient or to require medication or surgery, are also considered to be serious adverse events. A medical event that, in the judgment of the physician, may be harmful to the patient or require medication or surgical intervention to avoid the above is also considered a serious adverse event. The occurrence of pregnancy in the patient or his/her spouse is reported as a Serious Adverse Event to the relevant unit at .

Disease progression (including signs and symptoms of progression) should not be reported as a Serious Adverse Event, but death due to disease progression within the trial or safety reporting period should be reported as a Serious Adverse Event.

Hospitalization for signs and symptoms of disease progression should not be reported as a serious adverse event. If the final outcome of the cancer is death during the trial or safety reporting period, then the event leading to death must be reported as a Serious Adverse Event.

9.7 Handling of Serious Adverse Events

Any Serious Adverse Event that occurs during the course of a clinical trial should be reported in writing by the investigator to the sponsor's Pharmacovigilance Department at the designated email address (AL3818@cttq.com) within 24 hours of notification, and should be followed by a timely, thorough, written follow-up report. For reports involving fatal events, the investigator should provide the sponsor and the ethics committee with other required information such as autopsy reports and final medical reports. Serious adverse events should be analyzed and evaluated immediately upon receipt by the sponsor, including severity, relevance to the test drug, and whether it is an expected event. For suspected and unanticipated serious adverse events, the sponsor should promptly report them to all investigators participating in the clinical trial of the investigational drug, as well as to the clinical trial site and the ethics committee; and the investigators should report suspected and unanticipated serious adverse events provided by the sponsor to the ethics committee. The sponsor should also report suspected and unanticipated serious adverse reactions to the drug regulatory authorities and health authorities.

9.8 management of common adverse events

6.5.1 The section provides dosing adjustments and recommendations for some common adverse events of amlotinib hydrochloride capsules. The types of immune-related adverse events triggered by Penpulimab injections and recommendations for treatment can be found at Recommendations for the Management of Immune-Related Adverse Events Caused by Immune Checkpoint Inhibitor Therapy .

10. Data Management

The purpose of data management is to ensure that data are reliable, complete and accurate. The goal is to obtain real data of high quality for statistical analysis. In

this study, an electronic case report form (eCRF) will be used for study data collection and management.

10.1 Data Collection

This study will use eCRF for study data collection; Chia Tai Tianqing Pharmaceutical Group Co. will provide electronic data collection (EDC) system to the research organization. Ltd. will provide the electronic data capture (EDC) system to the research organization. The company staff will train the designated research organization personnel on the EDC system. Site personnel will be trained to log on to the EDC system. the PI or dedicated data entry personnel (CRC) should enter data into the EDC system according to the requirements of the visit process and the guidelines for completing the eCRF. The system logic verification program will check the integrity and logic of the clinical trial data entered into the EDC system and generate error message alerts for problematic data, allowing the PI or CRC to modify or interpret the problematic data. After the database is locked, the investigator receives a copy of the patient data on CD-ROM or file for archiving at the site. CZT is the sole owner of the original completed eCRF and is not permitted to make it available to third parties in any form without the written permission of CZT, except for CZT or an authorized representative of the regulatory agency. The Investigator is ultimately responsible for the collection and reporting of all clinical and laboratory data recorded in the eCRF and other data collection forms (original records) to ensure the attributability, legibility, timeliness, originality, accuracy, permanence, completeness, and consistency of such records.

The eCRF must be confirmed by the signature of the investigator or relevant authorized person to verify that the data recorded in the eCRF are genuine. Any data corrections in the eCRF and in the original record must be dated, signed by name, and given the necessary explanation, but must not obscure the previous original record.

Typically, the original record is the hospital or physician's chart. At that point,

the data collected in eCRF must be consistent with the data from those charts. In some cases, the eCRF may also serve as the original record. In this case, the research organization will need to have documentation that specifies which data will be recorded in the eCRF and that the eCRF will be used as the original record.

10.2 Data Management and Quality Control

To ensure that the clinical trial data are true and reliable and to improve the quality of clinical data, the clinical supervisor will review the trial data in the clinical database for completeness, consistency, and accuracy in accordance with the standard operating procedures during the course of the trial program, and will instruct the study site personnel to make any necessary additions or corrections to problematic data. The clinical supervisor or data manager will challenge questionable data in the form of an electronic challenge form to the PI or CRC, who must respond to the challenge and make corrections or explanations to the questionable data, and if necessary, issue multiple challenges until the questionable data is resolved. The medical director and data manager conduct periodic concordance comparisons of SAEs.

At the end of the trial project, the data manager and medical staff will perform a final quality control of all data in the database, summarizing all protocol offsets and protocol violations that occurred during the conduct of the trial, and convene a data verification meeting. Once the data in the database have met the quality requirements, the database will be locked and the trial unblinded, and the data manager will export the data for data analysis by the statistical department.

10.3 Review of data and monitoring of research institutions

Prior to trial initiation, during the initial visit to the study site or investigator meeting, a representative of Chia Tai Tianqing Pharmaceutical Group Co., Ltd. will work with the investigator and staff to introduce the trial protocol and eCRF. During the trial period, the supervisor will visit the study center on a regular basis to check

the completeness of the patient records and the accuracy of the content filled out on the eCRF, the adherence to the trial protocol and the quality management standard for drug clinical trials, progress of enrollment, and to ensure that trial medications are stored, dispensed, and counted in accordance with regulations. Key investigators must be available to assist the monitors during these visits.

The investigator must maintain the original documentation for each patient enrolled in the trial, including study charts and visit notes (inpatient or outpatient medical records), which include demographic indicators and medical information, laboratory data, electrocardiograms, and results of any other tests or evaluations. all information on the eCRF must be derived from the original documentation in the patient's file. The investigator must also maintain the informed consent form signed by the patient.

The investigator must confirm that all relevant original documents can be monitored to verify that they are consistent with the content of the eCRF. The monitoring criteria of the eCRF require that 100 percent of the monitoring has been done to obtain informed consent, compliance with inclusion/exclusion criteria, documentation of SAEs, and all data necessary for the evaluation of the primary and safety metrics. Additional checks for consistency between the raw data and the eCRF are performed in accordance with the monitoring plan specified for the trial. Any information on patient identity in the raw files will not be disclosed.

10.4 Retention of trial records

In order to satisfy the requirements for review and/or audit by the regulatory authorities or CTTQ, the investigator/institution agrees to maintain relevant records, including all subject identification numbers (sufficient information to be linked to the records, e.g., eCRF and hospital records), all original signed informed consents, all copies of the eCRF, safety report forms, original records, records of details of treatment treatment treatments, relevant communication documents (e.g. letters, meeting minutes, telephone reports). The investigator/institution is required to

maintain records in accordance with the relevant regulatory requirements.

The investigator/institution should notify CTTQ in advance if they can no longer keep the study records for any reason. Study records should be transferred to a recipient designated by CTTQ, such as another investigator, another organization, or an independent third party arranged by CTTQ. The study data should be retained by the research organization until 5 years after the completion of the clinical trial and by the co-sponsor until 5 years after the product has been marketed. Even after the expiration of the retention period, the Investigator must obtain written permission from CTTQ for the disposal of any records of this trial. When the data do not need to be retained any longer, CTTQ will promptly notify the Investigator/institution.

10.5 Data Processing

When the clinical trial is completed, data processing is commissioned by the sponsor to a data statistics company. Inconsistent information is identified through data verification. Any inconsistent information should be clarified by contacting the investigator through the clinical supervisor. When the data in the database is considered correct, it will be analyzed and statistically analyzed.

11 Statistical analysis

The main efficacy index of this study is the MPR, which was analyzed after the last subject had completed the post-surgical tumor tissue sections and obtained the results, and the 95% confidence intervals of the MPR were estimated by the Clopper-Pearson method.

11.1 Selection of data for statistical analysis

- Full Analysis Set (FAS set): according to the principle of Intentional Analysis (ITT), the efficacy of all cases who took 1 dose of the drug was analyzed. For case data where the full course of treatment could not be observed, the last observation data was used to carry forward to the trial final result (LOCF).

- Per-protocol Set (PPS Set): all cases that complied with the trial protocol, were compliant, and took at least 2 cycles of medication (subjects who took more than one cycle of medication and had clear imaging evidence of disease progression were also included in the PPS Set), did not take prohibited medications during the trial period, and completed the required fields on the case report form. Missing data were not filled in (imputation). The efficacy of the drug was statistically analyzed for both FAS and PPS.
- Safety Analysis Set (SAS set), all enrolled cases, all patients who have used the trial drug at least once and have a record of the safety of the drug after use belong to the safety analysis set, this data set is used for safety analysis.

11.2 Principles and contents of statistical analysis

(1) General principles

All statistical analyses were realized by writing programming language using SAS version 9.4 statistical software, and all statistical tests were conducted using two-sided tests, where a P value of ≤0.05 would be considered statistically significant for the differences tested, and the confidence interval was used with a 95% confidence level.

Baseline data were analyzed by the full analysis set, safety analyses were analyzed using the safety analysis set, and validity analyses were analyzed by the full analysis set and the compliance with the protocol set.

For continuous variables, the number of non-missing subject cases, mean, standard deviation, median, minimum and maximum values will be listed. The number of decimal places for the minimum and maximum values will be consistent with the records in the database. The mean, median, and standard deviation will retain one more decimal place than the original data recorded in the database.

For categorical variables, they will be presented in the form of frequency tables (frequencies and percentages). Percentages will be retained to two decimal places.

Estimation of missing values: no estimation of missing values will be made in

the safety evaluation. If the efficacy evaluation is missing due to early withdrawal from the treatment, "unable to evaluate" will be used to participate in the analysis of the objective remission rate and disease stabilization rate, and the missing OS and PFS will be used as the truncated data.

(2) Study population

①Population distribution

Descriptions of the enrollment status, dropout cases, completion of the expected course of treatment, and early discontinuation of the study population: number and percentage of cases. Distribution of cases, statistical description of dropout and exclusion cases one by one: medication use, proposed reasons for withdrawal from the trial, etc.

Distribution of cases in the safety dataset.

Case-by-case descriptions of dropout and early discontinuation cases: medication use, reasons for early withdrawal, etc.

②Protocol violations and deviations

Summarize and describe cases of protocol violations and deviations.

③ monitoring population demographic characteristics, disease characteristics and medical history analysis

(3) Effectiveness analysis

The primary study endpoint of this study was the MPR in the full analysis set (FAS) population as assessed according to RECIST v1.1. The analysis of the primary endpoint was performed after the last subject had completed the post-surgical tumor tissue sectioning and obtained the results. 95% confidence intervals for MPR were estimated using the Clopper-Pearson method.

Subjects' tumor tissue section pCR as well as 1-year event-free survival percentage (1-y EFS%), objective remission rate (ORR), event-free survival (EFS), safety, and overall survival (OS) based on the investigator's assessment were secondary efficacy metrics for this study. Among them, for the analysis of EFS and OS, the Kaplan-Meier method will be used to estimate the median time and the

corresponding 95% confidence intervals will be listed.The analysis of ORR and pCR will be done using the Clopper-Pearson method to estimate the 95% confidence intervals.

Comparisons with baseline check values will be made using paired t-tests or Wilcoxon signed rank sum tests, depending on the distributional characteristics of the information

(4) Safety evaluation

Safety analysis is mainly based on descriptive statistical analysis methods, and confidence intervals are utilized to present relevant results when necessary.

①Adverse events

Adverse event data, coded according to the current version of MedDRA at the time of the start of coding, will be processed in the statistical analysis.

Summarizes the incidence of adverse events, adverse reactions, adverse events leading to withdrawal, adverse events leading to death, serious adverse events, etc. Incidence of adverse events/reactions= Number of adverse events/reactions in the number of medications administered/number of medications administered x 100%. If multiple adverse events/reactions occurred in the same monitored case, it was recorded as 1 case in the calculation of the incidence rate; when the same AE occurred multiple times in a monitored case, it was recorded as 1 case in the calculation of the incidence rate of that AE.

Adverse events will be summarized in the frequency table by System Organ Classification (SOC) and Preferred Terminology (PT). Calculate the incidence rate by system and signs/symptoms (count of cases: number of monitored cases with at least one occurrence of a particular adverse event).

Severity of adverse events, reactions: multiple occurrences of the same adverse event in the same subject, with the most severe one participating in the severity analysis for that AE.

Provide a list of cases with the time of occurrence of the adverse event and the time of the serious adverse event.

(ii) Vital signs, laboratory test data, etc;

Mean ± standard deviation, maximum, minimum, and median were used to describe the measurements and changes before and after treatment. Vital signs and laboratory indicators were analyzed descriptively, and paired t-tests were used for within-group comparisons.

(iii) Drug exposure and dose adjustment

Mean, standard deviation, maximum, minimum and median were used to describe the amount of drug administered, and dose adjustment and suspension were analyzed.

④Combined medications

The use of combined medications during the study period was summarized, and the frequency of use of each medication.

12. Laws, regulations, ethics and informed consent

12.1 Laws and regulations

Prior to the initiation of the study, the study must be approved by the appropriate regulatory authorities or filed for record before the clinical study can be conducted. The clinical study will also be conducted in compliance with all applicable regulatory requirements.

12.2 Ethics

This clinical trial must be conducted in accordance with the Declaration of Helsinki (1996), the Guidelines for the Conduct of Clinical Trials of Pharmaceuticals (GCP) issued by the NMPA, and related regulations.

12.3 Ethics Committee

This trial protocol needs to be reviewed and approved by the ethics committee of each central hospital before implementation. During the deliberation of the Ethics

Committee, the sponsor and the investigator shall provide the relevant clinical trial documents to the Ethics Committee, including the following but not limited to: "Application to the Ethics Committee", "Clinical Research Approval" issued by NMPA, "Clinical Research Commissioning Letter", "Clinical Trial Study Team Members", "Quality Inspection Report of the Drugs Used in the Trial", "Sample of Informed Consent", "Clinical Trial Protocol" and "Clinical Case Report Form (CRF)". Case Report Form (CRF)", "Investigator's Manual" for clinicians' reference and other documents. Any modification of the trial protocol during the clinical study should be reported to the Ethics Committee for record.

12.4 Informed Consent

Subjects must give informed consent to participate in this trial before receiving treatment in order to protect the legitimate rights and interests of the subjects. It is the responsibility of the investigator to provide the subjects or their designated representatives with a complete and comprehensive description of the purpose of the study, the effects of the drug, the possible toxic side effects and the possible risks, and subjects should be made aware of their rights, the risks to be borne and the benefits. The conversation is very important to the informed consent process. If the subject and his/her legal representative are not literate, the informed consent process should be attended by a witness, who should sign the informed consent form after verbal consent is given by the subject or his/her legal representative, and the witness's signature should be on the same day as the subject's signature. The informed consent form should indicate the version and date of development or date of modification.

12.5 Confidentiality of Subject Information

Every effort will be made to protect the privacy of all subjects during the course of this study, and the names of subjects shall not be included in the co-sponsor's forms, study reports, publications, and any other publicly available materials, unless required by law. This study will utilize highly confidential methods

of data transmission in order to protect the subjects' personal information from disclosure.

13. Study Administration

13.1 Study Management Organization

This study is planned to be conducted at Tianjin Cancer Hospital. The clinical supervision of this study will be conducted by Chia Tai Tianqing Pharmaceutical Group Co. The third party will be responsible for the statistical analysis.

13.2 Standardized operation

The sponsors comply with the provisions of GCP (2003 version) and drug registration management methods, and the test drugs are prepared in GMP-compliant workshops and subject to strict quality checks. Laboratory checks are conducted by each test center in accordance with standard operating procedures (SOP). Laboratory inspection methods and quality control should be unified in each test center. The clinical laboratory of each test center shall carry out indoor quality control in accordance with the regulations and obtain the certificate of quality evaluation of the clinical test center of the Ministry of Health.

13.3 Training

According to the principles of GCP, clinical supervisors should have the qualifications recognized by the sponsoring organization; before the commencement of the clinical trial, the person in charge of each trial center shall train the investigators on the trial protocol, familiarize themselves with the content of the clinical trial protocol, master the principles of GCP, standardize the recording methods and judgment standards, and strictly follow the protocol.

13.4 Clinical Supervision

Supervisors must follow the Good Clinical Practice (GCP) and Standard Operating Procedures (SOP), visit the research unit for clinical supervision on a regular basis or in accordance with the actual situation, supervise the conduct and progress of the clinical trial, check and confirm that the recording and reporting of all the data and the input of the case report form are correct and complete, and that they are consistent with the original data, so as to ensure that the clinical trial is carried out in accordance with the protocol of the clinical trial. The investigator shall actively cooperate with the work of the supervisor. The specific content of the monitor includes:

1) Confirming before the trial that the trial undertaking unit has appropriate conditions, including staffing and training, laboratory equipment is complete and in good working order, with a variety of trial-related inspection conditions, the estimated number of subjects is sufficient, and the participating researchers are familiar with the requirements of the trial protocol;
2) Monitor the investigator's implementation of the trial protocol during the trial, confirm that informed consent was obtained from all subjects prior to the trial, understand the enrollment rate of subjects and the progress of the trial, and confirm that the enrolled subjects are qualified;
3) Confirmed that all data were recorded and reported correctly and completely, and that all case report forms were entered correctly and agreed with the original data. All errors or omissions were corrected or noted, signed and dated by the investigator. Dosage changes, treatment changes, comorbidities, intercurrent illnesses, missed visits, and missed tests should be identified and documented for each subject. Verify that withdrawals and lost visits of enrolled subjects are accounted for on the case report form;
4) Confirm that all adverse events are recorded, and that serious adverse events are reported and recorded within a specified time frame; verify that trial medications are supplied, stored, distributed, and withdrawn in accordance with

relevant regulations, and recorded accordingly;

5) There should be clear and truthful records of visits that the investigator failed to make, tests that were not performed, inspections that were not done, and whether corrections were made for errors and omissions;

6) Completing a written monitoring report after each visit, which should state the date and time of the monitoring, the name of the monitor, and the findings of the monitoring.

13.5 Recording and Retention of Study Data

In accordance with the principles of GCP, the investigator shall keep all the detailed original documents of the subjects and record in the case report form about the trial process, medication, laboratory test data, safety data and efficacy evaluation, etc. The recorded data shall be guaranteed to be complete, timely and clear. The original documents, medical records etc. should be clear, detailed and easily recognized by the participants of this clinical trial.

The case report form and original file can only be modified by the investigator. Any modifications to the case report form and original file must not obliterate the original data. The correct method of modification is to draw a single line through the original data, write the modified data next to the original data, and sign the date and initials of the person who made the modification.

The test data should be retained until 5 years after the completion of the test, after which time it will be retained by the sponsor. The sponsor will retain the trial data until at least 2 years after approval of the marketing application for the trial drug or until at least 2 years after formal cessation of development of the trial drug.

If the Principal Investigator transfers or retires, or ceases to fulfill his/her research responsibilities, the Sponsor must be notified so that appropriate measures can be put in place with respect to the trial data.

13.6 Quality Control and Quality Assurance

- The clinical research unit must be a drug clinical research base with clinical research conditions determined by the State Drug Administration;
- Researchers must be physicians trained in clinical trials and work under the guidance of senior professionals;
- The clinical wards must be inspected before the trial to meet the standardized requirements and ensure that the resuscitation equipment is complete;
- The subjects are given medication by professional nursing staff, with a detailed understanding of how the medication is being taken, to ensure the subjects' compliance;
- Each research center must strictly follow the study protocol and truthfully fill in the case observation form;
- Supervisors should follow the standard operating procedures to supervise the conduct of clinical trials, confirm that all data are recorded and reported correctly and completely, and that all case report forms are filled out correctly and are consistent with the original information to ensure that the trials are carried out in accordance with the clinical study protocol;
- In the event of an SAE, the supervisor shall promptly notify each research unit and temporarily suspend the study if necessary;
- Each research unit involved in the trial shall accept the auditing by the sponsor and the drug regulatory authority, and it is particularly important that the investigator and his/her related personnel shall provide convenience and time for the monitoring and auditing.

14. Publication of study results

The ownership of the study results belongs to Chia Tai Tianqing Pharmaceutical Group Co. Zhengda Tianqing does not restrict the investigator from publishing any information collected or generated, whether or not the results are favorable to the study drug. However, in order to prevent inadvertent disclosure of confidential information or unprotected inventions, the Investigator should provide the Co-Organizer with the opportunity to review any proposed publication or other form of

release before the document is submitted for publication or released. The Investigator should provide CZT with the original manuscript, abstract, or full text of any proposed publication (poster, invited or guest lecture) at least 30 days prior to the submission of the document for publication or other form of release. If a patent is required to protect intellectual property rights, the researcher should agree to delay publication for no more than 60 days. Prior to public release, the investigator may be asked to remove any previously unpublished confidential information (other than study results). If the study is part of a multicenter study, the investigator will need to agree that the first publication will be the combined results of all study centers. However, if the original manuscript of the composite analysis has not been submitted for publication within 12 months of completion or termination of the study at all Study Centers, the Investigator may independently publish the results in accordance with the other requirements of this section.

15. Clinical Study Progress

First subject enrollment: 03/2021;

Anticipated enrollment of the last subject: 06/2022;

Anticipated study end date: December 2028;

16. Trial protocol modification and approval

The study protocol, determined by the principal investigators participating in this clinical trial after joint discussion, will be implemented after the consent of Chia Tai Tianqing Pharmaceutical Group Co. and the approval of the Ethics Committee of the study lead unit. During the clinical research trial, any modification of the trial protocol should be reported to the Ethics Committee for approval or filing.

17. Research Unit

17.1 Investigators

1) Trained in GCP and the protocol of this trial and have time to conduct this

trial according to the study protocol.

2) Patients should be provided with detailed information about the study before enrollment, and their consent should be obtained and an informed consent form should be signed.
3) The investigator is obliged to take the necessary measures to safeguard the safety of the patients. In the event of an adverse reaction, the investigator will immediately follow the relevant regulations and report it to the principal investigator. Follow-up of serious adverse reactions.
4) Fill out the study medical record in a timely manner;
5) Actively cooperate with the regular visits of the clinical supervisor;
6) Complete retention of records of laboratory tests, clinical records, and original patient medical records;

17.2 Co-sponsors

1) Provide information and other support to the investigator, and explain to the investigator the protocol and the completion of various information before clinical initiation;
2) Dispatch of clinical supervisors for regular visits;
3) The supervisor ensures that he/she is able to keep in touch with the investigator at all times, using telephone, fax and e-mail.
4) The supervisor supervises the investigator to follow the approved protocol to carry out the clinical study, verifies that the drugs used in the study are issued and recalled in accordance with the relevant regulations, and ensures that the trial records in the clinical trial are consistent with the data in the original report.

Annex I Physical Condition Scoring Criteria (ECOG)

(Eastern Cooperative Oncology Group)

Activity Score	Description
0	Asymptomatic, fully active, & able to perform unrestricted activities.
1	Symptomatic, fully ambulatory, but limited in heavy physical activity, and able to perform light or sit-based tasks such as light housework and office work.
2	Symptomatic, able to walk, self-care, but unable to perform any physical activity, awake about 50% of the time or more (when bedridden during the day< 50%)
3	Symptomatic, limited self-care, bed or chair bound >50% of waking hours, but not yet bedridden.
4	Total loss of function, totally unable to care for self, bedridden.
5	Death.

Annex II Classification of cardiac insufficiency

Classification of cardiac insufficiency according to the Ninth Revision of the New York Heart Association (NYHA), 1994.

(a) Cardiac class I: Heart disease, but physical activity is not limited and general physical activity does not cause excessive fatigue, palpitations, dyspnea, or angina pectoris. (for the period of cardiac function compensation);

(② Cardiac function class II (mild): suffering from heart disease, physical activity is slightly restricted, no symptoms when resting, feeling comfortable, but general physical activity causes fatigue, palpitations, dyspnea or angina pectoris. (I ° heart failure);

(③) Heart Function Grade III (Moderate): Suffering from heart disease, physical activity is greatly restricted, asymptomatic at rest, still feels comfortable, but general light physical activity causes fatigue, palpitations, dyspnea or angina pectoris. (II° heart failure);

(iv) Heart Function Grade IV (Severe): Suffering from heart disease, with complete loss of physical ability, symptoms of heart failure or angina may still be present at rest, i.e., dyspnea and fatigue, and the symptoms are aggravated by any physical activity. I.e., light activity can worsen dyspnea and fatigue (III° heart failure)

Annex III Creatinine clearance calculation

Cockcroft-Gault formula for calculating creatinine clearance

Serum creatinine concentration (mg/dL).

$$\text{Male creatinine clearance (mL/min)} = \frac{(140 - \text{age}) \times (\text{weight})^{a}}{72 \text{ x serum creatinine}}$$

$$\text{Female creatinine clearance (mL/min)} = \frac{0.85 \times (140 - \text{age}) \times (\text{body weight})^{a}}{72 \text{ x serum creatinine}}$$

Serum creatinine concentration (μ mol/L).

$$\text{Male creatinine clearance (mL/min)} = \frac{(140 - \text{age}) \times (\text{body weight})^{a}}{0.81 \text{ x serum creatinine}}$$

$$\text{Female creatinine clearance (mL/min)} = \frac{0.85 \times (140\text{-age}) \times (\text{body weight})^{a}}{0.81 \times \text{serum creatinine}}$$

a Age is in years and weight is in KG.

Appendix IV Eighth Edition Lung Cancer Staging Table

From CSCO Guidelines for the Treatment of Non-Small Cell Lung Cancer 2020 Edition

	N0	N1	N2	N3
T1a	ⅠA1	ⅡB	ⅢA	ⅢB
T1b	ⅠA2	ⅡB	ⅢA	ⅢB
T1c	ⅠA3	ⅡB	ⅢA	ⅢB
T2a	ⅠB	ⅡB	ⅢA	ⅢB
T2b	ⅡA	ⅡB	ⅢA	ⅢB
T3	ⅡB	ⅢA	ⅢB	ⅢC
T4	ⅢA	ⅢA	ⅢB	ⅢC
M1a	ⅣA	ⅣA	ⅣA	ⅣA
M1b	ⅣA	ⅣA	ⅣA	ⅣA
M1c	ⅣB	ⅣB	ⅣB	ⅣB

Annex V Pathology Assessment Form

Screening number:	Sex:	Age:	Pathology No:
Department:	Sending physician:	Date of delivery for examination:	Reporting Physician:

Type of specimen sent for examination:

Gross description[1]:

Pathologic diagnosis[2]:

RVT (residual viable tumor cells)[3]: RVT1 (arithmetic mean)[4]= %

RVT2 (Weighted average)[5]= %

RVT3 (Arithmetic mean)[6]= %

RVT4 (Weighted average)[7]= %

Lymph nodes[8]:

Neoadjuvant immunotherapy related treatment response assessment:

Inflammatory lesions: mild moderate severe

Tertiary lymphoid structures[9]: Yes No

Neovascularization[10]: Yes No

Other

MPR (Major Pathologic Remission)[11]: Yes No

PCR (complete pathological remission)[12]: yes no

Staging: ypT N M

Remarks:

Signature of evaluator:________________ **Date of evaluation:**

Notes:

1. Note assessment of necrosis and percentage of residual tumor;
2. Same as routine report;
3. RVT (Residual viable tumor, residual viable tumor cells);
4. RVT1 The arithmetic mean number of residual surviving tumor cells in the primary tumor slice is calculated only, RVT1=(A slice+ ......+N slice percentage of residual surviving tumor cells)/N;
5. RVT2 Calculate the weighted mean number of residual viable tumor cells in the primary tumor section only, RVT2=Percentage of viable tumor in A slice × area of tumor bed in A slice/Total area of N slice+ ......+Percentage of viable tumor in N slice×area of tumor bed in N slice/Total area of N slice;
6. RVT3 Calculate the arithmetic mean number of residual surviving tumor cells in the primary tumor and lymph node sections, RVT3 = (A slice+ ...... + N slice percentage of residual surviving tumor cells)/N;
7. RVT4 Calculate the weighted mean number of residual viable tumor cells in the primary tumor and lymph node sections, RVT4= Percentage of viable tumor in A slice × tumor bed area in A slice/Total area of N slice+ ......+ Percentage of viable tumor in N slice × tumor bed area in N slice/Total area of N slice;
8. Similar to routine reports, the presence or absence of treatment response

needs to be indicated;

9. Ectopic lymph node-like structures appearing within the tumor, which may be accompanied by the formation of germinal centers and include predominantly T cells, B cells, dendritic cells, and high endothelial venules, among other components;
10. Small neovascularization during repair of tissue damage;
11. MPR is defined as the percentage of residual live tumor cells in the tumor bed after neoadjuvant therapy ≤ 10%, regardless of the presence or absence of residual live tumor cells in the lymph nodes (i.e., RVT1 and RVT2 are used as the standard, and if the results of the two are not assessed in the same way, RVT1 is used as the standard)
12. PCR was defined as the absence of residual live tumor cells both within the tumor bed and in the lymph nodes after neoadjuvant therapy.

References

1. *Almasi Z., H. Salehiniya, N. Amoori, et al. Epidemiology Characteristics and Trends of Lung Cancer Incidence in Iran.[J]. Asian Pac J Cancer Prev,2016,17(2),557-62. doi:10.7314/apjcp.2016.17.2.557.*
2. *Zheng RS, Sun KX, Zhang SW, et al. Report of cancer epidemiology in China, 2015[J]. Chin J Oncol, 2019, 41(1):19-28. doi:10.3760/cma.j.issn.0253-3766.2019.01.008.*
3. *Früh M, Betticher DC, Stupp R, et al. Multimodal Treatment in Operable Stage III NSCLC: A Pooled Analysis on Long-Term Results of Three SAKK trials (SAKK 16 /96, 16/00, and 16/01). J Thorac Oncol. 2019 Jan;14(1):115-123.*
4. *McElnay P, Lim E. Adjuvant or neoadjuvant chemotherapy for NSCLC. J Torac Dis, 2014, 6 Suppl 2: S224-S227.*
5. *Pignon JP, Tribodet H, Scagliotti GV, Douillard JY, Shepherd FA, Stephens RJ, Dunant A, Torri V, Rosell R, Seymour L, Spiro SG, Rolland E, Fossati R, Aubert D, Ding K, Waller D, Le Chevalier T; LACE Collaborative Group. Lung adjuvant cisplatin evaluation: a pooled analysis by the LACE Collaborative Group. J Clin Oncol. 2008 Jul 20;26(21):3552-9.*
6. *Li N,Ying JM,Tao XL,et al. Efficacy and Safety of Neoadjuvant PD-1 Blockade with Sintilimab in Resectable Non-Small Cell Lung Cancer[EB/OL].WCLC 2019, abstract JCSE01.10.*
7. *Rusch V W, Chaft J E, Johnson B, et al. Neoadjuvant atezolizumab in resectable non-small cell lung cancer (NSCLC): initial results from a multicenter study (LCMC3)[J]. Journal of Clinical Oncology, 2018, 36, 8541. DOI: 10.1200/JCO. 2018.36.15_suppl.8541.*
8. *Jia XH, Xu H, Geng LY, Jiao M, Wang WJ, Jiang LL, Guo H. Efficacy and safety of neoadjuvant immunotherapy in resectable nonsmall cell lung cancer: a meta- analysis. Lung Cancer. 2020 Sep;147:143-153. doi: 10.1016/j.lungcan.2020.07.001. Epub 2020 Jul 10.*
9. *Motz G T , Coukos G . The parallel lives of angiogenesis and*

immunosuppression: cancer and other tales[J]. Nature Reviews Immunology, 2011, 11(10):702.

10. *Jain RK. 2014 Cancer Cell 26(5):605-622. Jain RK. Antiangiogenesis strategies revisited: from starving tumors to alleviating hypoxia. Cancer Cell. 2014 Nov 10;26(5):605-22. doi: 10.1016/j.ccell.2014.10.006. Epub 2014 Nov 10.*
11. *Yang Y, Li L, Jiang Z, Wang B, Pan Z. Anlotinib optimizes anti-tumor innate immunity to potentiate the therapeutic effect of PD-1 blockade in lung cancer. Cancer Immunol Immunother. 2020 Jun 23.*
12. *Han B., T. Chu, R. Zhong, et al. P1.04-02 Efficacy and Safety of Sintilimab with Anlotinib as First-Line Therapy for Advanced Non-Small Cell Lung Cancer (NSCLC). [J]. Journal of Thoracic Oncology,2019,14(10).*
13. *Dahan R, Sega E, Engerlhardt J, et al. FcγRs Modulate the Anti-tumor Activity of Antibodies Targeting the PD-1/PD-L1 Axis. Cancer Cell. 2015 Sep 14; 28 (3) :285-95.*
14. *Boutros C, Tarhini A, Routier E, et al. Safety profiles of anti-CTLA4 and anti-PD-1 antibodies alone and in combination. Nat Rev Clin Oncol. 2016 13(8). 473-86.*
