# Supplementary material for: Perioperative penpulimab-based combination therapy in patients with resectable non-small cell lung cancer (ALTER-L043): an open-label, multicenter, randomized, phase II trial
Source: Signal Transduct Target Ther. 2026 Jan 16;11:21. doi: 10.1038/s41392-025-02544-w (PMC12811312; doi:10.1038/s41392-025-02544-w)
Supplement: Supplementary file 3 — SAP [file 41392_2025_2544_MOESM3_ESM.docx]

An Exploratory Phase II Clinical Study of a Penpulimab-Based Combination Regimen for Neoadjuvant/Adjuvant Treatment of Resectable Locally Advanced Non-Small Cell Lung Cancer

Statistical Analysis Plan

Principal Investigator: Prof. Changli Wang

Statistical unit: Tianjin Cancer Hospital

Co-organizer: Chia Tai Tianqing Pharmaceutical Group Co., Ltd.

Version No.: 3.0

Version Date: September 7, 2022

The ownership of the information contained in this document belongs to Chia Tai Tianqing Pharmaceutical Group Co., Ltd., and the information contained herein may not be copied or disclosed without the written consent of Chia Tai Tianqing Pharmaceutical Group Co., Lt

**Program signature page**

**Signature of Statistical Unit**

I have read in detail " An Exploratory Phase II Clinical Study of a Penpulimab-Based Combination Regimen for Neoadjuvant/Adjuvant Treatment of Resectable Locally Advanced Non-Small Cell Lung Cancer" of the statistical analysis plan (version number: 3.0), and agreed to perform statistical analysis of the trial results in accordance with the plan.

Statistical analysis unit: Tianjin Cancer Hospital

|  | | | | |
| --- | --- | --- | --- | --- |
| Principal person in charge (in print) |  | Principal person in charge (signature) |  | Date of signature (dd/mm/yyyy) |

**Research Program Version Update Record**

| Version No. | Version Date |
| --- | --- |
| 1.0 | December 21, 2020 |
| 2.0 | March 20, 2021 |
| 2.1 | May 21, 2021 |
| 3.0 | September 7, 2022 |
|  |  |
|  |  |
|  |  |
|  |  |
|  |  |
|  |  |
|  |  |
|  |  |

Note: Modifications are detailed in the Program Modifications and Approvals section.

# Program Summary

| Study Title | An Exploratory Phase II Clinical Study of Penpulimab-Based Combination Regimen for Neoadjuvant/Adjuvant Treatment of Resectable Locally Advanced Non-Small Cell Lung Cancer |  |
| --- | --- | --- |
| Study Number | ALTER-L043 |  |
| Version and Date | Version 3.0 Date September 7, 2022 |  |
| Applicant | Tianjin Cancer Hospital |  |
| Co-organizer | Chia Tai Tianqing Pharmaceutical Group Co. |  |
| Nature of study | Exploratory study |  |
| Subjects | Stage IIB-IIIB(N2), radically resectable, driver gene-negative NSCLC |  |
| Purpose of the study | Primary objective: To observe the primary efficacy of Penpulimab-based combination regimen neoadjuvant/adjuvant for the treatment of resectable locally advanced NSCLC. |  |
|  | Secondary Objective: To observe the safety of the Penpulimab-based combination regimen for the neoadjuvant/adjuvant treatment of resectable locally advanced NSCLC. |  |
| Study Endpoints | Primary study endpoint   - Major Pathologic Remission Rate (MPR) as assessed by the investigator, i.e., the percentage of residual surviving tumor cells in the tumor bed in the postoperative specimen≤ 10% based on the Pathologic Remission Assessment Criteria.   Secondary study endpoints   - Complete pathological remission pCR [defined as the absence of residual tumor cells (including lymph nodes free of tumor remnants) in postoperative tumor tissue specimens, based on the Pathological Remission Assessment Criteria], preoperative objective remission rate (ORR), 1-year event-free survival (1-y EFS%), event-free survival (EFS), overall survival (OS), as assessed by the investigator - Safety: adverse events (AE) |  |
| Study Design. | This study was designed to evaluate and observe the primary efficacy and safety of the Penpulimab-based combination regimen neoadjuvant/adjuvant for the treatment of resectable locally advanced NSCLC  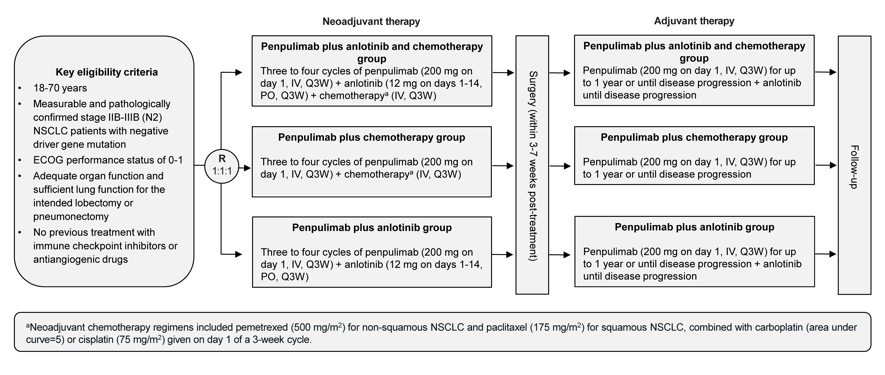  This was an exploratory study, and a total of 90 patients were enrolled and randomly assigned to group A/B/C, with 30 patients in each group.  Group A:  Neoadjuvant therapy (amlotinib+ Penpulimab, 4 cycles; chemotherapy 3-4 cycles): amlotinib, 12 mg, po, qd, continuous oral for 2 weeks with one week off, 3 weeks for 1 cycle; Penpulimab, 200 mg, iv, d1, 3 weeks for 1 cycle; chemotherapy (3-4 cycles): patients with non-squamous carcinoma chose pemetrexed+carboplatin/cisplatin (pemetrexed Pemetrexed,500mg/m^(2) ,^iv,d1,3 weeks for 1 cycle; Cisplatin,75mg/m^(2) ,^iv,d1,3 weeks for 1 cycle; Carboplatin AUC 5,iv,d1,3 weeks for 1 cycle), squamous carcinoma patients chose paclitaxel+carboplatin/cisplatin, (Paclitaxel,175mg/m^(2) ,^iv,d1,3 weeks for 1 cycle; Cisplatin,75mg/m ^(2) ,^iv,d1,3 weeks as a cycle; carboplatin AUC 5,iv,d1,3 weeks as a cycle) (Carboplatin dose (mg): set AUC 5mg/ml/min× [urinary creatinine clearance (ml/min)+25],AUC:area under the curve).  Surgery was performed 3-7 weeks after the final dose of amlotinib, and was evaluated by the investigator for surgery  Adjuvant therapy 4-6 weeks postoperatively as assessed by the investigator  Adjuvant therapy: anilotinib, 12 mg, po, qd, 2 consecutive oral weeks off one week, 1 cycle of 3 weeks (until PD); Penpulimab, 200 mg, iv, d1, 1 cycle of 3 weeks (until PD or up to 1 year of treatment);  Group B:  Neoadjuvant therapy (Penpulimab, 4 cycles; chemotherapy 3-4 cycles):  Penpulimab, 200 mg, iv, d1, 1 cycle in 3 weeks; chemotherapy (3-4 cycles): Patients with non-squamous cancers chose pemetrexed + carboplatin/cisplatin (pemetrexed, 500 mg/m^(2) ,^iv, d1, 1 cycle in 3 weeks; cisplatin, 75 mg/m^(2) ,^iv, d1, 1 cycle in 3 weeks; carboplatin AUC 5, iv, d1, 3 week for one cycle) and paclitaxel + carboplatin/cisplatin for patients with squamous carcinoma (paclitaxel,175mg/m^(2) ,^iv,d1,3 weeks for one cycle; cisplatin,75mg/m^(2) ,^iv,d1,3 weeks for one cycle; carboplatin AUC 5,iv,d1,3 weeks for one cycle) (Carboplatin dose (mg): the set AUC 5mg/ml/min× [urine creatinine clearance (ml/min)+25],AUC:area under the curve).  Surgery was performed 3-7 weeks after the final administration of Penpulimab and was evaluated by the investigator to be performed  Adjuvant therapy 4-6 weeks postoperatively as assessed by the investigator  Adjuvant therapy: penpulimab, 200 mg, iv, d1, 1 cycle of 3 weeks (until PD or up to 1 year of treatment);  Group C:  Neoadjuvant therapy (Anrotinib+ Penpulimab, 4 cycles): anrotinib, 12 mg, po, qd, continuous oral for 2 weeks off for one week, 3 weeks for 1 cycle; Penpulimab, 200 mg, iv, d1, 3 weeks for 1 cycle;  Surgery 3-7 weeks after final dose of amlotinib, as evaluated by the investigator  Adjuvant therapy 4-6 weeks postoperatively as assessed by the investigator  Adjuvant therapy: amlotinib, 12 mg, po, qd, 2 consecutive weeks by mouth with one week off, 1 cycle of 3 weeks (until PD); Penpulimab, 200 mg, iv, d1, 1 cycle of 3 weeks (until PD or up to 1 year of treatment);  All chemotherapeutic agents (carboplatin, cisplatin, pemetrexed, and paclitaxel) are required to be nationally consistently evaluated drugs. |  |
| Planned number of patients enrolled | 90 |  |
| Principal Investigator | Prof. Changli Wang |  |
| Organization in charge of the clinical trial | Tianjin Cancer Hospital |  |
| Patient Screening Criteria | **General Inclusion Criteria:**   - Subjects voluntarily enroll in the study, sign the informed consent form, good compliance, cooperate with follow-up visits - Age≥ 18 years and≤ 70 years at the time of signing the informed consent form, male or female; - Eastern Cooperative Oncology Group (ECOG) physical status score of 0 or 1; - Expected survival of not less than 12 weeks; - Male and female patients of childbearing age agree to use a reliable method of contraception before entering the trial, during the study and until 8 weeks after discontinuation. - Consent to collect tumor histology specimens needed for the study and apply them to the study; - Patients who agree to undergo radical surgical treatment; - Patients who, in the judgment of the specialist, have no contraindications to surgery   **Disease-related inclusion criteria:**   - Patients with non-small cell lung cancer diagnosed by pathologic histology or cytology (according to the WHO 2015 classification); and patients with radically resectable stage IIB-IIIB (N2) non-small cell lung cancer (as judged by the International Association for the Study of Lung Cancer (IASLC) Manual of Thoracic Tumor Staging, 8th edition); and with primary or lymph node metastasis testing clearly EGFR/ALK/ROS1 negative (patients with squamous carcinoma will have the need for genetic testing at the discretion of the investigator); - Subjects with primary non-small cell lung cancer not previously treated with surgery, chemotherapy, radiation therapy, and biologic therapy; - Patients with resectable lesions as judged by the investigator, with clinical stage IIB-IIIB (N2), who can obtain sufficient tumor histology specimens (non-cytology) for molecular marker analysis; - Evaluable disease with at least one single diameter measurable lesion with a longest diameter of ≥ 10 mm measured by spiral CT according to the criteria for the evaluation of the efficacy of solid tumors (RECIST 1.1) - Subjects must have adequate lung function for the intended lung resection.   **Normal major organ function should meet the following criteria:**   - Pulmonary Ventilation Function Test, FEV1≥ 1.5L, or FEV1≥ 800 ml after anticipated lobectomy/total lung resection; - Standard blood tests (no transfusion of blood or blood products within 14 days, not corrected with G-CSF and other hematopoietic stimulating factors):  1. Hemoglobin (HB) ≥90g/L 2. Absolute neutrophil count (ANC) ≥1.5× 10^9^/L 3. Platelets (PLT) ≥80× 10^9^/L;  - Biochemical tests need to meet the following indicators:  1. Total bilirubin (TBIL) ≤ 1.5 times the upper limit of normal (ULN); 2. Alanine aminotransferase (ALT) and aspartate aminotransferase AST≤ 2.5× ULN; 3. Serum creatinine (Cr) ≤ 1.5× ULN or creatinine clearance (CCr) ≥ 60 ml/min.  - International normalized ratio (INR) of prothrombin time ≤ 1.5 and partial thromboplastin time (APTT) ≤ 1.5 times the upper limit of normal in patients who have not received anticoagulation therapy. Patients receiving full or parenteral anticoagulant therapy may be admitted to clinical trials as long as the dose of anticoagulant has been stable for at least 2 weeks prior to entry into a clinical study and the results of coagulation assays are within the local therapeutic limits; - Women of childbearing potential (15～ 49 years of age) must have had a negative urine pregnancy test within 7 days prior to initiating treatment.   **Exclusion Criteria:**   - Large cell carcinoma and mixed cell lung cancer; - Patients who, in the judgment of the investigator, have a high probability of fatal hemorrhage due to tumor invasion of vital blood vessels during subsequent studies; or who have significant cavitary or necrotic tumors in the lungs; - Any systemic anticancer therapy, including cytotoxic drug therapy, immunologic drug therapy, or experimental therapy, for NSCLC; - Have had localized radiotherapy for NSCLC; - Patients who have had a cancer other than NSCLC in the five years prior to the start of treatment in this study. Excluding cervical carcinoma in situ, cured basal cell carcinoma, and bladder epithelial tumors [including Ta and Tis]; - Patients with prior use of amlotinib and other anti-angiogenic agents; - Patients with prior use of Penpulimab, or other anti-PD-1, anti-PD-L1, anti-CTLA-4 antibodies, and any other antibody or drug therapy targeting the T-cell co-stimulatory or checkpoint pathways, such as ICOS or agonists (e.g., CD40, CD137, GITR, OX40, etc.); - Hypersensitivity to amlotinib or Penpulimab or any component of the chemotherapeutic agent; - Patients with multiple factors that interfere with oral administration of medications (e.g., inability to swallow, chronic diarrhea, and intestinal obstruction); and - Patients with the presence of any severe and/or uncontrolled medical condition, including:  1. Patients with suboptimally controlled blood pressure (systolic blood pressure ≥ 150 mmHg and diastolic blood pressure ≥ 100 mmHg); 2. Patients with class I or greater myocardial ischemia or myocardial infarction, arrhythmias (including QTc≥ 480ms) and class ≥2 congestive heart failure (New York Heart Association (NYHA) classification); 3. Abnormal coagulation (INR > 1.5 or prothrombin time (PT) > ULN + 4 seconds or APTT > 1.5 ULN), bleeding tendency or undergoing thrombolytic or anticoagulant therapy; Note: The use of low-dose heparin (daily dosage of 0.6 million in adults) or low-dose aspirin (daily dosage ≤ 100 mg) is permitted for prophylactic purposes, provided that the international normalized ratio of the prothrombin time (INR) ≤ 1.5 is used. ~12,000 U) or low-dose aspirin (≤ 100 mg daily) for prophylactic purposes. 4. Active or uncontrolled serious infections; 5. Cirrhosis, decompensated liver disease, active hepatitis or chronic hepatitis requiring antiviral therapy; 6. Renal failure requiring hemodialysis or peritoneal dialysis; 7. History of immunodeficiency, including being HIV-positive or having other acquired, congenital immunodeficiency diseases, or a history of organ transplantation; 8. Poorly controlled diabetes mellitus (fasting blood glucose (FBG) > 10 mmol/L); 9. Those with routine urinalysis suggestive of urinary protein ≥++ and confirmed 24-hour urine protein quantification > 1.0 g; 10. Patients with seizures and requiring treatment; 11. Prolonged unhealed wounds or fractures, etc; 12. Clinically significant hemoptysis (>50 ml per day) within 2 weeks prior to enrollment; or clinically significant bleeding symptoms or a definite bleeding tendency, such as gastrointestinal bleeding, bleeding gastric ulcer, fecal occult blood++ and above at baseline, or suffering from vasculitis;  - Pre-existing interstitial lung disease, drug-induced interstitial disease, radiation pneumonitis requiring hormonal therapy, or any clinically evidenced active interstitial lung disease; - Those who have had an arterial/venous thrombotic event within 6 months, such as cerebrovascular accidents (including transient ischemic attacks), deep vein thrombosis, and pulmonary embolism; - Presence of current peripheral neuropathy of ≥ CTCAE degree 2, except as a result of trauma; - Patients requiring total right lung resection; subjects who have had major surgery or severe trauma have had the effects of surgery or trauma resolved for less than 14 days prior to enrollment at ; - Patients who are participating in another clinical study or are less than 4 weeks from the end of treatment in a previous clinical study; - Patients with mixed small cell lung cancer components; - Have received a live or attenuated vaccine within 30 days prior to the first dose of Penpulimab or plan to receive a live or attenuated vaccine during the study period; - Known history of severe hypersensitivity reactions to other monoclonal antibodies; - Pregnant or lactating women; - Prior history of definite neurologic or psychiatric disorders, including epilepsy or dementia; - Patients who, in the judgment of the investigator, may have other factors that may force the mid-term termination of this study, such as other serious illnesses or serious laboratory test abnormalities or concomitant family or social factors that would compromise the safety of the subjects or the collection of trial data and samples. |  |
| Withdrawal Criteria | - - - 1. Subject voluntarily withdraws informed consent at any time;       2. Medical imaging or clinical features suggesting relapse of the disease;       3. Those who are unable to tolerate the toxicity of amlotinib after two dose downward adjustments;       4. Subjects who experience a pregnancy event during the course of the study;       5. The occurrence of any clinical adverse event, laboratory test abnormality, or other medical condition that results in the possibility that the subject may no longer benefit from continued dosing;       6. Subjects found to be ineligible after enrollment;       7. Other reasons why the investigator believes that the trial treatment cannot be continued. |  |
| Criteria for Termination of Study | Subjects must be terminated from study medication upon the occurrence of any of the following (including, but not limited to)   - An unintended, meaningful, or unacceptable risk to the subject is discovered; - A significant failure of the protocol is discovered during the execution of the trial; - The investigational drug/trial treatment is ineffective, or continuation of the trial is pointless; - Extreme difficulty in completing the trial due to, for example, significant lags in subject enrollment or frequent protocol deviations. |  |
| Exclusion Criteria | - Failure to administer the medication at the dose, method, and regimen specified in this study protocol (discontinuation of medication for a cumulative period of more than four weeks in a single dosing cycle will be recorded as a dropout); - Those who were treated with other chemotherapy or experimental drugs other than this protocol during the trial; - Those who did not meet the criteria and were included in error; - Patients not on medication |  |
| Determination of sample size | Assuming an MPR of 42% for each arm, taking historical data (MPR of 19% for atalizumab monotherapy in LCMC3), taking α=0.05, β=0.2, and using the PASS15 software, and after a preset 10% dropout rate, it is proposed to enroll 30 subjects in each arm of the study, for a total enrollment of 90 subjects. |  |
| Study Duration | Anticipated start of trial: 2021 August  Estimated time to completion of enrollment: 2023 Feb.  Anticipated Trial End Date: 2029 05 |  |
| Dosing regimen | - Anrotinib, 12 mg, po, qd, taken orally for 2 consecutive weeks with a one-week break in a 3-week cycle; - Penpulimab, 200 mg, iv, d1, 1 cycle in 3 weeks; - Pemetrexed, 500 mg/m2 , iv, d1, 1 cycle in 3 weeks; - Paclitaxel, 175 mg/m2 , iv, d1, 1 cycle in 3 weeks; - Cisplatin, 75 mg/m2, iv, d1, 1 cycle in 3 weeks; - Carboplatin AUC 5, iv, d1, 3 weeks for 1 cycle;   (Carboplatin dose (mg): set AUC 5 mg/ml/min× [urinary creatinine clearance (ml/min) + 25],AUC: area under the curve). |  |

# 1. Introduction

Protocol No. ALTER-L043 is “An Exploratory Phase II Clinical Study of Penpulimab-Based Combination Regimen for Neoadjuvant/Adjuvant Treatment of Resectable Locally Advanced Non-Small Cell Lung Cancer”. This plan describes the methods of statistical analysis of the efficacy and safety of the ALTER-L043 study and provides templates for graphical presentation of the data.

This statistical analysis plan (SAP) was written based on version 3.0 (version date 07/09/2022) of the study protocol and version 3.0 (version date 07/09/2022) of the corresponding electronic case report form (eCRF). Updates to the protocol and eCRF may cause updates to this plan.

For the description of some specific analysis methods, the statistical analysis plan and protocol may not be consistent, please refer to this statistical analysis plan.

This statistical analysis plan was written with reference to the “guidelines for planning and reporting of data management and statistical analysis in clinical trials of drugs and the biostatistical guidelines for clinical trials of drugs” issued by the National Drug Administration (NMPA, formerly the CFDA), as well as ICH E3 (Structure and content of clinical study reports) and ICH E9 (statistical principles for clinical trials), both of which are issued by the International Council (ICH).

# 2. Overview

## 2.1 Purpose

- To evaluate the efficacy and safety of a Penpulimab-based combination regimen for the neoadjuvant/adjuvant treatment of resectable locally advanced NSCLC.

**Primary Objectives**

- To observe the primary efficacy of the Penpulimab-based combination regimen neoadjuvant/adjuvant treatment of resectable locally advanced NSCLC.

**Secondary objective**

- To observe the safety of Penpulimab-based combination regimen for neoadjuvant/adjuvant treatment of resectable locally advanced NSCLC.

## 2.2 Endpoint

**Primary study endpoint**

- Major Pathologic Remission Rate (MPR) as assessed by the investigator, i.e., the percentage of residual surviving tumor cells in the tumor bed in the postoperative specimen≤ 10% based on the Pathologic Remission Assessment Criteria.

**Secondary study endpoints**

- Complete pathological remission pCR [defined as the absence of residual tumor cells (including lymph nodes free of tumor remnants) in postoperative tumor tissue specimens, based on the Pathological Remission Assessment Criteria], preoperative objective remission rate (ORR), 1-year event-free survival (1-y EFS%), event-free survival (EFS), overall survival (OS), as assessed by the investigator
- Safety: adverse events (AE)

### 2.3 Study design

This study evaluates the efficacy and safety of the Penpulimab-based combination regimen neoadjuvant/adjuvant for the treatment of resectable locally advanced NSCLC, as shown in Figure 1.


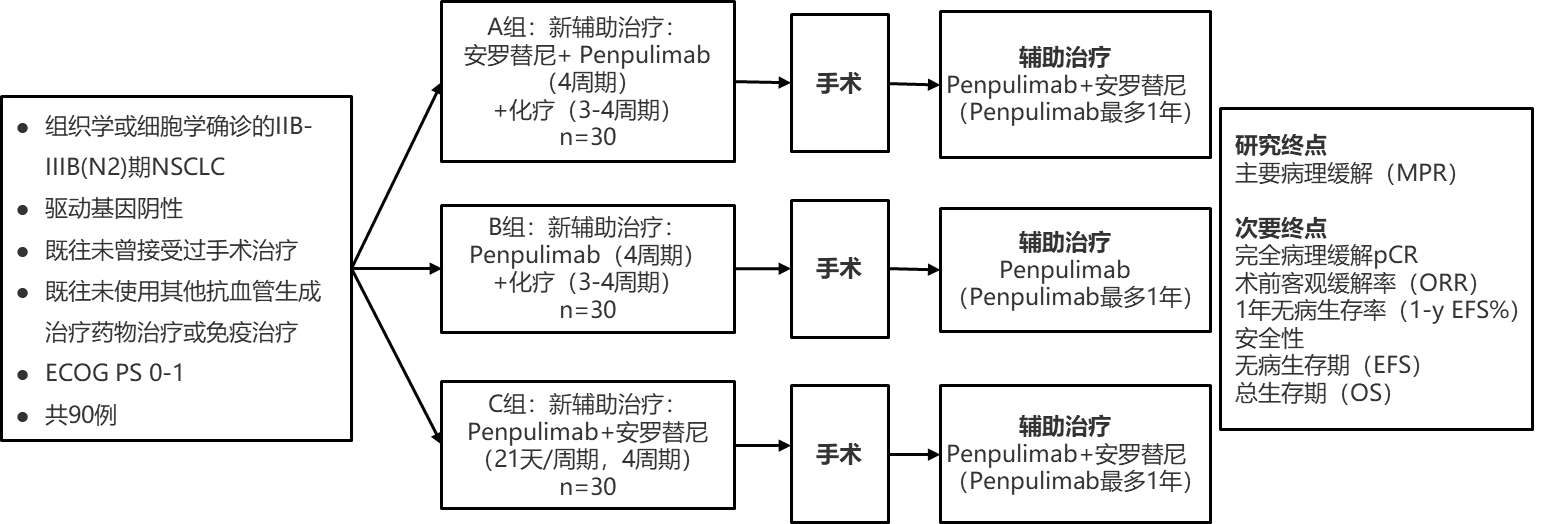


**Figure 1. study design**

This study is a multicenter, randomized, open-label trial planned to be conducted in Tianjin Cancer Hospital and other hospitals. Ninety patients are expected to be enrolled and randomly assigned to groups A/B/C with 30 patients in each group. Initiation is expected to start from March 2021, with an approximate end of enrollment in June 2022.

Study treatment until intolerable toxicity, confirmed disease progression according to RECIST v1.1, death, loss to follow-up, termination of the study by the sponsor, withdrawal of informed consent by the subject, or fulfillment of other termination criteria in the protocol, whichever occurs first.

### 2.4 Sample Size Setting

Assuming that the MPR for each arm of this study is 42%, and taking the historical data (the MPR for atalizumab monotherapy in LCMC3 is 19%), taking α=0.05, β=0.2, and using the PASS15 software, and after a preset dropout rate of 10%, it is proposed to enroll 30 subjects in each arm of the study, with a total of 90 subjects enrolled in the study.

### 2.5 Randomization method

The randomized envelope method of grouping was used in this study. Each patient will correspond to a random serial number after enrollment. Each random serial number will be divided into groups A, B and C according to certain rules.

### 2.6 Drug administration program

**Group A (n=30):**

Neoadjuvant therapy (amlotinib+ Penpulimab, 4 cycles; chemotherapy 3-4 cycles): amlotinib, 12 mg, po, qd, continuous oral for 2 weeks with one week off, 3 weeks for 1 cycle; Penpulimab, 200 mg, iv, d1, 3 weeks for 1 cycle; chemotherapy (3-4 cycles): non-squamous cancer patients chose pemetrexed+carboplatin/cisplatin (Pemetrexed Pemetrexed,500mg/m^(2) ,^iv,d1,3 weeks for one cycle; cisplatin,75mg/m^(2) ,^iv,d1,3 weeks for one cycle; carboplatin AUC 5,iv,d1,3 weeks for one cycle), patients with squamous carcinoma choose paclitaxel+carboplatin/cisplatin (paclitaxel,175mg/m^(2) ,^iv,d1,3 weeks for one cycle; cisplatin,75mg/m^(2) ,^iv,d1,3 weeks as a cycle; carboplatin AUC 5,iv,d1,3 weeks as a cycle) (carboplatin dose (mg): set AUC 5mg/ml/min× [urinary creatinine clearance (ml/min)+25],AUC:area under the curve).

Surgery was performed 3-7 weeks after the final dose of amlotinib, and was evaluated by the investigator for surgery

Adjuvant therapy 4-6 weeks postoperatively as assessed by the investigator

Adjuvant therapy: anilotinib, 12 mg, po, qd, 2 consecutive oral weeks off for one week, 1 cycle of 3 weeks (until PD); Penpulimab, 200 mg, iv, d1, 1 cycle of 3 weeks (until PD or up to 1 year of treatment);

**Group B (n=30):**

Neoadjuvant therapy (Penpulimab, 4 cycles; chemotherapy 3-4 cycles): penpulimab, 200 mg, iv,d1, 1 cycle in 3 weeks; chemotherapy (3-4 cycles): pemetrexed+ Carboplatin/cisplatin for patients with non-squamous carcinoma (pemetrexed,500 mg/m^(2) ,^iv,d1, 1 cycle in 3 weeks; cisplatin,75 mg/m^2,^iv,d1,3 weeks for one cycle; carboplatin AUC 5,iv,d1,3 weeks for one cycle), and patients with squamous carcinoma chose paclitaxel+ Carboplatin/cisplatin, (paclitaxel, 175mg/m^(2) ,^iv,d1,3 weeks for one cycle; cisplatin, 75mg/m^(2) ,^iv,d1,3 weeks for one cycle; carboplatin AUC 5,iv,d1,3 weeks for one cycle) ( Carboplatin dose (mg): set AUC 5mg/ml/min× [urinary creatinine clearance (ml/min)+25],AUC:area under the curve).

Surgery 3-7 weeks after final Penpulimab administration, as assessed by the investigator

Adjuvant therapy 4-6 weeks postoperatively as assessed by the investigator

Adjuvant therapy: Penpulimab, 200 mg, iv, d1, 1 cycle of 3 weeks (until PD or up to 1 year of treatment);

**Group C (n=30):**

Neoadjuvant therapy (amlotinib+ Penpulimab, 4 cycles): amlotinib, 12 mg, po, qd, 2 consecutive oral weeks off for one week, 3 weeks for 1 cycle; Penpulimab, 200 mg, iv, d1, 3 weeks for 1 cycle;

Surgery 3-7 weeks after final dose of amlotinib, as evaluated by the investigator

Adjuvant therapy 4-6 weeks postoperatively as assessed by the investigator

Adjuvant therapy: amlotinib, 12 mg, po, qd, 2 consecutive oral weeks off for one week, 1 cycle in 3 weeks (until PD); Penpulimab, 200 mg, iv, d1, 1 cycle in 3 weeks (until PD or up to 1 year of treatment);

All chemotherapeutic agents (carboplatin, cisplatin, pemetrexed, and paclitaxel) are required to be nationally consistently evaluated drugs.

Patients who experienced complete remission (CR), partial remission (PR), and stable disease (SD) continued dosing until disease progression, intolerable toxicity, or the patient requested discontinuation. Dosing is discontinued in patients with progressive disease (PD).

There are no results from clinical studies in special populations including the elderly, children, pregnant women, or patients with hepatic or renal insufficiency. Analysis of blood concentration data in patients of different ages and genders has shown that patients' blood concentrations are not affected by factors such as age and gender; therefore, dosage adjustments based on age and gender are not recommended.

### 2.7 Dosing cycle

Take 1 capsule (12 mg) of amlotinib hydrochloride on an empty stomach once daily before breakfast. Take 2 consecutive oral doses for 2 weeks and stop for 1 week, i.e. 3 weeks (21 days) as a treatment cycle. In the event of a missed dose, confirm that the time to the next dose is shorter than 12 hours, there will be no make-up dose.

Penpulimab, 200 mg, IV, day 1, 3 weeks (21 days) for one treatment cycle and four cycles of neoadjuvant therapy, up to 1 year of adjuvant therapy.

Chemotherapy (3-4 cycles): pemetrexed, 500 mg/m^(2) ,^iv,d1, one cycle in 3 weeks; cisplatin, 75 mg/m^(2) ,^iv,d1, one cycle in 3 weeks; carboplatin AUC 5,iv,d1, one cycle in 3 weeks; paclitaxel, 175 mg/m^(2) ,^iv,d1, one cycle in 3 weeks.

In subjects with disease control (CR+PR+SD) and tolerable adverse effects, amlotinib was continued until disease progression occurred, the subject voluntarily withdrew informed consent, and intolerable toxicity occurred. Dosing was terminated when the subject was deemed unsuitable by the investigator for continued dosing or was evaluated as PD according to RECIST 1.1 criteria.

# 3. Efficacy Evaluation and Analysis

During the neoadjuvant treatment phase, imaging assessments were performed once on Day 21 of Cycle 2 and once within 7 days prior to surgery. Postoperative MPR evaluation was performed; postoperative adjuvant treatment phase was evaluated every 12 weeks (up to 1 year postoperatively) and every 24 weeks starting in the second year (up to 5 years postoperatively); all subjects were required to keep all imaging data.

The expected duration of study treatment for each subject will continue until imaging-confirmed tumor progression, provided that none of the following conditions occur, such as withdrawal of informed consent by the subject, drug toxicities that are not tolerable, or the investigator's opinion that further testing is not appropriate.

### 3.1 Biomarker analysis

PD-L1 expression will be measured once during the screening period and once after surgery, and data from the different PD-L1 expression groups will be analyzed to compare the efficacy of the treatment.PD-L1 will be detected by immunohistochemistry using the IHC 22C3 PharmDx (DAKO) assay.PD-L1 will be detected by immunohistochemistry using the IHC 22C3 PharmDx (DAKO) assay.

### 3.2 Survival Follow-up

Subjects entered the follow-up period after discontinuing trial administration; subjects remained in the trial period and should be followed up further until death or loss to follow-up. All subjects will be followed up 5 years after surgery at a frequency of every 48 weeks. Follow-up can be done by telephone by asking the subject himself/herself, his/her family or local physician.

### 3.3 Follow-up of Adverse Events

Adverse events that have not recovered by the time the study drug is discontinued should be followed up and a final evaluation made. All patients should be followed for a 21-day safety visit after final dosing to detect any new adverse events.

# 4. Efficacy evaluation indicators

### 4.1 Main indicators and observation methods

### 4.1.1 Assessment of main efficacy indicators

**Major pathologic remission rate (MPR).**

Defined as ≤10% of residual tumor cells in surgically resected tumor specimens after neoadjuvant therapy. The formula is: residual surviving tumor cell area/(residual surviving tumor cell area+ necrotic area+ stromal tissue area). See Appendix V for specific assessment forms.

### 4.1.2 Assessment of secondary efficacy indicators

**Complete Pathologic Remission Rate (pCR).**

Defined as the absence of residual viable tumor cells within the tumor bed and in the lymph nodes as assessed by the pathological response of postoperative specimens after neoadjuvant therapy. See Appendix V for specific assessment forms.

**Preoperative objective remission rate (ORR):**

Refers to the proportion of patients with a certain amount of tumor shrinkage that is achieved and maintained for a certain period of time, and encompasses both CR and PR cases. Objective tumor remission was assessed using the Solid Tumor Remission Assessment Criteria (RECIST 1.1 criteria). Subjects had to be accompanied by measurable tumor lesions at baseline, and the efficacy assessment criteria were classified as complete remission (CR), partial remission (PR), stable (SD), and progression (PD) according to the RECIST 1.1 criteria.

**1-year event-free survival percentage (EFS%).**

Defined as the proportion of subjects who did not experience imaging-confirmed disease progression, local progression leading to inoperable disease, unresectable tumor, local or distant recurrence, or death due to any cause from the time of initiation of the drug to 1 year as a proportion of the total number of subjects in the group.

**Event-free survival (EFS)**

Defined as the period from initiation of medication until imaging-confirmed disease progression, local progression leading to inoperable disease, unresectable tumor, local or distant recurrence, and death due to any cause.

**Overall Survival (OS):**

Overall survival (OS) was defined as the period from the date of enrollment to the date of death from any cause. Subjects who were alive at the time of the final follow-up visit had an OS that was data censored at the time of the final follow-up visit. The OS of subjects who were lost to follow-up was counted as data censored at the time of last confirmed survival prior to the loss of follow-up. OS for data deletion was defined as the time from enrollment to deletion.

**For safety (SAFETY)** evaluation, refer to Section 9.

# 5. Safety evaluation

### 5.1 Adverse events

Adverse Event (AE) refers to all adverse medical events that occur after a subject receives a test drug, which may manifest as signs and symptoms, disease, or abnormal laboratory tests, but are not necessarily causally related to the test drug. The collection period for AEs in this trial begins when the subject signs the informed consent form and continues until 30 days after the last dose of the drug or the initiation of treatment for a new target indication.

### 5.2 Evaluation of Adverse Events

Criteria for evaluating the nature and severity of adverse events followed the National Cancer Institute's Common Toxicity Criteria [NCI -CTC v5.0].

### 5.3 Documentation of Adverse Events

It is the responsibility of the investigator to collect all AEs (including SAEs) during the protocol-specified AE reporting period and record them on the CRF/eCRF form. When recording AEs, the investigator should use correctly standardized medical terminology and avoid colloquialisms and abbreviations. The time of onset of the AE, the NCI CTCAE v5.0 grading of the highest degree, the time of termination, relevance to the study medication, impact to the study, presence or absence of concomitant therapy, and recovery need to be documented.

Diagnosis vs. signs and symptoms

If a diagnosis already exists, the diagnosis should be recorded on the CRF/eCRF rather than individual signs and symptoms (e.g. record liver failure rather than jaundice, elevated aminotransferases and fluttering tremor). However, if the symptoms and signs cannot be categorized as a single diagnosis at the time of reporting, each individual event should be recorded as an AE on the CRF/eCRF. If the diagnosis is later established, it should be updated on the CRF/eCRF to document the diagnosis.

Adverse events secondary to other events

In general, AEs secondary to other events (e.g., caused by other events or clinical sequelae) should be documented as primary events unless the secondary event is of greater severity or is an SAE; however, clinically significant secondary events should be documented as separate AEs on the CRF/eCRF if they do not occur at the same time as the primary event, or separately if the association between the events is not clear. record the primary event and the secondary event.

Persistent, intermittent, or separate adverse events (frequency of adverse events)

A persistent AE (continuous AE) is an AE that persists throughout the course without remission, e.g., an upper respiratory infection that lasts 5 days. This type of AE should be recorded with only one entry on the CRF/eCRF. For severity assessment, the most severe of the event over the full course should be recorded.

An intermittent AE is an AE in which symptoms, signs, or laboratory markers change or resolve throughout the course of the event, but no clinically significant outcome occurs, e.g., nausea and vomiting that lasts for multiple days, with relative remission during the course of the event, or hypertension in a patient who has had intermittent remissions in multiple blood pressure tests, but the course of the hypertension has been relatively continuous. Such AEs may be recorded as a single event on the CRF/eCRF. For intensity assessment, the most severe of the full course of the event should be recorded.

A single AE (Single AE) is an AE that logically can only occur in isolation or that occurs independently only once during the trial, e.g., an incidental fall of the patient during medication administration; vomiting by the patient that occurs only once during the trial. Such AEs are recorded only once on the CRF/eCRF.

It should be noted that if there has been a clinically significant recovery from the above AE, but the same AE occurs later and the latter is not considered to be in continuity with the former in terms of course, the occurrence of the event should be recorded separately on the CRF/eCRF.

Abnormal laboratory tests or abnormal vital signs

All laboratory test results may be documented on the CRF Laboratory Results page. Not all laboratory test abnormalities/abnormal vital signs should be documented as an AE. It is the responsibility of the investigator to review all abnormal laboratory results and abnormal vital signs and make a medical judgment as to whether they should be documented as an AE. Any of the above abnormalities should be documented as an AE when clinically significant, e.g., when one or more of the following conditions are met:

- Accompanying clinical symptoms
- Result in a change in study medication (e.g., dose adjustment, temporary or permanent discontinuation)
- Requires medical intervention or change in combination therapy (e.g., addition, suspension, discontinuation, or other change in combination medication, treatment, or therapy)
- Clinically significant in the judgment of the investigator

If clinically significant laboratory test abnormalities or vital sign abnormalities are symptomatic of a disease or syndrome (e.g., elevated ALT/AST and blood bilirubin due to hepatic impairment), record the diagnosis (i.e., hepatic impairment) only on the Adverse Events form of the CRF/eCRF. Conversely, record an abnormal laboratory test or abnormal vital signs on the Adverse Events form of the CRF/eCRF and indicate whether the test value is above or below the normal range. If the abnormal laboratory test or abnormal vital sign has a standard clinical term associated with it, the clinical term should be recorded on the CRF/eCRF (e.g., an elevated potassium level of 7.0 mmol/L should be recorded as "hyperkalemia").

Death

When recording a death, if there is an AE that led to the death, record the AE that led to the death using a single medical concept in the CRF/eCRF and report the event as an SAE; if the cause of the death is unknown, record the "Unexplained Death" in the AE table of the CRF/eCRF and report the "Unexplained Death" as an SAE first. If the cause of death is unknown, record "Unexplained Death" on the AE form of the CRF/eCRF and report the event as an SAE, then investigate the exact cause of death and update the record and SAE report when the cause of death is known.

Pre-existing medical conditions

Symptoms/signs that were present during the screening period of the trial should be recorded as AEs only if there is an exacerbation of the severity, frequency, or nature of the symptoms/symptoms (other than a worsening of the disease condition under study) after entry into the trial. Changes in status relative to previous status should be documented, e.g., "increased frequency of headache," "worsening of hypertension," etc.

Hospitalization, prolonged hospitalization

A hospitalization or prolonged hospital stay should not be reported as an SAE if it is: 1) planned in accordance with protocol requirements (e.g., for medication administration, efficacy assessment, etc.); or 2) due to a pre-existing medical condition that has not changed since participation in the study, such as elective surgery or treatment scheduled prior to participation in the study, and then hospitalized after participation in the study to receive the surgery or treatment, if this is not considered to be an adverse event. considered an adverse event. However, if the condition of a pre-existing medical condition worsens in the study (e.g., surgery or treatment is performed earlier than originally planned), then hospitalization for surgery or treatment due to the worsening of the medical condition will require hospitalization for surgery or treatment, and the worsening of the condition will be considered an SAE.

Surgery

If the condition for which the surgery was performed is clearly identified, the condition should be recorded as the AE, not the surgery itself (e.g., if the patient underwent an inguinal hernia repair, "inguinal hernia" should be recorded, not "inguinal hernia repair"); however, if the reason for the surgery is not clear, the name of the surgery may be recorded as the AE. However, if the reason for the procedure is unclear, the procedure name may be recorded as AE (e.g., if the patient underwent an exploratory laparotomy, "exploratory laparotomy" may be recorded as AE).

Pregnancy

The Investigator should be notified immediately if a pregnancy occurs in a female subject or a female partner of a male subject during the clinical trial. The investigator should report the pregnancy to the sponsor by completing the Serious Adverse Event Report Form within 24 hours of learning of the pregnancy event, and follow-up should continue until the pregnancy is terminated (e.g., termination of pregnancy, labor and delivery) and the results reported to the sponsor. If a female subject becomes pregnant, the investigational drug should be discontinued immediately and the investigator should discuss with the subject the risks of continuing the pregnancy and the possible effects on the fetus.

In the event of an induced/spontaneous abortion, termination of pregnancy for medical reasons, or congenital anomalies or malformations of the fetus/neonate during pregnancy, this is considered an SAE and will need to be documented and reported in accordance with the timeframe requirements for SAEs.

Disease progression

If an event occurs that is unequivocally consistent with the expected pattern of progression of the primary tumor, it should not be considered an AE. hospitalization due solely to the progression of that disease should also not be considered an SAE. if the symptoms are not unequivocally due solely to the progression of the disease or do not coincide with the expected pattern of progression of the tumor, the associated clinical symptoms may be documented as an AE, and a SAE should be reported if it is consistent with an SAE.

### 5.4 Follow-up of adverse events

Investigators should follow all AEs until any of the following occurs.

- The AE resolves or improves to baseline levels;
- The investigator confirms that no further improvement is expected;
- The patient dies;
- The patient has been lost to contact;
- The investigator confirms that the AE is not related to the study treatment;
- The patient begins a new anticancer treatment;
- Clinical or safety data are no longer being collected, or the database is finally closed.

The final outcome of each AE (including the date of AE remission or death) must be recorded on the CRF/eCRF.

### 5.5 Criteria for determining drug-adverse event association

The investigator should make an assessment of the possible association between the adverse event and the test drug, which can be determined in the manner listed in the table with reference to the following five categories of criteria.

(1) Whether there is a reasonable chronological sequence between the administration of the drug and the appearance of the adverse reaction;

(2) Whether the reaction is consistent with the types of adverse reactions known to occur with the drug;

(3) Reduction or disappearance of the reaction after discontinuation or reduction of the drug;

(4) Whether the same reaction recurs after reuse of the suspected drug;

(5) Whether the reaction can be explained by the effects of the combined drug, the progression of the patient's condition, or other therapeutic measures;

**Table 10.5.1 Adverse Event-Drug Relationship Determination Forms**

|  | 1 | 2 | 3 | 4 | 5 |
| --- | --- | --- | --- | --- | --- |
| Definitely relevant. | ＋ | ＋ | ＋ | ＋ | -Likely. |
| Most likely. | ＋ | ＋ | ＋ | Most likely. | -Likely? |
| Possibly. | ＋ | -Possibly. | ±Possibly. | What's going on? | ±? |
| Maybe not. | -It's probably not relevant. | -It's probably not. | ±What's the matter? | What? - What? | ±? |
| Definitely not. | -I'm sure it's not. | -It's not. | It's definitely not-- | -It's not. | - |

Note: + means yes, - means no,± means difficult to confirm or deny, ? means the situation is unknown.

Adverse reactions were counted as those that were definitely related, probably related, probably related, or probably unrelated, and the incidence rate of adverse reactions was calculated accordingly.

### 5.6 Serious adverse events

Adverse events are classified as serious adverse events when one or more of the following criteria are met: death, life-threatening (e.g., immediate risk of death), resulting in hospitalization or prolonged hospitalization, permanent or severe disability, congenital deformity or defect, and some medical events that do not result in death, life-threatening injury, or hospitalization, but which are judged by the physician to be potentially harmful to the patient or to require medication or surgery, are also considered to be serious adverse events. A medical event that, in the judgment of the physician, may be harmful to the patient or require medication or surgical intervention to avoid the above is also considered a serious adverse event. The occurrence of pregnancy in the patient or his/her spouse is reported as a Serious Adverse Event to the relevant unit at .

Disease progression (including signs and symptoms of progression) should not be reported as a Serious Adverse Event, but death due to disease progression within the trial or safety reporting period should be reported as a Serious Adverse Event. Hospitalization for signs and symptoms of disease progression should not be reported as a serious adverse event. If the final outcome of the cancer is death during the trial or safety reporting period, then the event leading to death must be reported as a Serious Adverse Event.

### 5.7 Handling of Serious Adverse Events

Any Serious Adverse Event that occurs during the course of a clinical trial should be reported in writing by the investigator to the sponsor's Pharmacovigilance Department at the designated email address (AL3818@cttq.com) within 24 hours of notification, and should be followed by a timely, thorough, written follow-up report. For reports involving fatal events, the investigator should provide the sponsor and the ethics committee with other required information such as autopsy reports and final medical reports. Serious adverse events should be analyzed and evaluated immediately upon receipt by the sponsor, including severity, relevance to the test drug, and whether it is an expected event. For suspected and unanticipated serious adverse events, the sponsor should promptly report them to all investigators participating in the clinical trial of the investigational drug, as well as to the clinical trial site and the ethics committee; and the investigators should report suspected and unanticipated serious adverse events provided by the sponsor to the ethics committee. The sponsor should also report suspected and unanticipated serious adverse reactions to the drug regulatory authorities and health authorities.

### 5.8 management of common adverse events

6.5.1 The section provides dosing adjustments and recommendations for some common adverse events of amlotinib hydrochloride capsules. The types of immune-related adverse events triggered by Penpulimab injections and recommendations for treatment can be found at Recommendations for the Management of Immune-Related Adverse Events Caused by Immune Checkpoint Inhibitor Therapy .

# 6. Statistical analysis

The main efficacy index of this study is the MPR, which was analyzed after the last subject had completed the post-surgical tumor tissue sections and obtained the results, and the 95% confidence intervals of the MPR were estimated by the Clopper-Pearson method.

## 6.1 Selection of data for statistical analysis

● Full Analysis Set (FAS set): according to the principle of Intentional Analysis (ITT), the efficacy of all cases who took 1 dose of the drug was analyzed. For case data where the full course of treatment could not be observed, the last observation data was used to carry forward to the trial final result (LOCF).

● Per-protocol Set (PPS Set): all cases that complied with the trial protocol, were compliant, and took at least 2 cycles of medication (subjects who took more than one cycle of medication and had clear imaging evidence of disease progression were also included in the PPS Set), did not take prohibited medications during the trial period, and completed the required fields on the case report form. Missing data were not filled in (imputation). The efficacy of the drug was statistically analyzed for both FAS and PPS.

● Safety Analysis Set (SAS set), all enrolled cases, all patients who have used the trial drug at least once and have a record of the safety of the drug after use belong to the safety analysis set, this data set is used for safety analysis.

## 6.2 Principles and contents of statistical analysis

(1) General principles

All statistical analyses were realized by writing programming language using SAS version 9.4 statistical software, and all statistical tests were conducted using two-sided tests, where a P value of ≤0.05 would be considered statistically significant for the differences tested, and the confidence interval was used with a 95% confidence level.

Baseline data were analyzed by the full analysis set, safety analyses were analyzed using the safety analysis set, and validity analyses were analyzed by the full analysis set and the compliance with the protocol set.

For continuous variables, the number of non-missing subject cases, mean, standard deviation, median, minimum and maximum values will be listed. The number of decimal places for the minimum and maximum values will be consistent with the records in the database. The mean, median, and standard deviation will retain one more decimal place than the original data recorded in the database.

For categorical variables, they will be presented in the form of frequency tables (frequencies and percentages). Percentages will be retained to two decimal places.

Estimation of missing values: no estimation of missing values will be made in the safety evaluation. If the efficacy evaluation is missing due to early withdrawal from the treatment, "unable to evaluate" will be used to participate in the analysis of the objective remission rate and disease stabilization rate, and the missing OS and PFS will be used as the truncated data.

(2) Study population

①Population distribution

Descriptions of the enrollment status, dropout cases, completion of the expected course of treatment, and early discontinuation of the study population: number and percentage of cases. Distribution of cases, statistical description of dropout and exclusion cases one by one: medication use, proposed reasons for withdrawal from the trial, etc.

Distribution of cases in the safety dataset.

Case-by-case descriptions of dropout and early discontinuation cases: medication use, reasons for early withdrawal, etc.

②Protocol violations and deviations

Summarize and describe cases of protocol violations and deviations.

③ monitoring population demographic characteristics, disease characteristics and medical history analysis

(3) Effectiveness analysis

The primary study endpoint of this study was the MPR in the full analysis set (FAS) population as assessed according to RECIST v1.1. The analysis of the primary endpoint was performed after the last subject had completed the post-surgical tumor tissue sectioning and obtained the results. 95% confidence intervals for MPR were estimated using the Clopper-Pearson method.
 Subjects' tumor tissue section pCR as well as 1-year event-free survival percentage (1-y EFS%), objective remission rate (ORR), event-free survival (EFS), safety, and overall survival (OS) based on the investigator's assessment were secondary efficacy metrics for this study. Among them, for the analysis of EFS and OS, the Kaplan-Meier method will be used to estimate the median time and the corresponding 95% confidence intervals will be listed.The analysis of ORR and pCR will be done using the Clopper-Pearson method to estimate the 95% confidence intervals.
 Comparisons with baseline check values will be made using paired t-tests or Wilcoxon signed rank sum tests, depending on the distributional characteristics of the information

(4) Safety evaluation

Safety analysis is mainly based on descriptive statistical analysis methods, and confidence intervals are utilized to present relevant results when necessary.

①Adverse events

Adverse event data, coded according to the current version of MedDRA at the time of the start of coding, will be processed in the statistical analysis.

Summarizes the incidence of adverse events, adverse reactions, adverse events leading to withdrawal, adverse events leading to death, serious adverse events, etc. Incidence of adverse events/reactions= Number of adverse events/reactions in the number of medications administered/number of medications administered x 100%. If multiple adverse events/reactions occurred in the same monitored case, it was recorded as 1 case in the calculation of the incidence rate; when the same AE occurred multiple times in a monitored case, it was recorded as 1 case in the calculation of the incidence rate of that AE.

Adverse events will be summarized in the frequency table by System Organ Classification (SOC) and Preferred Terminology (PT). Calculate the incidence rate by system and signs/symptoms (count of cases: number of monitored cases with at least one occurrence of a particular adverse event).

Severity of adverse events, reactions: multiple occurrences of the same adverse event in the same subject, with the most severe one participating in the severity analysis for that AE.

Provide a list of cases with the time of occurrence of the adverse event and the time of the serious adverse event.

(ii) Vital signs, laboratory test data, etc;

Mean ± standard deviation, maximum, minimum, and median were used to describe the measurements and changes before and after treatment. Vital signs and laboratory indicators were analyzed descriptively, and paired t-tests were used for within-group comparisons.

(iii) Drug exposure and dose adjustment

Mean, standard deviation, maximum, minimum and median were used to describe the amount of drug administered, and dose adjustment and suspension were analyzed.

④Combined medications

The use of combined medications during the study period was summarized, and the frequency of use of each medication.
